# Multilocus genetic and morphological phylogenetic analysis reveals a radiation of shiny South Asian jumping spiders (Araneae, Salticidae)

**DOI:** 10.3897/zookeys.839.28312

**Published:** 2019-01-16

**Authors:** Nilani Kanesharatnam, Suresh P. Benjamin

**Affiliations:** 1 National Institute of Fundamental Studies, Hantana Road, Kandy, Sri Lanka National Institute of Fundamental Studies Kandy Sri Lanka; 2 Department of Zoology, Faculty of Science, Eastern University, Vantharumoolai, Sri Lanka Eastern University Vantharumoolai Sri Lanka

**Keywords:** Chrysillini, cryptic species, ecomorphs, India, monophyly, new genera, parsimony, synapomorphies

## Abstract

This study presents a systematic revision of South Asian members of the Tribe Chrysillini Simon, 1901. Genetic and morphological variations were analysed of a “similar-looking” group of species that were initially identified as members of the jumping spider genera *Chrysilla* Thorell, 1887 and *Phintella* Strand, in Bösenberg and Strand 1906 to determine their phylogenetic relationships. Results suggest that the assessed morphospecies complex constitute of three evolutionary lineages, two previously unrecognised, which are described and diagnosed as two new genera: *Phintelloides***gen. n.** and *Proszynskia***gen. n.** The third lineage, *Phintella*, is sister to these proposed genera. The following new species are described: *Phintelloidesalborea***sp. n.**, *P.brunne***sp. n.**, *P.flavoviri***sp. n.**, *P.flavumi***sp. n.**, *P.orbisa***sp. n.**, *Phintellaargentea***sp. n.**, and *P.jaleeli***sp. n.** Sri Lanka is rich in biodiversity but currently has one of the highest rates of deforestation. Lack of clarity on diversity and distribution of the islands’ biodiversity can lead to underestimations during threat assessments and thus downgrading of conservation needs of individual species.

## Introduction

A very high degree of endemism in several taxonomic groups such as mammals (Bambaradeniya 2006), amphibians and reptiles (Bossuyt et al. 2004; Meegaskumbura et al. 2002; Pyron et al. 2013), birds (Harrison and Worfolk 2011), fish (Pethiyagoda 1991), and plants (Gunatilleke et al. 2005) are observed in Sri Lanka. However, the invertebrates of the island are less well known. If recently conducted studies on Goblin spiders is a yardstick, invertebrates of Sri Lanka might be highly diverse and the island may harbour endemic radiations of species currently lumped together as widely distributed species (Ranasinghe and Benjamin 2016a, b, 2018). This lack of information on diversity and distribution of the islands’ biodiversity may lead to underestimations during threat assessments and thus downgrading of conservation needs of individual species.

Jumping spiders (Salticidae) are small, diurnal hunters remarkable for their excellent vision and various body forms and behaviours (Land 1969; Blest, O’Carroll and Carter 1990). They are known from all non-polar terrestrial ecosystems (Maddison 2015) with more than 6053 species described in 632 genera (World Spider Catalog 2018). The Salticidae is part of the RTA clade, which includes at least half of all known spiders (Blackledge et al. 2009; Fernández et al. 2018; Wheeler et al. 2016).

Recent field work in Sri Lanka yielded a superficially “similar-looking” group of jumping spider that were initially identified as members of the genera *Chrysilla* Thorell, 1887 and *Phintella* Strand, in Bösenberg and Strand 1906, both of which are members of the tribe Chrysillini Simon, 1901. *Chrysilla* and *Phintella* are widely distributed, encompassing numerous species mainly from Indomalayan and Palaearctic regions (Wesołowska 2010; Wesołowska and Wiśniewski 2013; Żabka 2012). Few members of these genera live in other parts of the world; with seven species known from the Afrotropical region (World Spider Catalog 2018). However, detailed drawings of genitalia of our specimens revealed that at least some of our morphospecies might be distinct from two species of *Chrysilla* and *Phintella* previously recorded from Sri Lanka.

To validate our primary assessment, we performed a series of phylogenetic analyses based on both morphological and genetic variations of individuals collected across the island. Based on our results, we describe herein two new genera, *Phintelloides* gen. n. and *Proszynskia* gen. n., with seven new species. We further use these findings to clarify the geographic ranges of these taxa in an effort to facilitate evaluation of the conservation needs of these distinct evolutionary lineages.

## Materials and methods

### Taxon selection

Our selection of outgroup taxa is based on Maddison (2015) and on the assumption that our taxa of interest (the ingroup) are Chrysillines. In our molecular analysis, we included species of Chrysillines and Hasariines genera including *Bristowia*, *Cheliceroides*, *Chinattus*, *Chrysilla*, *Cosmophasis*, *Echeclus*, *Epocilla*, *Hakka*, *Hasarius*, *Habrocestum*, *Helicius*, *Heliophanus*, *Helvetia*, *Icius*, *Menemerus*, *Mexcala*, *Phintella*, *Pseudicius*, and *Siler*. *Idastrandiaorientalis* (Szombathy, 1915), *Thianialatibola* Zhang & Maddison, 2012 were used to root the tree (Table 1). Based on our analysis of the molecular data and availability of material within Sri Lanka, the following outgroup taxa were selected for the morphological analysis: *Hasariusadansoni* (Audouin, 1826), *Habrocestumkodigalaensis* Kanesharatnam & Benjamin, 2016, *Habrocestumhantaneensis* Kanesharatnam & Benjamin, 2016 and *Bristowiagandhii* Kanesharatnam & Benjamin, 2016. Ten exemplars were selected to represent our taxa of interest and constitute the ingroup; details are given in Table (2). *Phintelloidesorbisa* sp. n. is known only from its female holotype and is not part of the molecular work. However, it is included in the morphological analysis.

**Table 1.** Details of exemplars used in this study including GenBank accession numbers and collection localities. * denotes taxa included in the morphological matrix (see Table 2 for details). Collection localities are given if available.

Species	Locality	Specimen No	CO1	28S	18S	GenSeq Nomenclature
* Phintella vittata *	Sri Lanka: Ampara	IFS_SAL_240	KY888758	KY888746	KY888695	genseq-4 COI, 28S, 18S
Phintellavittata*	Sri Lanka: Anuradhapura	IFS_SAL_816	KY888751	KY888728	KY888696	genseq-4 COI, 28S, 18S
* Phintella vittata *	India: Maharashtra	–	KT383680.1	–	–	–
Phintellaargentea*	Sri Lanka: Nuwara Eliya	IFS_SAL_893	KY888750	KY888722	KY888720	genseq-4 COI, 28S, 18S
* Phintella argentea *	Sri Lanka: Loolecondera	IFS_SAL_857	KY888763	KY888727	KY888721	genseq-4 COI, 28S, 18S
* Phintella linea *	–	–	–	JN817066.1	JN816864.1	–
* Phintella cavaleriei *	–	–	–	JN817067.1	JN816865.1	–
* Phintella abnormis *	–	–	–	JN817064.1	JN816862.1	–
* Phintella arenicolor *	–	–	–	JN817065.1	JN816863.1	–
* Phintella aequipeiformis *	Vietnam	–	LC105670.1	–	–	–
Phintellajaleeli*	Sri Lanka: Dambulla	IFS_SAL_262	KY888757	KY888745	KY888698	genseq-4 COI, 28S, 18S
* Phintella jaleeli *	Sri Lanka: Kurunegala	IFS_SAL_167	KY888760	KY888748	KY888697	genseq-4 COI, 28S, 18S
* Phintella bifurcilinea *	Vietnam	–	LC105668.1	–	–	–
* Phintella piatensis *	Philippines: Luzon	–	AY297396.1	–	–	–
* Phintelloides alborea *	Sri Lanka: Dambulla	IFS_SAL_369	KY888783	KY888737	KY888704	genseq-2 COI, 28S, 18S
Phintelloidesalborea*	Sri Lanka: Mihintale	IFS_SAL_814	KY888766	KY888729	KY888715	genseq-4 COI, 28S, 18S
* Phintelloides versicolor *	Vietnam	–	LC105657.1	–	–	–
Phintelloidesflavumi*	Sri Lanka: Galle	IFS_SAL_742	KY888768	KY888730	KY888712	genseq-4 COI, 28S, 18S
Phintelloidesjesudasi*	Sri Lanka: Kurunegala	IFS_SAL_293	KY888753	KY888741	KY888702	genseq-4 COI, 28S, 18S
Phintelloidesbrunne*	Sri Lanka: Nuwara Eliya	IFS_SAL_844	KY888764	KY888723	KY888717	genseq-4 COI, 28S, 18S
* Phintelloides brunne *	Sri Lanka: Gammaduwa	IFS_SAL_281	KY888754	KY888742	KY888701	genseq-2 COI, 28S, 18S
* Phintelloides flavoviri *	Sri Lanka: Galle	IFS_SAL_754	KY888752	KY888724	KY888713	genseq-1 COI, 28S, 18S
Proszynskiadiatreta*	Sri Lanka: Vavuniya	IFS_SAL_861	KY888761	KY888725	KY888719	genseq-4 COI, 28S, 18S
* Proszynskia diatreta *	Sri Lanka: Vavuniya	IFS_SAL_539	KY888774	KY888733	KY888708	genseq-4 COI, 28S, 18S
*Epocilla* sp.	Sri Lanka: Galle	IFS_SAL_730	KY888771	–	MH304355	genseq-4 COI, 18S
* Epocilla aurantiaca *	Sri Lanka: Kurunegala	IFS_SAL_168	KY888759	–	MH304356	genseq-4 COI, 18S
* Pseudicius vulpes *	–	–	JN817279.1	JN817059.1	JN816857.1	–
* Hakka himeshimensis *	–	–	JN817278.1	JN817058.1	–	–
* Helicius chikunii *	–	–	AB924449.1	–	–	–
* Heliophanus cupreus *	Poland: Mielik	–	DQ665756.1	DQ665769.1	DQ665738.1	–
* Menemerus brachygnathus *	Sri Lanka: Kandy	IFS_SAL_270	KY888755	–	–	genseq-4 COI
* Menemerus bivittatus *	–	–	AY297395.1	AY297266.1	–	–
* Icius subinermis *	Solvenia	–	KX039217.1	–	–	–
Helvetia aff. zonata	–	–	AY297394.1	AY297265.1	–	–
* Mexcala elegans *	South Africa: Kwazulu-Natal	–	EU815594.1	EU815479.1	–	–
Silersemiglaucus*	Sri Lanka: Galle	IFS_SAL_731	KY888770	KY888731	KY888711	genseq-4 COI, 28S, 18S
* Siler cupreus *	–		JN817270.1	JN817051.1	–	–
Chrysillavolupe*	Sri Lanka: Ballagola	IFS_SAL_443	MG910461	MG883389	MG883392	genseq-4 COI, 28S, 18S
*Cosmophasis* sp.	Sri Lanka: Ethagala	IFS_SAL_522	KY888776	–	MH304353	genseq-4 COI
*Cosmophasis* sp.	Sri Lanka: Galle	IFS_SAL_736	KY888769	–	MH304354	genseq-4 COI
* Cheliceroides longipalpis *	China: Guizhou	–	KM033219.1	KM033179.1	–	–
*Echeclus* sp.	Malaysia: Selangor	–	KM033222.1	KM033182.1	–	–
Hasariusadansoni*	Sri Lanka: Kandy	IFS_SAL_268	KY888756	KY888749	KY888700	genseq-4 COI, 28S, 18S
Habrocestum cf. albimanum	South Africa: Western Cape Province	–	EU815611.1	EU815500.1	–	–
* Chinattus parvulus *	USA: North Carolina	–	EU815581.1	EU815464.1	–	–
Habrocestumkodigalaensis*	Sri Lanka: Kodigala	IFS_SAL_1000	MG910462	MG883391	MG883394	genseq-4 COI, 28S, 18S
*Habrocestum* sp.*	Sri Lanka: Loolecondera	IFS_SAL_827	KY888765	MG883390	MG883393	genseq-4 COI, 28S, 18S
Bristowiagandhii*	Sri Lanka: Badulla	IFS_SAL_357	KY888778	KY888738	KY888703	genseq-4 COI, 28S, 18S
* Idastrandia orientalis *	Malaysia: Sabah	–	EU815608.1	EU815496.1	EU815535.1	–
* Thiania latibola *	Malaysia: Pahang	–	KC615750.1	KC615569.1	–	–

**Table 2.** Phylogenetic data matrix scored for 17 taxa. The first state is ‘0’ followed by ‘1’ etc.; ‘?’ denotes missing data, ‘-‘ is inapplicable. * denotes outgroup taxa.

	0										1										2										3										4										5					
	1	2	3	4	5	6	7	8	9	0	1	2	3	4	5	6	7	8	9	0	1	2	3	4	5	6	7	8	9	0	1	2	3	4	5	6	7	8	9	0	1	2	3	4	5	6	7	8	9	0	1	2	3	4	5	6
* Bristowia gandhii *	0	0	0	0	-	0	0	0	0	0	2	1	1	0	1	0	1	2	0	0	-	0	0	0	1	1	1	0	0	1	4	0	-	0	1	0	1	0	0	0	0	1	1	0	0	0	-	–	1	1	0	0	0	0	0	0
* Hasarius adansoni *	0	0	0	0	-	0	0	0	0	0	0	0	0	0	1	0	0	2	0	0	-	0	0	1	1	0	1	0	0	0	4	0	-	0	0	-	-	-	0	0	0	1	0	-	1	0	-	–	0	0	0	0	4	0	0	1
* Habrocestum kodigalaensis *	0	0	0	0	-	0	0	0	0	0	0	0	0	0	0	0	0	2	0	0	-	0	0	1	1	0	0	0	0	0	4	0	-	0	1	3	1	1	0	0	0	1	0	-	1	0	-	–	0	0	0	0	5	0	1	1
* H. hantaneensis *	0	0	0	0	-	0	0	0	0	0	0	0	0	0	0	0	0	2	0	0	-	0	0	1	1	3	0	0	0	1	4	0	-	0	1	3	1	1	0	1	1	1	0	-	1	0	-	–	0	0	0	0	4	0	1	1
* Siler semiglaucus *	1	0	0	0	-	0	0	0	0	1	1	0	1	0	1	0	1	3	0	0	-	0	0	0	1	4	1	0	0	2	4	0	-	0	1	3	1	1	0	1	0	1	0	-	0	0	-	–	0	0	0	1	0	0	0	0
* Phintelloides jesudasi *	0	1	1	1	0	1	1	1	0	0	1	0	0	0	1	0	1	1	1	1	0	0	0	0	2	2	1	1	1	0	2	1	1	1	1	2	0	2	1	1	0	2	0	-	0	1	0	1	1	1	0	0	1	0	3	0
* P. brunne *	0	1	1	2	0	1	1	0	0	0	1	0	0	0	1	0	1	1	1	1	1	0	0	0	2	2	1	1	1	1	0	0	-	1	1	2	0	2	1	1	0	2	0	-	0	1	0	0	1	1	0	0	0	0	3	0
* P. orbisa *	0	?	?	?	?	?	1	0	0	0	?	?	?	?	?	0	1	0	0	?	?	?	0	0	?	?	?	?	?	?	?	?	?	?	?	?	?	?	?	?	?	?	0	-	0	1	1	1	2	3	2	0	2	1	3	0
* P. flavumi *	0	1	1	1	0	1	1	1	0	0	1	0	0	0	1	0	1	1	2	1	0	0	0	0	2	2	1	1	1	0	2	1	1	1	1	2	0	2	1	1	0	2	0	-	0	1	0	0	1	1	0	0	1	1	3	0
* P. alborea *	0	1	1	1	0	1	1	1	0	0	1	0	0	0	1	0	1	1	1	1	0	0	0	0	2	2	1	1	1	0	2	1	1	1	1	2	0	2	1	1	0	2	0	-	0	1	0	1	1	1	0	0	1	0	3	0
* P. flavoviri *	0	?	?	?	?	?	1	0	0	0	?	?	?	?	?	0	1	0	0	?	?	?	0	0	?	?	?	?	?	?	?	?	?	?	?	?	?	?	?	?	?	?	0	-	0	1	1	0	2	3	1	0	0	0	3	0
* Chrysilla lauta *	1	0	0	0	-	0	0	0	1	1	2	0	0	0	2	1	2	?	?	0	-	0	0	0	1	1	1	1	1	2	3	0	-	1	1	0	1	2	0	1	0	1	?	?	?	?	?	?	?	?	?	?	?	0	?	?
* C. volupe *	1	0	0	0	-	0	0	0	1	1	2	0	0	0	2	0	2	3	0	0	-	1	0	0	1	1	1	1	1	2	3	0	-	1	1	1	1	2	0	1	0	1	0	-	0	0	-	-	1	1	0	1	0	0	0	0
* Proszynskia diatreta *	0	0	1	3	1	1	0	0	0	0	2	0	0	0	1	0	1	1	3	1	2	0	0	0	0	5	1	0	0	2	0	0	-	1	1	4	1	0	0	0	0	2	1	0	0	0	-	-	1	1	0	1	3	0	2	0
* Phintella jaleeli *	0	0	0	0	-	0	0	0	0	0	2	0	0	1	1	0	1	2	0	0	-	0	0	0	0	0	1	0	0	2	0	0	-	1	1	2	0	2	0	0	1	2	1	0	0	0	-	-	0	1	0	0	0	0	0	0
* P. argentea *	2	0	0	3	2	0	0	0	0	0	1	0	0	0	1	0	1	3	0	0	-	0	1	0	0	0	1	0	0	2	1	1	0	1	1	3	1	0	0	0	0	0	1	1	0	0	-	-	1	1	0	2	0	0	0	0
* P. vittata *	2	0	0	3	2	0	0	0	0	0	1	0	0	0	1	0	1	4	0	0	-	0	1	0	0	0	1	0	0	2	0	1	0	1	1	3	1	0	0	0	0	0	1	0	0	0	-	-	1	2	0	2	1	0	0	0

### Morphology

Methodology and taxonomic descriptions are based on the format of Benjamin and Kanesharatnam (2016), Kanesharatnam and Benjamin (2016), and Benjamin (2004, 2010). Sampling was primarily done by beating vegetation up to a height of approximately 1.5 m. Specimens for the morphological study were preserved in 70% ethanol and identified using an Olympus SZX7 stereomicroscope. Female genitalia were excised and digested with Sigma Pancreatin lp 1750 enzyme complex, in a solution of sodium borate (Dingerkus and Uhler 1977). Male palps and epigynes were cleared and mounted with methyl salicyclate for further examination. Drawings of male palps, epigyne, and vulva were made with the aid of a drawing tube attached to an Olympus BX51 compound microscope. Either a Nikon D80 or a D7000 camera with a macro lens was used to take photographs of live spiders. Photographs of palps, epigynes, and intact spiders were taken with a Leica MC170 HD camera mounted on a Leica M205C stereomicroscope using Leica Application Suite software (Leica Microsystems Limited, Germany). Images were merged with Helicon Focus image stacking software (version 6, Helicon soft Ltd). Images were then edited with Adobe Photoshop CC and assembled using Adobe Illustrator CS6. A complete synonymy of the genera and species is given in World Spider Catalog (2018). All measurements are in millimetres. All specimens unless otherwise stated are deposited in the National Museum of Sri Lanka, Colombo.

### Morphological phylogenetic tree construction

The morphology of over 80 adult specimens was studied. Fifty-six potentially informative characters were identified and scored for 17 taxa (33 binary and 23 multistate). Twenty-three characters describe somatic morphology and 33 describe copulatory organ morphology. Mesquite (version 2.72; Maddison and Maddison 2009) was used to build and edit the data matrix. Parsimony analysis of the morphological data matrix was carried out using “traditional search” mode in TNT 1.1 (Goloboff et al. 2003; Goloboff, Farris and Nixon 2008). Under equal and implied weights, traditional searches were performed with following settings: 1000 random addition sequence replicates and tree bisection reconnection (tbr) swapping algorithm saving 10 trees per replication in TNT. The concavity constant (K) was set to values of 3–10, max trees was set to 100,000. Group support values and Bremer and Relative Bremer indices (Bremer 1988; Bremer 1994) were calculated using the ‘aquickie.run’ script in TNT. Ambiguous character optimisations were resolved to favour early gains of features with subsequent reversals (Farris optimisation or ACCTRAN). All multistate characters were treated as non-additive (unordered or Fitch minimum mutation model; Fitch 1971) as no transformation series could be inferred. Winclada version 1.00.08 (Nixon 2002) was used for mapping of characters and character states onto preferred parsimonious tree and strict consensus tree. The final matrix is submitted to the Dryad repository (https://doi.org/10.5061/dryad.b04s6t7).

### Laboratory protocols

Fresh specimens for DNA extraction were collected and preserved in 100% ethanol and/or RNA later® (Qiagen, Germany) and stored at -20 °C until extraction. If fresh material was unavailable, specimens preserved in 70% ethanol were used instead. Total genomic DNA was extracted from one or two legs or alternatively from the entire prosoma using the DNeasy Tissue Kit (Qiagen, Germany) as per manufacturer’s protocol. The targeted markers and primers are standard in arachnid systematics and are given in Table (3). Partial fragment of the mitochondrial protein-encoding gene cytochrome *c* oxidase subunit I (CO1 ~600 bp) and partial fragments of nuclear small and large subunit ribosomal RNA (18S ~1600 bp) and (28S ~800 bp) were amplified. The three overlapping fragments of 18S were amplified individually using three primer sets (Table 3). PCR amplification included a 2 min 94 °C initial denaturation and 35 iterations of 30 s at 94 °C, 30 s annealing step at 48 °C (18S and CO1) and 55 °C (28S), 30 s at 72 °C and one 10 min extension step at 72 °C (Maddison et al. 2014). Each PCR mix was prepared by the addition of 3 µl MgCl_2_, 1 µl dNTPs, 4 µl 5X colourless Gotaq^R^ flexi buffer, 1 µl forward and reverse primers (10 pmol/µl) and 0.4 µl Gotaq^R^ polymerase (Promega Corporation, 2800 Woods Hollow Road, Maddison, USA). Reaction volume was adjusted up to 20 µl by addition of 9.6 µl genomic DNA sample. For older specimens preserved in 70% ethanol, Qiagen Multiplex PCR kit (Qiagen, Germany) was used to enhance DNA yield. Reaction mixture per sample comprises 2.3 µl RNase free water, 10 µl QIAGEN Multiplex PCR Master Mix, 2 µl Q solution, 1.6 µl forward and reverse primers (10 pmol/µl) and 2.5 µl of DNA template. PCR was performed with an initial denaturation at 95 °C for 15 min then denaturation at 95 °C (30”), annealing temperature as mentioned above and extension at 72 °C for 1'30” for 35 cycles. Alternatively, Illustra^TM^ puRE Taq Ready-To-Go PCR beads (GE Healthcare, UK) was used for PCR amplification. As a rule, 1.6 µl of forward and reverse primers (10 pmol/µl) and 2.5 µl of the DNA template were added to the tubes supplied by the manufacturer. Molecular-grade RO water made up the remaining volume. PCRs were subsequently visualised in a 0.8% agarose gel. Sequencing of the purified PCR products were done at Macrogen (Seoul, South Korea).

**Table 3.** Details of genes, primer names, primer sequences, and sources of all primers used in this study.

Gene	Primer	References
CO1	CO1 1628 (F) 5’-ATAATGTAATTGTTACTGCTCA-3’	(Simon et al. 1994)
	C1-N-2191 (R) 5’-CCCGGTAAAATTAAAATATAAA-3’	(Dallas et al. 2005)
18S	18S 1F 5’-TACCTGGTTGATCCTGCCAGT-3’	(Giribet and Ribera 2000)
	18S 5R 5’-CTTGGCAAATGCTTTCGC-3’	(Giribet et al. 1996)
	18S 3F 5’-GTTCGATTCCGGAGAGGGA-3’	(Giribet et al. 1996)
	18S 7R 5’-GCATCACAGACCTGTTATTGC-3’	(Giribet et al. 1996)
	18S 4F 5’-CCAGCAGCCGCGCTAATTC-3’	(Giribet et al. 1996)
	18S 9R 5’-GATCCTTCCGCAGGTTCACCT-3’	(Giribet et al. 1996)
28S	28S “O” 5’-GAA ACT GCT CAA AGG TAA ACG G-3’	(Whiting et al. 1997)
	28S “C” 5’-GGT TCG ATT AGT CTT TCG CC-3’	(Whiting et al. 1997)

### Sequence alignment and editing

Chromatogram files were assembled and edited using Geneious 6.1.5 software package (Biomatters Limited; Kearse et al. 2012). Sequences were aligned in Geneious with options of automatically determine sequences’ direction, create alignment of consensus sequences only, gap open penalty 12, gap extension penalty 3, type of global alignment, refinement iterations 2. Edited sequences were queried by blasting against the NCBI BLAST database (http://blast.ncbi.nlm.nih.gov) as a check for possible contamination. The sequences obtained from NCBI were imported and aligned with our sequences in Geneious. The alignment of the non-coding 28S fragment was further refined manually in Mesquite. Finally, the fragments were assembled in to four separate multiple sequence alignments (*CO1*-single gene, *28S*-single gene, *18S*-single gene and concatenated *CO1*+*18S*+*28S*. The combined gene matrix was converted to tnt format using Sequence matrix 1.7.8 (Vaidya, Lohman, Meier 2011). All new sequences were submitted to GenBank; accession numbers are given in Table 1. The final concatenated alignment is submitted to the Dryad repository (https://doi.org/10.5061/dryad.b04s6t7).

### Phylogenetic tree construction

Both parsimony and likelihood methods were used as optimality criteria for the phylogenetic analyses. The three-gene combined data was analysed using ML and MP methods. The single gene matrices were analysed using ML methods only. RAxML–VI–HPC (randomised accelerated maximum likelihood for high performance computing v2.0.1) (Stamatakis 2006) as implemented in T-REX (Tree and reticulogram REConstruction) (Boc et al. 2012) was performed for maximum likelihood analysis using non-parametric bootstrapping in the program with the GTRGAMMAI model for all genes. MEGA 6.06 (Hall 2013) was used to determine the appropriate model by using the Akaike Information Criterion. For single gene matrices 100 search replicates and for the combined matrix 50 replicates were run. Clade support was assessed with 1000 bootstrap replicates (GTRGAMMAI model). Parsimony analysis was performed using TNT 1.1.

### Abbreviations

**Morphology**

**AEB** anterior epigynal border;

**AL** abdominal length;

**ALE** anterior lateral eyes;

**ALT** apical lobe of tegulum;

**AME** anterior median eyes;

**AT** atrium;

**AW** abdominal width;

**CD** copulatory ducts;

**CO** copulatory opening;

**CY** cymbium;

**DDC** duck-neck-shaped diverging curves;

**E** embolus;

**FD** fertilisation ducts;

**F** femur;

**HS** head-like structure;

**LP** lamellar process;

**MT** metatarsus;

**PA** patellar apophyses;

**PEB** posterior epigynal border;

**PL** prosoma length;

**PLE** posterior lateral eyes;

**PME** posterior median eyes;

**PT** patella;

**PW** prosoma width;

**PLT** proximal lobe of tegulum;

**RTA** retrolateral tibial apophysis;

**S** spermatheca;

**SC** scapum;

**SD** sperm duct;

**T** tegulum;

**TA** tarsus;

**TB** tibia;

**TEB** tegular bump;

**TL** total length;

**TR** trochanter;

**VR** vaginal roof.

**Additional abbreviations**

**FR** Forest reserve;

**ML** maximum likelihood;

**MP** maximum parsimony;

**SNR** Strict Nature Reserve.

## Description of characters and character states

### Somatic morphology

1. Body scale colouration: orange or black scales absent, 0; reddish orange, 1; black and golden yellow, 2.

In almost all *Chrysilla*, the body is covered with shiny metallic orange or black scales (Fig. 21A; Caleb and Mathai 2014: figs 15–18). Some *Phintella* species are also of a shiny metallic colouration (Figs 27A–F, 34A–F). However, all members of *Phintelloides* are lighter in colour (Figs 4A–H, 8A–F, 14A–F). *Silersemiglaucus* is covered with orange scales as in *Chrysilla*. However, they are not arranged in transverse rows on the prosoma: instead the pale metallic blue prosoma is decorated with broad, metallic, orange lateral bands (Kulkarni and Joseph 2015: fig. 1a, b; Prószyński and Deeleman-Reinhold 2010; Prószyński 1985).

2. White band on the clypeus of males: absent, 0; present, 1.

Clypeus of known males from *Phintelloides* is covered with a row of white tuft of depressed scales (Figs 4A, B, F, 8D, 14B). Caleb and Mathai (2014) termed this row of white tuft ‘short white moustache’ for *Phintelloidesjesudasi* comb. n. This might be an unambiguous synapomorphy of *Phintelloides*. Clypeus of most species of *Phintella* and *Proszynskiadiatreta* comb. n. are covered with glossy black or blackish brown scales, without any prominent stripes (Figs 23B, F, H, 27B, 34B). Clypeus of *Chrysilla* species are covered with metallic blue scales (Caleb and Mathai 2014: fig. 17).

3. Pale white / yellow anterior eye field in males: absent, 0; present, 1.

Half of the anterior portion of the eye field is covered with white dense scales in all known males of *Phintelloides*. (Figs 4A, E, F, 8A–D, 14A–C). This character is absent in males of *Chrysilla* and *Phintella*. Males of *Proszynskiadiatreta* possess discontinuous pale yellow blotches around eyes (Fig. 23E–G).

4. Diamond mark on prosoma: absent, 0; constricted, 1; stretched out posteriorly, 2; stretched out laterally, 3.

5. Colour of diamond mark: white, 0; greyish white, 1; metallic colouration, 2.

A conspicuous transverse, white diamond-shaped mark, just behind ocular field is diagnostic for males of *Phintelloides* (Figs 4E, F, 8A–C, 14A–C). *Proszynskiadiatreta* has greyish white diamond-shaped blotch with vague margins (Fig. 23E, G). *Phintellavittata* (Koch, 1846) has a laterally stretched out metallic blue and *P.argentea* sp. n. has a silvery diamond-shaped mark posterior to the eye field in both sexes (Figs 27A, C–F, 34A–F).

6. Lateral white belts of the male prosoma: absent, 0; present, 1.

In all known males of *Phintelloides* the lateral margins of prosoma is adorned with a broad contrasting white belts covering one third height of prosoma (Figs 4A, B, E, F, 8A–C, 14A, C). A similar discontinuous, somewhat less prominent band is present in *Proszynskiadiatreta* (Fig. 23E–G). In *Phintellavittata*, the lateral prosoma is decorated with blue metallic bands and in *P.argentea* with silvery blotches instead of silvery belts (Figs 27A, E, F, 34E, F); both scored as absent. Males of *Hasariusadansoni* are covered with a broad, white belt around the eye field that extends continuously from the vicinity of ALE to the middle of prosoma, just behind PLE in a semicircle (Kim and Kim 1996: fig. 1; Peng et al. 1993; Prószyński 2003). However, this belt is unlike that of *Phintelloides*.

7. Prominent dark blotches around ale and PLE in females: absent, 0; present, 1.

8. Prominent dark blotches behind PLE in females: absent, 0; present, 1.

All known females of *Phintelloides* possess black blotches on the prosoma (Figs 4D, G, H, 12A, E, 14D–F). In addition, *Phintelloidesjesudasi*, *P.flavumi* sp. n., and *P.alborea* sp. n. have black patches on the posterior region of prosoma behind PLE (Figs 4D, G, H, 14D–F). These patches are absent in all other studied genera.

9. Prosoma with horizontal iridescent blue and reddish orange bands in males: absent, 0; present, 1.

This character is present in both *Chrysillavolupe* (Karsch, 1879) and *C.lauta* Thorell, 1887 (Figs 19A, 21A, Caleb and Mathai 2014: fig. 15). Although, *C.deelemani* Prószyński & Deeleman-Reinhold, 2010 is similar to *C.volupe* and *C.lauta* in palpal morphology, it lacks this banding pattern (Prószyński and Deeleman-Reinhold 2010: fig. 31). All other known *Chrysilla* species also lack these bands. The metallic blue prosoma of *S.semiglaucus* is furnished with reddish orange lateral belts. However, they are not arranged in rows as described above (Kulkarni and Joseph 2015: fig. 1a, b; Prószyński 1985).

10. Metallic blue edges of prosoma: absent, 0; present, 1.

Lateral edges of prosoma in *C.volupe*, *C.lauta*, and *S.semiglaucus* are covered with metallic blue scales in a rather broad stripe (Fig. 21A; Caleb and Mathai 2014: figs 15, 18; Kulkarni and Joseph 2015: fig. 1a, b). However, *C.volupe* and *C.lauta* have additional metallic blue stripe just below PLE on the laterals sides of the prosoma (Caleb and Mathai 2014: fig. 18). This character further highlights the close resemblance of both species.

11. First pair of legs in males, relative thickness: normal, 0; slightly thickened, 1; thickened, 2.

12. First pair of legs in males, relative length: normal, 0; elongated, 1;

These two characters described the relative thickness and length of leg I. *Bristowiagandhii* Kanesharatnam and Benjamin, 2016 is easily distinguishable by their elongated coxa, trochanter, and patella of leg I. They are strongly modified in males than in females (Kanesharatnam and Benjamin, 2016: fig. 3a). First legs of females are comparably less strong and not enlarged in all other taxa considered in this character matrix.

13. First pair of legs furnished with a ventral fringe of black bristles: absent, 0; present, 1.

In *B.gandhii* both the male and female, patella I and tibia I are furnished with a ventral fringe of black bristles (Kanesharatnam and Benjamin, 2016: fig. 3a, b). In *S.semiglaucus*, only males possess black bristles (Prószyński 1985); scored as present.

14. Tibia I with black blotches in males: absent, 0; present, 1.

Males of *Phintellajaleeli* sp. n. have prominent large and black blotches at the distal end of dorsal side of tibia I (Fig. 23A, B). Absent in females, which have black longitudinal lateral stripes on femur, patella, and tibia of all legs (Fig. 23C, D).

15. Shape of the abdomen in males: stout and tapered, 0; elliptical, 1; more elongated, 2.

This character differentiates males of *Chrysilla* from *Phintella*. *Chrysillavolupe* and *C.lauta* are characterised by a rather more elongated abdomen (Figs 19A, B, 21A, B). However, females of both genera have no obvious differences: all females have a stout and somewhat tapered abdomen (Figs 22A, B, 27C, D, 29A, B, E, F, 34C).

16. Abdominal scutum: absent, 0; present, 1.

In *C.lauta*, the abdomen is fully covered with a metallic, blackish blue scutum (Fig. 19A, B); scored as absent in *C.volupe* and this apomorphic character differentiates *C.lauta* from *C.volupe*.

17. Relative abdominal length: shorter than one time width, 0; length less than twice of width, 1; much longer than twice of width, 2.

This character describes the longer and narrower abdomen of *Chrysilla* (Figs 19A, B, 21A, B). Abdominal length is scored in relation to its width. In *H.kodigalaensis*Kanesharatnam and Benjamin 2016 and *H.hantaneensis*Kanesharatnam and Benjamin 2016, abdomen is much smaller than their prosoma (Kanesharatnam and Benjamin 2016: figs 5a, b, 7a–c); scored as 0.

18. Abdominal dorsal pattern in females: none, 0; stripes or streaks, 1; non-metallic markings, 2; metallic markings, 3; transverse metallic banding, 4.

This character is scored based on specimens in life. Males and females of *Phintelloidesjesudasi* comb. n., *P.flavumi* sp. n. and *P.alborea* sp. n. are sexually dimorphic; they differ in colour and organisation of the stripe pattern (Figs 4A–H, 8A–D, 14A–F). Females of *P.orbisa* sp. n. and *P.flavoviri* sp. n. are uniformly greenish yellow coloured and lack any markings on the dorsum of abdomen (Fig. 8E, F).

19. Stripe pattern of abdomen in females: absent, 0; narrow, not converging, 1; broad, converging, 2; broad, not converging, 3.

Longitudinal blackish green stripes of *Phintelloidesjesudasi* and *P.alborea* are narrow and almost parallel to each other. They do not converge near the spinnerets (Fig. 4D, G, H). In *P.flavumi* sp. n., stripes are comparably broader and converge at the end (Fig. 14D–F).

20. Longitudinal banding pattern of abdomen in males: absent, 0; present, 1.

All males of *Phintelloides* including *P.versicolor* comb. n. have a characteristic arrangement of longitudinal stripes on abdomen (Figs 4A, E–F, 8A–C, 14A–C). This may be diagnostic for *Phintelloides*. However, females of *P.versicolor* lack banding; instead, abdomen is covered with irregular brownish black blotches.

21. Arrangement of mid-dorsal and lateral bands in males: greyish black median band surrounded by pale yellow lateral bands, 0; brownish black median band bordered by white lateral bands, 1; pale yellow median band bordered with two black bands, 2.

This character describes the arrangement of bands in males of *Phintelloides*.

22. “M”-shaped metallic orange abdominal band in males: absent, 0; present, 1.

This apomorphic character differentiates males of *Chrysillavolupe* (Fig. 21A; Caleb and Mathai 2014: fig. 15) from those of *C.lauta*.

23. Anterior abdomen with a transverse silvery band: absent, 0; present, 1.

In some species of *Phintella*, anterior portion of the abdomen is covered with a metallic coloured transverse silvery band. Both *P.vittata* and *P.argentea* have a bluish silvery anterior abdomen (Figs 27A–F, 34A–F). Male of *Hasariusadansoni* also possess a semicircular non-metallic white band on the anterior abdomen (Kim and Kim 1996: fig. 1); scored as absent.

### Copulatory organ morphology

**Male palp**

24. White-edged palp: absent, 0; present, 1.

Hasariines are often decorated with white, conspicuous patches at the edges of their palps (Maddison 2015). In *Habrocestumkodigalaensis* and *H.hantaneensis*, large, round spot of white or pale yellow patches are visible dorsally (Kanesharatnam and Benjamin 2016: fig. 5c–d). In *Hasariusadansoni*, dorsal edges of palps are covered with dense white patches.

25. Size of the embolus: small, much smaller than one fourth length of the cymbium, 0; medium, less than half length of the cymbium, 1; long, more than half length of the cymbium, 2.

26. Embolus structure: stout, 0; slender, 1; filiform 2; funnel-shaped, 3; crescent-shaped, 4; more/less straight, 5

A shorter, stouter embolus set at a slightly oblique angle toward the retrolateral position on the apical portion of tegulum is diagnostic for *Phintella* (Figs 28D, E, 30B, C, 31D, E, 33A, B, 35D, E, 36A, B). *Chrysilla* has a comparably slender, medium sized and retrolaterally curved embolus (Figs 19C–E, 20A–D, 21C–E). *Phintelloides* is characterised by a thinner and longer embolus (Figs 5D, E, 7A, B, 9D, E, 11A, B, 15D, E, 16A, B, 17D, E, 18A, B).

27. Origin of the embolus: embolic base below the apical tegular ridge 0; fixed to the apical tegular ridge, 1.

In *Habrocestumkodigalaensis* and *H.hantaneensis*, the embolus originates internally from below the apical tegular ridge (Kanesharatnam and Benjamin 2016: figs 5c, d, 6a, 7d, 8a). In all other taxa considered here, the base of the embolus is visible in the ventral view of the bulb. It is fixed embolus to the apical ridge.

28. Size of the cymbium: short, 0; long, 1.

Cymbial length is scored in relation to its maximum width (near broader proximal region). Short and long are coded as less and more than two times of its width respectively. In *Phintelloides*, *P.brunne* is characterised with comparably shorter and broader cymbium than its congeners, although it is coded as long. (Figs 9C–E, 11A, B). In *Chrysilla* and *Phintelloides*, cymbium is scored as long. All *Phintella* species used in the present context and *Proszynskiadiatreta* possess short cymbium.

29. Distal half of cymbium: blunted/tapered 0; elongated 1.

In *Phintella* and *Proszynskiadiatreta*, the distal cymbium is characterised with a tapering end (Figs 28C–E, 30A–C, 31C–E, 33A, B, 35D, E, 36A, B). *Phintelloides* and *Chrysilla* possess an elongated cymbium with much narrower distal end (Figs 5C–E, 7A, B, 9C–E, 11A, B, 15C–E, 16A, B, 17C–E, 18A, B, 19C–E, 20A–D, 21C–E).

30. Apical portion of tegulum in relation to its distal end: at same level, 0; slightly elongated, 1; more elongated, 2.

In *Phintella* and *Chrysilla*, ALT prolongs beyond the boundary of the distal tegulum (Figs 19D–E, 20A–D, 21D, E, 28D–E, 30B, C, 31D, E, 33A, B, 35D, E, 36A). All known males of *Phintelloides* are characterised with nearly the same level of apical and distal portion of tegulum. This character distinguishes them from *Phintella* and *Chrysilla* (Figs 5D, E, 7A, B, 9D, 11A, 15D, 16A, 17D, 18A).

31. Shape of apical lobe of tegulum: triangular, 0; elongated-triangular, 1; broad and irregular, 2; elongated-semicircular 3; semicircular, 4.

*Phintellaargentea* is distinguished from its closest relative *P.vittata* by comparably more elongated triangular apical tegulum (Figs 28D, E, 30B, C). The presence of an elongated-semicircular apical tegulum is synapomorphic for *Chrysillavolupe* and *C.lauta* (Figs 19D, 20A, C, 21D).

32. Lamellar process (LP): absent, 0; present, 1.

Refers to any outgrowth from the apical lobe of the tegulum. It is absent in *Phintelloidesbrunne* (Figs 9D, 11A) and partially developed in *P.alborea* and *P.jesudasi* (Figs 5D, 7A, 17D, 18A). It is well developed in *P.flavumi* and most species of *Phintella* (Figs 15D, 16A, 28D, 30B, 35D, 36A). The apical tegulum is smooth without any projection in *Proszynskiadiatreta*, *Phintellajaleeli* (Figs 24D, 25A, 31D, 33A) and all known *Chrysilla* species (Figs 19D, 20A, C, 21D). Peng et al. (1993) described this similar structure for *Phintella* as “flaky outgrowth”.

33. Shape of the LP: triangular, 0; semicircular, 1.

*Phintelloidesflavumi* has a well-developed semicircular LP immediately lying below embolus (Figs 15D, 16A). *Phintellavittata* and *P.argentea* possess a well-developed triangular LP (Figs 28D, 30B, 35D, 36A).

34. Tegular bump: absent, 0; present, 1.

Most of the chrysillines have a bump on tegulum ca. 90°clockwise from the base of the embolus of the left palp (Maddison 2015). All known males of *Chrysilla*, *Phintelloides*, *Phintella* and *Proszynskia* have a triangular-shaped bump at the retrolateral portion of the tegulum (Figs 5C, E, 7A–B, 9E, 15C, 17C, 28C, 30B, C, 31C–E, 33A, B, 35C, D, 36A, B).

35. Proximal lobe of tegulum: absent, 0; present, 1.

36. Shape of the tegular proximal lobe: rounded, 0; rectangular, 1; flap-like , 2; irregular elongated, 3; elongated semicircular, 4.

37. Size of the proximal lobe: small, 0; large, 1.

38. Position of the proximal lobe: prolateral, 0; retrolateral, 1; posterior, 2.

The tegulum is characterised with a proximal lobe in all ingroup and outgroup taxa except for *Hasariusadansoni* (Kim and Kim 1996: figs 2–3). The proximal lobe of all known males of *Phintelloides* (Figs 5C–E, 7A, 9C–E, 11A, 15C–D, 16A, 17D–E, 18A) and *Phintellajaleeli* (Figs 31D–E, 33A–B) is modified as a small flap-like structure at the posterior tegulum. In *P.vittata* and *P.argentea*, PLT is well-developed postero-prolaterally prolonging nearly half of the palpal tibia (Figs 28D, E, 30A–C, 35D, E, 36A).

39. Triangular-shaped bulbus: absent, 0; present, 1.

All known males of *Phintelloides* have triangular bulbus excluding apical and posterior lobe and it is slightly oblique to apical lobe (Figs 5D, 7A, 9D, 11A, 15D, 16A, 17D, 18A). This is an important synapomorphy that differentiates *Phintelloides* from closely related genera *Phintella*, *Proszynskia*, as well as other chrysillines.

40. RTA nearly half of the tegulum: absent, 0; present, 1.

Present in *Phintelloides*. Males of *Phintelloides* have a comparably straight RTA with a slightly bent tip (Figs 5D–E, 7A–B, 9D–E, 11A–B, 15D–E, 16A–B, 17D–E, 18A–B). *Chrysillavolupe* and *C.lauta* possess robust RTA with very broad basal portion (Figs 19D, E, 20A–D, 21D, E). *Habrocestumhantaneensis* (Kanesharatnam and Benjamin 2016: 5c–d, 6a–b) and *S.semiglaucus* possess very long RTA. Generally chrysillines have single apophyses on the palpal tibia; however, some species of *Phintella* including *P.vittata* possess double RTA of unequal size. One is blunted and small. The other is with a pointed tip (Figs 35D, E, 36A, B).

41. RTA with minute teeth at ventral margin: absent, 0; present, 1.

In *Phintellajaleeli* and *Habrocestumhantaneensis*, the inner margin of RTA is covered with minute teeth (Figs 31D–E, 33A–C, Kanesharatnam and Benjamin 2016: figs 5c, d, 6a, b). Absent in other species of *Phintella*. The presence of teeth on the RTA may function as a tight lock with the epigynum (Eberhard and Huber 2009).

42. Shape of RTA: bent backwards, dorsally, 0; bent forward, ventrally, 1; straight with slightly curved tip, 2.

### Epigynum

43. Anterior epigynal border: absent, 0; present, 1.

44. Sclerotisation of anterior epigynal border: poor, 0; high, 1.

In all species of *Phintella* described in our character matrix, the anterior portion of epigynum is covered with different levels of sclerotisation (Figs 29C, D, G, H, 32C, D, 36C, D).

45. Transverse and membranous white “window”: absent, 0; present, 1.

Within the outgroup taxa (except for *B.gandhii*), all hasariines (*Hasariusadansoni*, *Habrocestumhantaneensis*, and *H.kodigalaensis*) possess a large window-like structure into which the CO may open. However, the exact position of CO is uncertain (Kanesharatnam and Benjamin 2016: figs 5e, f, 6c, d, 7f, 8c, d).

46. DDC of CD at anterior margin: absent, 0; present, 1.

DDC as designated here is the curved anterior section of CD. This part is curved in the shape of a duck’s neck. This character is diagnostic for *Phintelloides*. It is not known to occur in any other salticidae genera. In *Phintelloidesjesudasi*, *P.orbisa*, and *P.alborea*, CO is on the outer side of the curve (Figs 6C, D, G, H, 7C, D, 12G, H, 13A, B, 18C, D). In other species, the position of CO is unclear; it might be covered by the much broader DDC (Figs 10C, D, G, H, 11C, D, 12C, D, 13C, D, 16C, D).

47. Width of DDC in relation to CD: broader, 0; much broader, 1.

Here, size of the DDC is described in relation to the width of the middle portion of the CD. DDC less than or more than three times broader than the width of CD are coded as broader and much broader respectively.

48. Space between DDC and CD: absent, 0; present, 1.

This character describes the space between DDC and CD in *Phintelloidesjesudasi* and *P.alborea*. This space is comparably larger than one time width of CD in *P.jesudasi* (Figs 6C, D, G, H, 7C, D, 12G, H, 13A, B, 18C, D).

49. Total length of the CD, including DDC: short, 0; moderately long, 1; very long, 2.

Here, the total length of CD is scored in relation to the diameter of spermathecae. Duct much shorter and longer than one diameter of spermathecae are scored as short and moderately long respectively. CD longer than four or more times receptacle diameter is coded as very long. Other hasariines possess much shorter CD, except for *B.gandhii* (Kanesharatnam and Benjamin 2016: figs 5e, f, 6c, d, 8c, d).

50. Progression of CD: not curved, 0; curved, 1; “V”-shaped, 2; twisted, 3.

51. Number of coils of CD: no coiling, 0; one, 1; three, 2.

Based on the highly twisted structure of CD in *Phintelloidesorbisa* (Figs 12G, H, 13A, B), we can predict that the embolus of unknown male could be much longer than its congeners. Same could be said for unknown male of *P.flavoviri* (Figs 12C, D, 13C, D).

52. Scapum: present, 0; scapum, partially developed, 1; scapum, well-developed, 2.

Epigynal scape is a tongue-like structure at the posterior margin. In *Phintelloides*, the posterior margin is characterised by a rather broad plate-like structure termed basal plate (Figs 6C, D, G, H, 7C, D, 10C, D, G, H, 11C, D, 12C, D, G, H, 13A–D, 16C, D, 18C, D). *Phintellavittata* and *P.argentea* possess a well-developed scape, which is a sclerotised and tongue-shaped structure protruding beyond the epigastric furrow (Figs 29C, D, G, H, 30D, E, 36C, D).

53. Shape of spermathecae: rounded, 0; oval, 1; reniform, 2; pyriform, 3; irregular, 4; triangular, 5.

54. Head of receptacles (spermathecal head): absent, 0; present, 1.

*Phintelloides*, *P.orbisa* and *P.flavumi* are characterised by a well-developed spermathecal head at the anterior apical wall of the spermathecae (Figs 10C, D, 12G, H, 13A, B, 16C, D).

55. The origin of FD from the wall of spermathecae: anterior, 0; anterolateral, 1; mid-dorsal, 2; apical, 3.

In *Phintelloides*, FD originates from the narrowed apex of the receptacles (Figs 6D, H, 9D, 10D, H, 11D, 12D, H, 13D, 16D, 18D). In *Phintella* and *Chrysilla* species, FD is from the anterior wall of origin. *Proszynskiadiatreta* has an unusual funnel-like structure on the mid wall of receptacles from which FD originates (Figs 25E, 26D).

56. Vaginal roof, absent, 0; present, 1.

The small sclerotised pocket-like structure at posterior margin near the epigastric furrow is termed as vaginal roof. The function of this structure is unknown. *Habrocestumhantaneensis* has a wide vaginal roof with a shallow notch (VR in Kanesharatnam and Benjamin 2016: figs 5e, f, 6c, d) whereas, *Hasariusadansoni* and *Habrocestumkodigalaensis* have a comparably small vaginal roof with a deep notch (Davies and Żabka 1989; Kanesharatnam and Benjamin 2016: 7f, 8c, d).

## Results

### Molecular phylogeny

**Figure 1.**
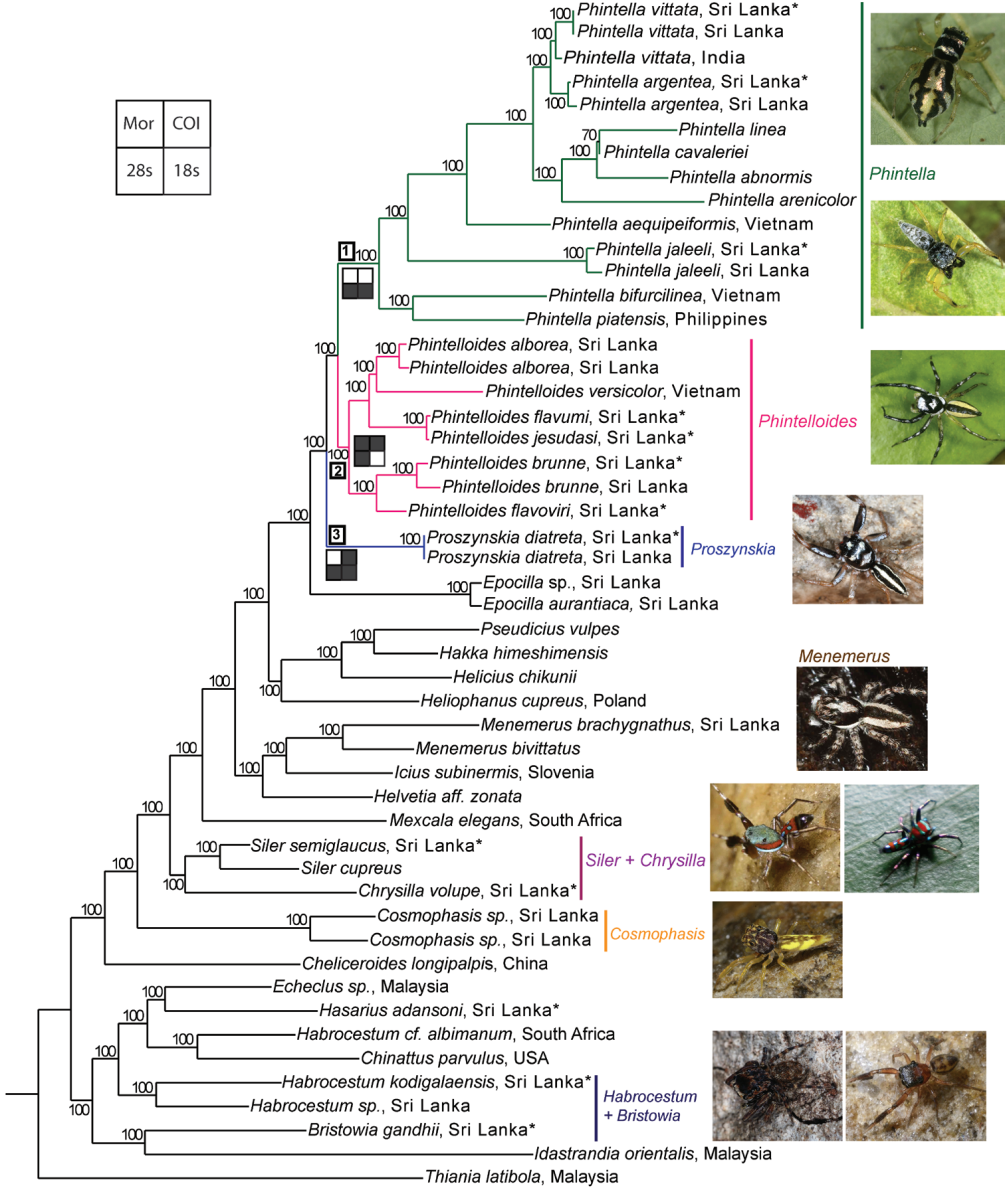
The single most likely tree obtained by ML analysis of the combined molecular data in RAxML–VI–HPC. The numbers at the nodes represent bootstrap values (only values 60 and above are given). Nodes that are unsupported have been collapsed. Collection country is given if available. Key: “Navajo rugs” indicate presence (black) or absence (white) of a given node in the tree specified in the legend.* denotes taxa included in the morphological analysis; resulting tree is given in Fig. (3).

**Figure 2.**
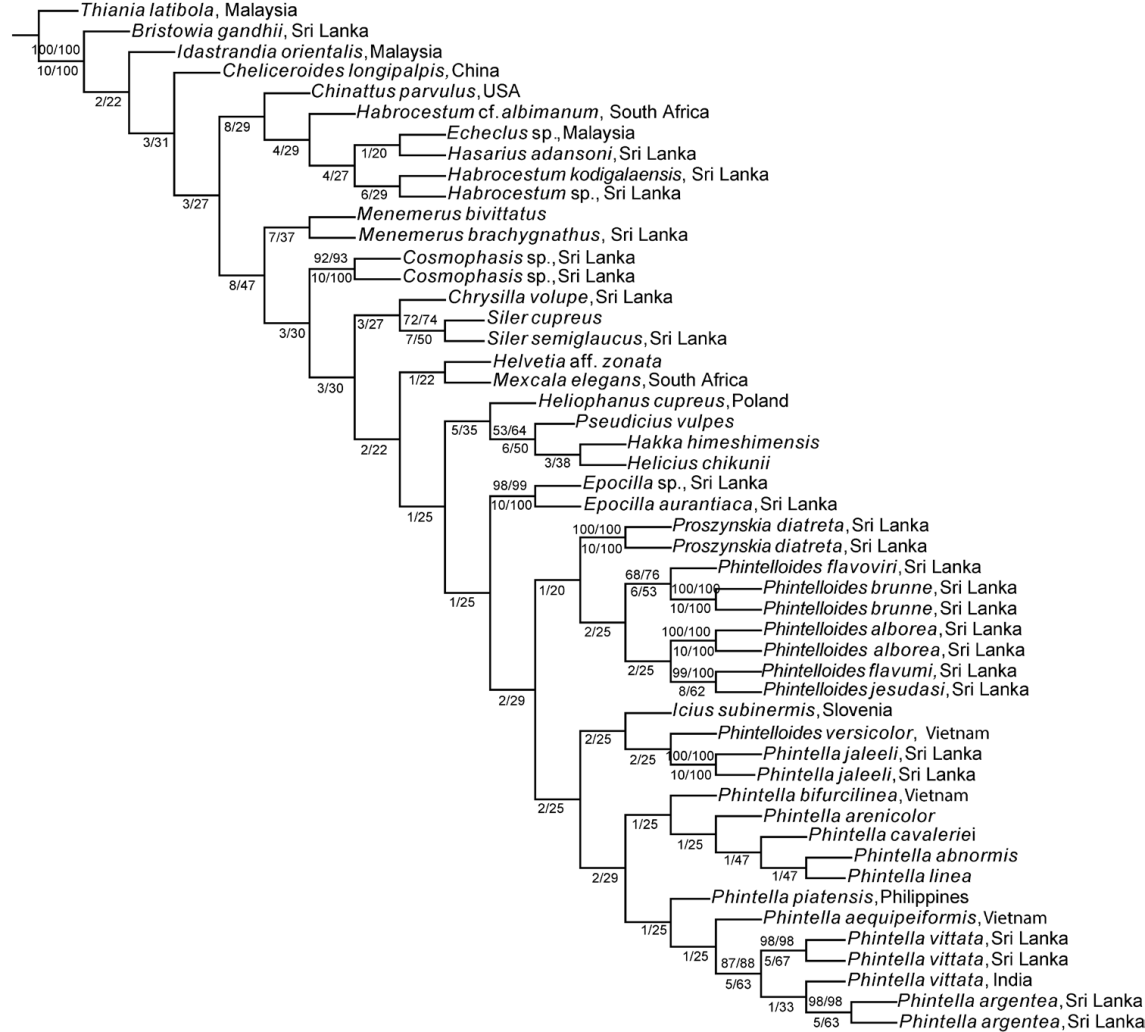
The single most parsimonious tree obtained by analysis of the combined molecular data in TNT. The numbers at the nodes represent bootstrap values (only values above 60 are given). Nodes that are unsupported have been collapsed. Collection country is given if available.

The concatenation of aligned sequences of the three genes resulted in a 50 taxa 2876 bp long matrix. The COI gene resulted in a 46 taxa matrix of 552 bp. The 28s resulted in a 38 taxa matrix of 778 bp and 18s gene resulted in a 32 taxa matrix of 1715 bp. The ML phylogenetic analysis of the three genes combined data retrieved a single tree (Fig. 1). This tree recovers three well-supported clades. Clade 1 contains all species of *Phintella* included in the study. Clades 2 and 3 contain species provisionally identified by us as *Chrysilla* or *Phintella*. These two clades are described here as new genera *Phintelloides* and *Proszynskia*. The evidence for this decision is summarised and discussed below in the taxonomic section. The resulting tree of MP analysis of the same matrix is given in Fig. 2.

All resulting single gene ML phylogenies were examined to assess their differences to the preferred tree of Fig. 1. In the COI phylogeny clades 2 and 3 are recovered (Suppl. material 1: Fig. S1). Clade 1 is not recovered with *Phintellabifurcilinea* (Bösenberg & Strand, 1906) and *P.jaleeli* grouping out separate from all other *Phintella*. However, these groupings receive low support. The 28s phylogeny recovers all three clades and they are well supported (Suppl. material 2: Fig. S2). The 18s phylogeny recover well-supported clades 1 and 3 (Suppl. material 3: Fig. S3). However, clade 2 is not recovered. Thus, we chose to focus on the ML tree inferred from the 3-gene combined data set and base our taxonomic decisions on it.

*Phintelloides* is recovered as a separate lineage in the ML tree inferred from the three genes, COI and 28s genes with high support (Fig. 1; Suppl. material 1, 2: Figs S1, S2). However, this lineage is not recovered in the MP tree inferred from the 3-gene combined data and the 18s tree (Fig. 2; Suppl. material 3: Fig. S3). In MP tree inferred from the three genes *Phintelloidesversicolor* (part of clade two in the ML analysis) groups outside of all other *Phintelloides*.

**Figure 3.**
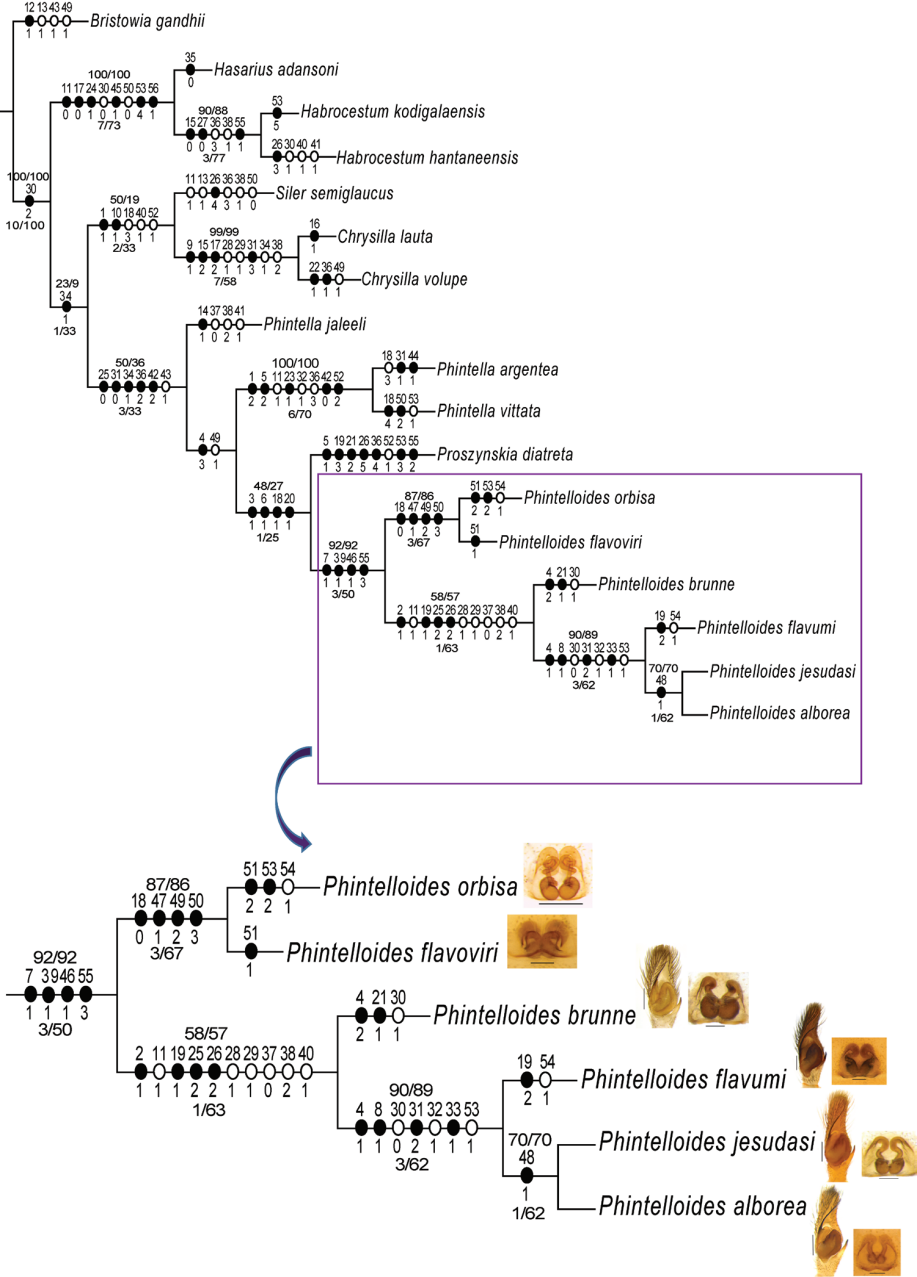
The single MP tree obtained by parsimony analysis in TNT under implied weights of the 56 morphological characters given in Table 2. Unambiguous character state changes are mapped using Farris optimisation. Characters are denoted by the numbers above the circles and character state changes by numbers below the circles. The values above the lines represent sympatric resampling frequencies/sympatric resampling frequency differences, while the values below the line represent Bremer support/relative Bremer support.

*Proszynskia* is recovered as a separate lineage in all our analyses with high support (Figs 1–3; Suppl. material 1–3: Figs S1–S3). However, its placement within trees differs: in the COI tree it is sister to *Phintelloides* and a large group of other chrysillins including *Phintella*. In the 28s tree it is sister to *Phintella*; in the 18s tree *Proszynskia* + *Epocilla* is sister to *Phintelloides* + *Phintella*.

### Morphological phylogeny

Distribution of characters and their character states is given in Table 2. Parsimony analysis under equal weights of this matrix resulted in two most parsimonious trees (best score of 125). The same data matrix reanalysed under implied weights, resulted in a single most parsimonious tree (best score of 4.95) with fully resolved branches (L = 110, CI = 0.78, RI = 0.84). This tree does not recover the three lineages recovered in the molecular analysis (Figs 1, 2). However, a single lineage containing species provisionally identified by us as *Chrysilla* or *Phintella* was recovered with high support, which is identical in species composition to the lineage recovered in all molecular trees and described as *Phintelloides* below.

The monophyly of *Phintelloides* is supported by the following putative synapomorphies: triangular-shaped bulbus with slightly oblique orientation in relation to its apical lobe (39–1), long embolus (25–2), white band on the clypeus (2–1), pale white band on the anterior eye field (3–1), white diamond-shaped marking on the prosoma (4–1, 5–0), lateral white belts (6–1), black median band bordered by two lateral bands on the abdomen in males (21–0,1), conspicuous black blotches on the prosoma of females (7–1), duck-neck-shaped diverging curves at anterior margin of epigynum (46–1), apical origin of FD (55–3), out of which the presence of triangular bulbus and DDC are unambiguous synapomorphies delimiting all known species of *Phintelloides*. Within the *Phintelloides* clade, *P.orbisa* and *P.flavoviri* are sister species supported by unambiguous synapomorphies: devoid of any markings or stripes of abdomen (18–0), presence of much broader DDC (47–1), very long and twisted CD with coils (49–2, 50–3). Further, *P.brunne* is sister to *P.flavumi* + (*P.jesudasi* +*P.alborea*).

*Proszynskiadiatreta* is sister to all *Phintelloides*. It shares the following unambiguous synapomorphies with *Phintelloides*: presence of pale white anterior eye field and lateral white belts in males (3–1, 6–1), dorsum of abdomen with stripe pattern in females (18–1), arrangement of stripe pattern of abdominal dorsum in males (21–2). A set of characters can differentiate *Proszynskia* from other chrysillines; large body size, origin of FD in funnel-like structures, pale yellow median abdominal band bordered with two black bands (Figs 23E–G), more/less straight embolus (Figs 24D, 25A). A monophyletic *Phintella* is not recovered. However, *Phintelloides*, *Phintella*, and *Proszynskia* are more closely related to each other than to *Siler* and *Chrysilla*, included in this morphological analysis.

### Taxonomy

The results presented below are based on the tree generated using the concatenated molecular markers and ML optimality criteria (Fig. 1).

### Family Salticidae Blackwall, 1841

#### Subfamily Salticinae Blackwall, 1841

##### Tribe Chrysillini Simon, 1901

###### Genus Phintelloides gen. n.

Taxon classificationAnimaliaAraneaeSalticidae

http://zoobank.org/DC99FB77-D3F9-4330-AFE7-304075FACB80

####### Type species.

*Chrysillajesudasi* Caleb & Mathai, 2014.

####### Etymology.

Combination of “Phintell” taken from *Phintella* and “oides” meaning “having the form of”. This name also refers to the closer relationship of *Phintelloides* to *Phintella* than to other chrysillines. Gender masculine.

####### Monophyly and phylogenetic placement.

**Figure 4.**
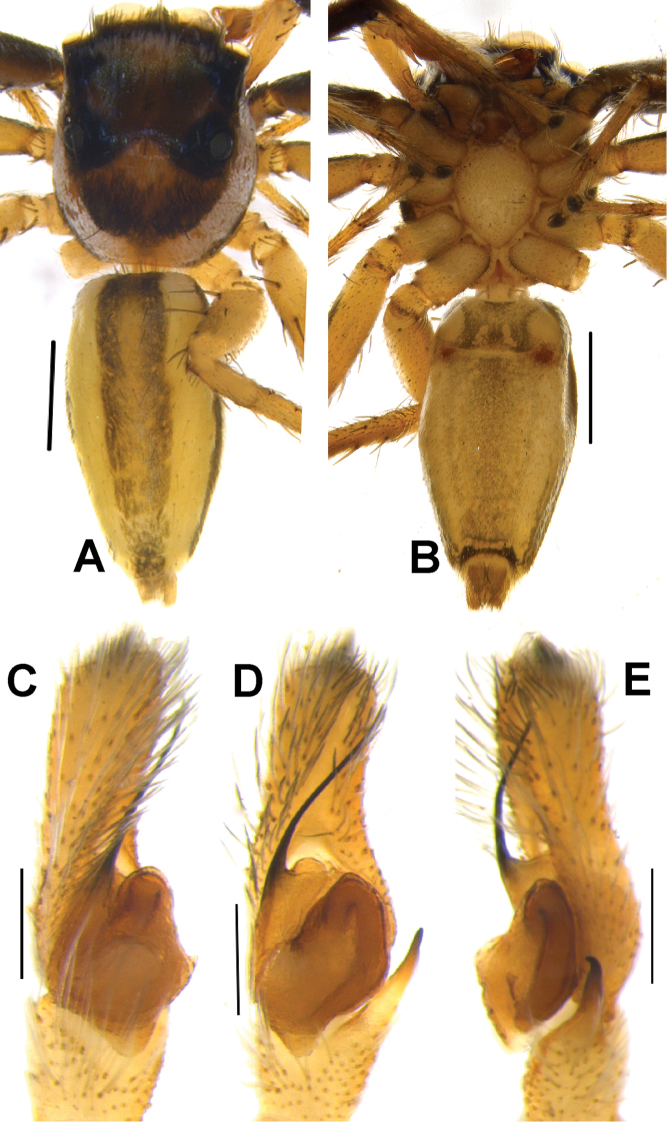
*Phintelloidesalborea* (**A–D**) from Dambulla, National Institute of Fundamental Studies, Arboretum **A, B** Male in life **C, D** Female in life and *Phintelloidesjesudasi* (E–H) from Pilikuttuwa **E, F** Male in life **G, H** Female in life.

**Figure 5.**
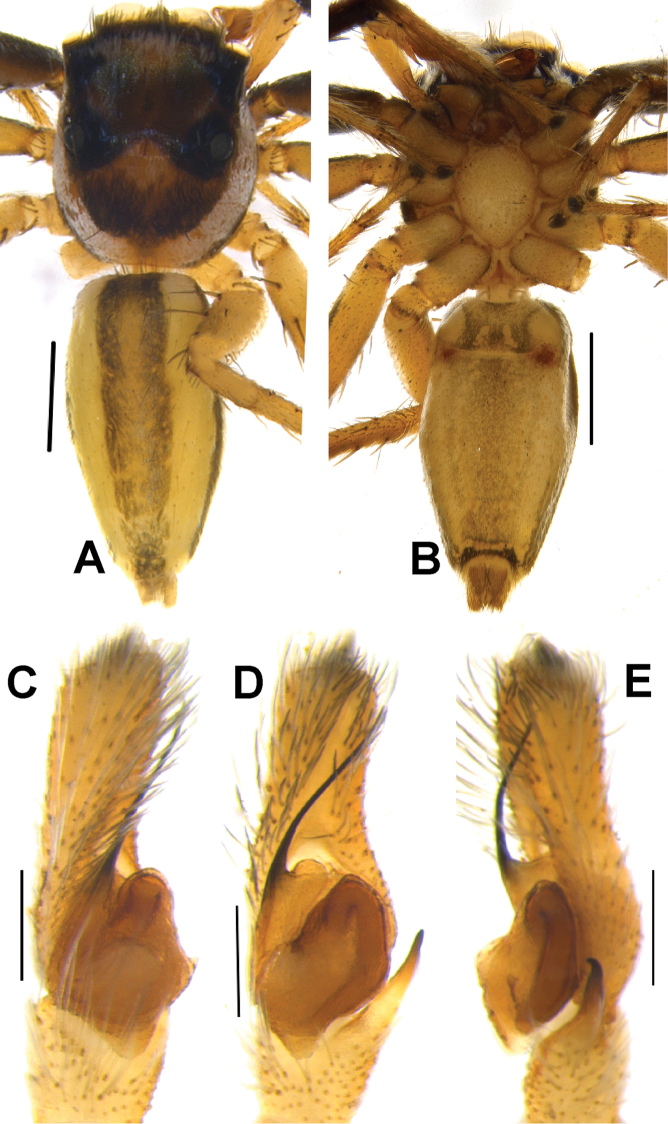
*Phintelloidesalborea* (**A, B**) Male habitus **A** dorsal view **B** ventral view **C–E** Male palp **C** prolateral view **D** ventral view **E** retrolateral view. Scale bars 1 mm (**A, B**), 0.2 mm (**C–E**).

**Figure 6.**
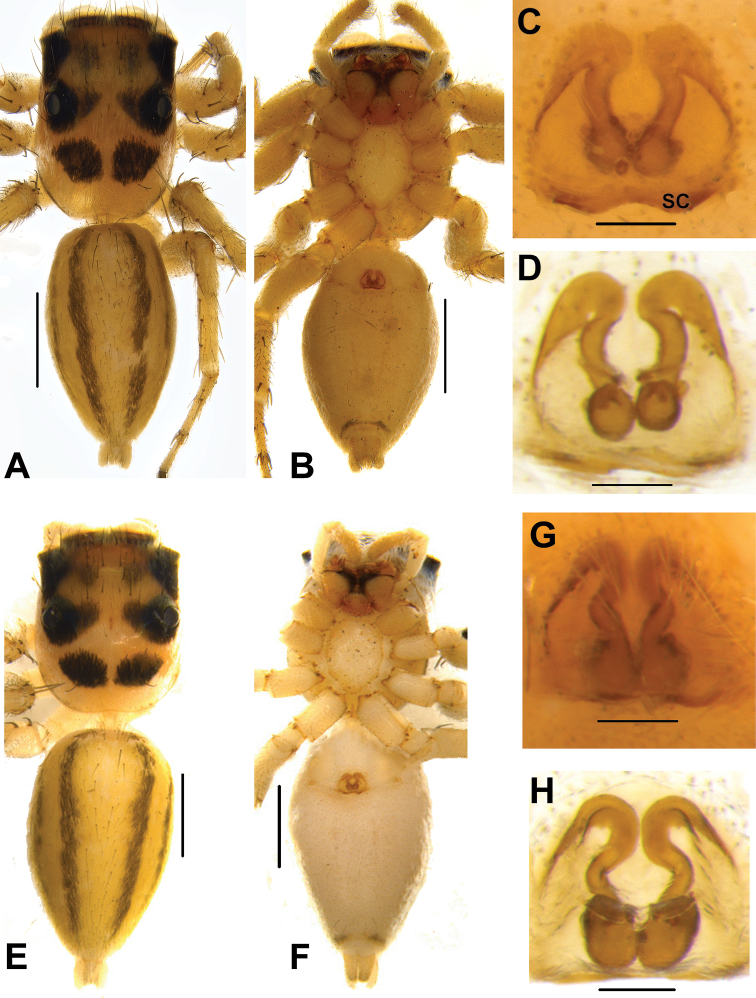
*Phintelloidesalborea* (**A–D**) **A–B** Female habitus **A** dorsal view **B** ventral view **C, D** Epigynum **C** ventral view **D** dorsal view; *Phintelloidesjesudasi* (**E–H**) **E, F** Female habitus **E** dorsal view **F** ventral view **G, H** Epigynum **G** ventral view **H** dorsal view. Scale bars: 1 mm (**A, B, E, F**), 0.1 mm (**C, D, G, H**).

**Figure 7.**
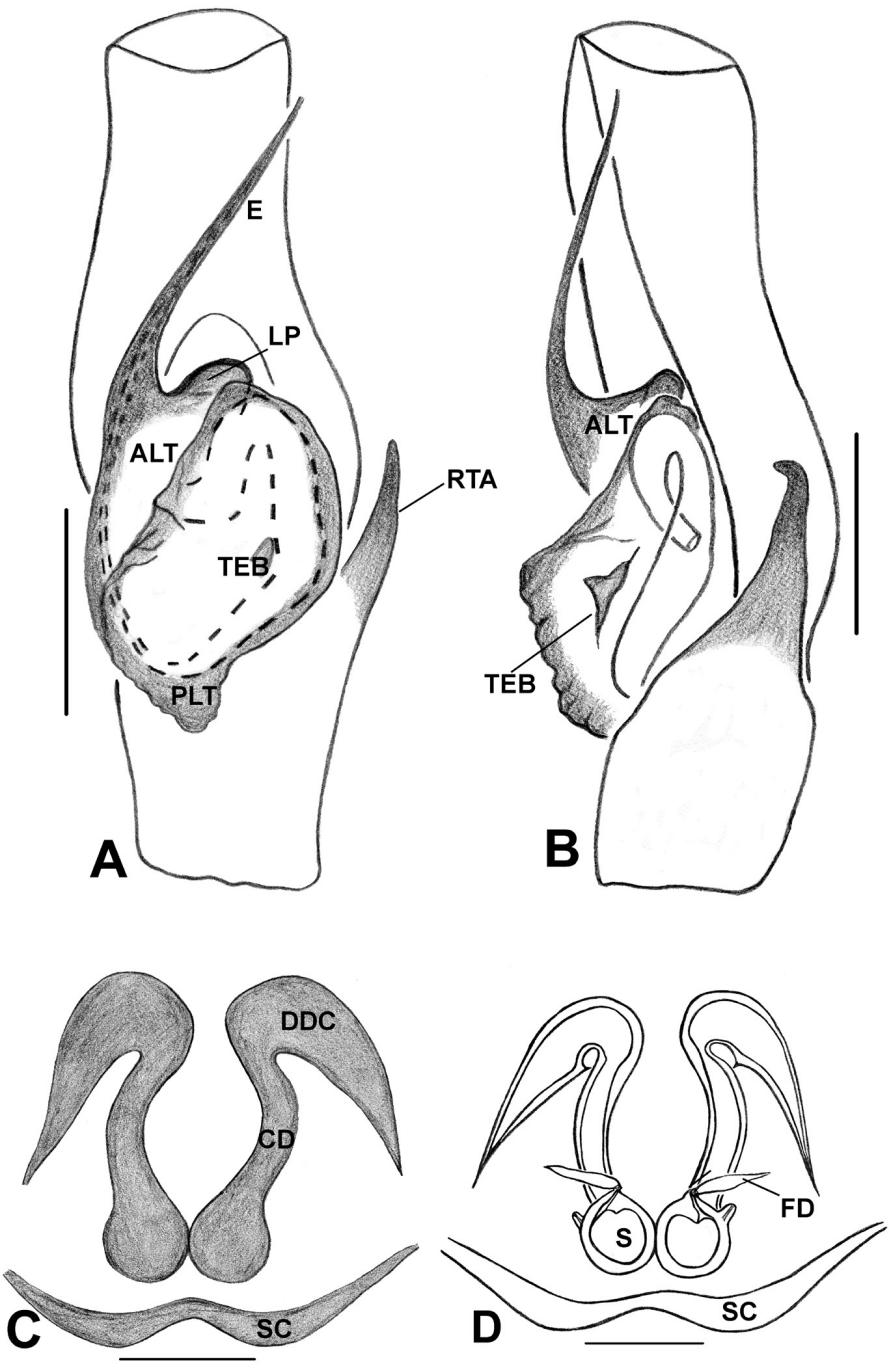
*Phintelloidesalborea***A** Palp, ventral view **B** Palp, retrolateral view **C** Epigynum, ventral view **D** Vulva, dorsal view. Abbreviations: ALT = apical lobe of tegulum; CD = copulatory ducts; CO = copulatory opening; DDC = duck-neck-shaped diverging curves; E = embolus; FD = fertilisation ducts; PLT = proximal lobe of tegulum; RTA = retrolateral tibial apophysis; S = spermatheca; SC = scapum; TEB = tegular bump. Scale bars: 0.2 mm (**A, B**), 0.1 mm (**C, D**).

The monophyly of *Phintelloides* is recovered in all ML molecular trees (except in the 18S single gene analysis; see supporting information) and the morphology parsimony tree (Figs 1, 3). Supported by the following morphological unambiguous putative synapomorphies: triangular-shaped bulbus, slightly oblique to apical lobe (39–1) (Figs 5D, 7A, 9D, 11A, 15D, 16A, 17D, 18A), conspicuous black blotches on the prosoma of females (7–1) (Figs 4D, G, H, 12A, E, 14D–F), duck-neck-shaped diverging curves at anterior margin of epigynum (46–1) (Figs 6C, D, G, H, 7C, D, 10C, D, G, H, 11C, D, 12C, D, G, H, 13A–D, 16C, D, 18C, D), apical origin of FD (55–3). The genus *Phintelloides* is a member of the tribe Chrysillini both as defined by Maddison (2015) and Proszynski (2016).

All molecular trees recover *Phintelloidesbrunne* and *P.flavoviri* as sister species with high support. This is in contrast to the morphological tree where *P.orbisa* and *P.flavoviri* are recovered as sister species (*P.orbisa* was not included in the molecular analysis, due to lack of fresh materials). We predict that it would branch with *P.flavoviri*, due to similar genital morphology (Fig. 12G, H). All genes trees, individual single gene phylogenetic trees as well as male and female habitus, palpal, and epigynal structure suggest that *Chrysillajesudasi* Caleb & Mathai, 2014 should be transferred to *Phintelloides*.

####### Diagnosis.

This genus can be recognised from other chrysillines by white tuft of hairs on the clypeus, white diamond-shaped mark behind PLE, pale white band on the anterior eye field, black median band bordered by two lateral bands on the abdomen. Further, presence of LP, comparably longer embolus in males and the duck-neck-shaped diverging curves of CD in females. This genus is closely related to *Proszynskia* in appearance than to *Phintella* and *Chrysilla*.

####### Description.

Medium sized spiders. Male with white tuft of hairs on the clypeus (described as “moustache” in Caleb and Mathai (2014); prosoma with pale yellow/ white band behind AME; white diamond-shaped mark behind the eye field; white belts on lateral prosoma; leg I slightly robust in males; abdomen with blackish or brownish grey longitudinal median band bordered by pale yellow bands; long embolus; apical portion of bulbus with lamellar process; small posterior lobe of bulbus; long RTA with bent tip. Female with black patches on the eye field and surrounding PME, behind PLE and posterior slope of prosoma; abdomen with longitudinal lateral stripes or devoid of markings; duck-neck-shaped diverging curves at anterior margin of epigynum; CO laterally outwards; CD medium or very long and bent or twisted; spermatheca pyriform or spherical; broad PEB. See species descriptions below.

####### Composition.

*Phintelloidesalborea* sp. n., *P.brunne* sp. n., *P.flavoviri* sp. n., *P.flavumi* sp. n., *P.jesudasi* (Caleb & Mathai, 2014) comb. n., *P.orbisa* sp. n., *P.versicolor* (CL Koch, 1846) comb. n.

####### Remarks.

The transfer of *P.versicolor* is based on the tree from the ML phylogenetic analysis of the combined matrix (Fig. 1). Additionally, *P.versicolor* shares with other species of *Phintelloides* the following characters: in males, the lateral white belts of the prosoma, white band on the anterior eye field, white diamond mark, black longitudinal abdominal median band bordered with pale yellow bands and similar shape of tegulum. In females, it differs in the absence of black blotches of prosoma, stripe pattern of abdomen and absence of DDC of the epigynum. It is also not clear if all specimens described under this name belong to a single species; special attention needs to be given to this matter in future studies.

####### Distribution.

India, Sri Lanka (excluding *P.versicolor*).

###### Phintelloides alborea sp. n.

Taxon classificationAnimaliaAraneaeSalticidae

http://zoobank.org/2B69E627-8FD2-46E1-A891-7A526D7D862B

Figs 4A–D , 5A–E , 6A–D , 7A–D

####### Type material.

**Holotype**1♂ (IFS_SAL_436), Sri Lanka, Central Province, Matale District, IFS Arboretum, 188 m, 07°51'34"N, 80°40'28"E, 17-VIII-2012, leg. SP Benjamin et al. **Paratype.** 1♀ (IFS_SAL_369), same locality as holotype, 07-VII-2013, leg. SP Benjamin and N Athukorala. **Other material examined.** 1♀ (IFS_SAL_654), Sri Lanka, North Central Province, Anuradapura District, Mihintale Sanctuary, 123 m, 08°21'10.60"N, 80°30'14.54"E, hand collection, 22-VI-2013, leg. I Sandunika. 1♂, 1♀ (IFS_SAL_814-815), same locality and collection data, 14-VI-2016, leg. K Nilani.

####### Etymology.

The species name a noun in apposition, is derived from the Latin *alborem* for pale white colour and refers to the pale white spots behind PMEs on the prosoma of males.

####### Diagnosis.

This species is distinguishable from other known congeners by oval LP in males (Figs 5D, 7A), small and rounded spermathecae and shape of copulatory ducts (thin, elongated without coils) in females. This species is closely related to *P.jesudasi* comb. n. and *P.flavumi* by the shape of bulbus and RTA (Figs 5D, 15D, 17D); however, female members obviously differs by rounded spermathecae, ‘c’-shaped CD and comparably broader DDC (Figs 6C, D, G, H, 10C, D). Furthermore, it differs from *P.brunne* and *P.versicolor* by its comparably longer embolus and broader ALT, and from *P.orbisa* and *P.flavoviri* by the absence of a coiled CD.

####### Description.

Male. Blackish clypeus with white ‘moustache’ covered with tuft of white hairs (Fig. 4B). Prosoma blackish ornamented with pale yellow band behind AME (Fig. 4A) and slightly broader than abdomen. There are two white blotches in front of PLE in life. Conspicuous, constricted, white diamond-shaped mark behind eye field. Lateral sides of prosoma with white belts (Figs 4A, B). Chelicerae brownish black, covered with white hairs at its base. Yellowish brown prosoma with black patches behind ALE and PLE in preserved specimens. Posterior margin of prosoma rather steep and slightly truncated. Sternum yellowish brown and oval in shape, edges bordered with light brown. Leg I robust than others, leg I and II blackish with white hairs around proximal region of patella, tibia and metatarsus, leg III and IV blackish yellow. Medium sized abdomen, tapering posteriorly. Dorsum with broad blackish grey median band, bordered by pale yellow bands extending longitudinally from anterior to posterior end (Fig. 4A). Ventrum blackish grey in life and yellowish brown in preserved specimens (Fig. 5B). Spinnerets yellow.

Palp covered with pale yellow scales, except for reddish brown cymbium. Cymbium longer and narrower at the distal region and broader at the proximal region. Embolus slender, long immovable on rather broad apical portion of bulbus (Figs 5C–E, 7A, B). Lamellar process semicircular comparably smaller than in *P.flavumi* (Figs 5D, 7A). Bulbus longer than wide. Sperm duct is clearly visible at the distal portion of tegulum. Small triangular bump at the retrolateral portion of bulbus. Tegulum with small posterior lobe (Figs 5C, D, 7A). RTA robust and long, nearly half-length of the bulbus, broader at the base, narrower and bent at the tip (Figs 5D, E, 7A, B).

**Measurements.**TL 4.60, PL 2.20, PW at PLE 1.70, AL 2.24, AW 1.26. Eye field: diameter of AME 0.50, PLE 0.32, ALE 0.27, PME 0.15, PME-PME 1.25, PLE-PLE 0.67, ALE-PME 0.30, ALE-PLE 0.71. Leg I: TR 0.34, FM 2.20, PT 0.90, TB 2.10, MT 1.64, TA 0.76; Leg II: TR 0.25, FM 1.68, PT 0.72, TB 1.40, MT 0.83, TA 0.81; Leg III: TR 0.34, FM 1.92, PT 0.76, TB 1.30, MT 1.22, TA 0.53; Leg IV: TR 0.31, FM 1.86, PT 0.66, TB 1.32, MT 1.54, TA 0.66.

**Female.** White prosoma decorated with three pairs of large, black patches, surrounding PME, behind PLE and posterior slope of prosoma in life (Figs 4D, 6A). Eye field enclosed with pale yellow scales. AME blackish brown covered with white and yellowish scales in the anterior and posterior portions respectively. Clypeus covered with dense white scales (Fig. 4C). Chelicerae unidentate, pale brown. In alcohol-preserved specimen, carapace yellow with faded black patches. Sternum yellow and pentagonal in shape (Fig. 6B).

Abdomen pale yellow and elliptically shaped, longer and narrower than prosoma. Dorsum with two lateral greenish black stripes extending longitudinally along the length of the abdomen (Figs 4D, 6A). Ventrum enclosed with white scales, no markings in live specimens (Fig. 4D). Spinnerets pale yellow. Legs glassy pale yellow.

Epigynum moderately sclerotised. CO projecting laterally outwards (Fig. 6D). Copulatory ducts diverge initially at more than two receptacle diameters and then slightly curved inward to form DDC leading to CO (Figs 6C, D, 7C, D). Spermathecae comparably small, rounded and both receptacles placed closely to each other (Figs 6C, D, 7C, D). FD lanceolate, originating from apical wall of receptacles (Figs 6D, 7D). PEB rather broad with shallow median indentation.

**Measurements.**TL 4.82, PL 2.52, PW at PLE 1.82, AL 2.35, AW 1.38. Eye field: diameter of AME 0.51, PLE 0.30, ALE 0.27, PME 0.15, PME-PME 1.20, PLE-PLE 0.67, ALE-PME 0.33, ALE-PLE 0.68. Leg I: TR 0.27, FM 2.22, PT 1.00, TB 1.79, MT 1.60, TA 0.71; Leg II: TR 0.28, FM 1.62, PT 0.75, TB 1.40, MT 0.84, TA 0.80; Leg III: TR 0.32, FM 1.80, PT 0.77, TB 1.25, MT 1.22, TA 0.54; Leg IV: TR 0.30, FM 1.78, PT 0.67, TB 1.27, MT 1.51, TA 0.66.

####### Distribution.

This species is known only from Sri Lanka.

###### Phintelloides brunne sp. n.

Taxon classificationAnimaliaAraneaeSalticidae

http://zoobank.org/CE3422DF-5A3B-4C9B-B0BD-02CE2C7055FF

**Figure 8.**
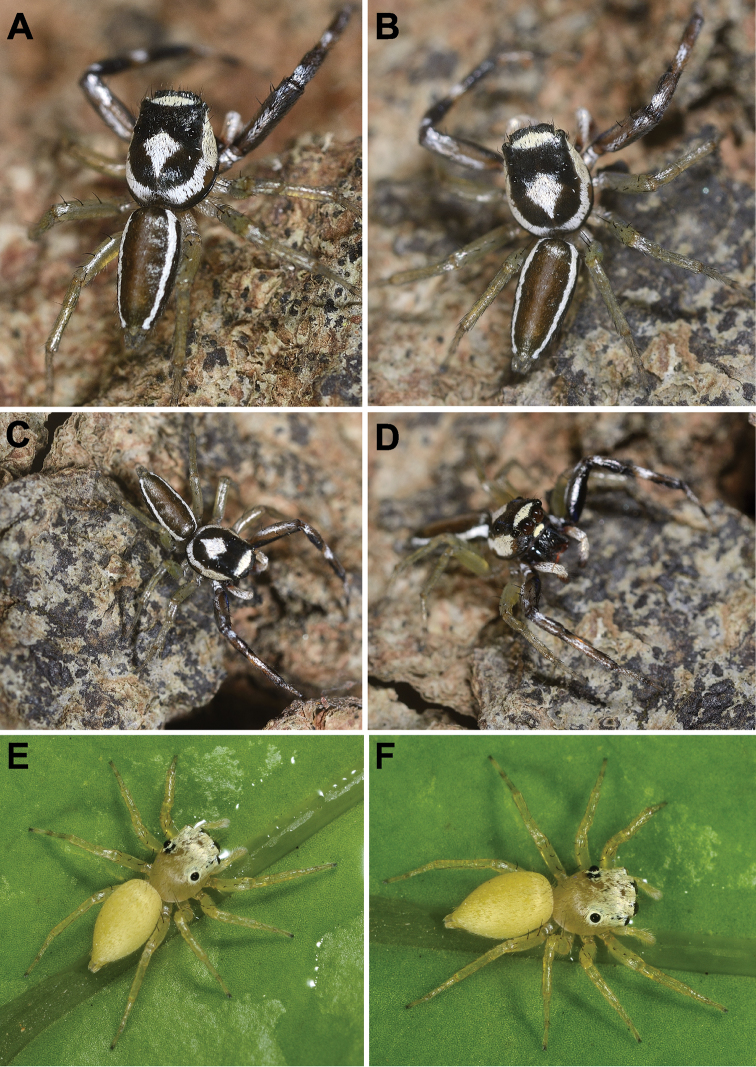
Live male of *Phintelloidesbrunne* (**A–D**) from Hakgala SNR and female of *Phintelloidesflavoviri* (**E, F**) from Hiyare, Kombala-Kottawa FR.

**Figure 9.**
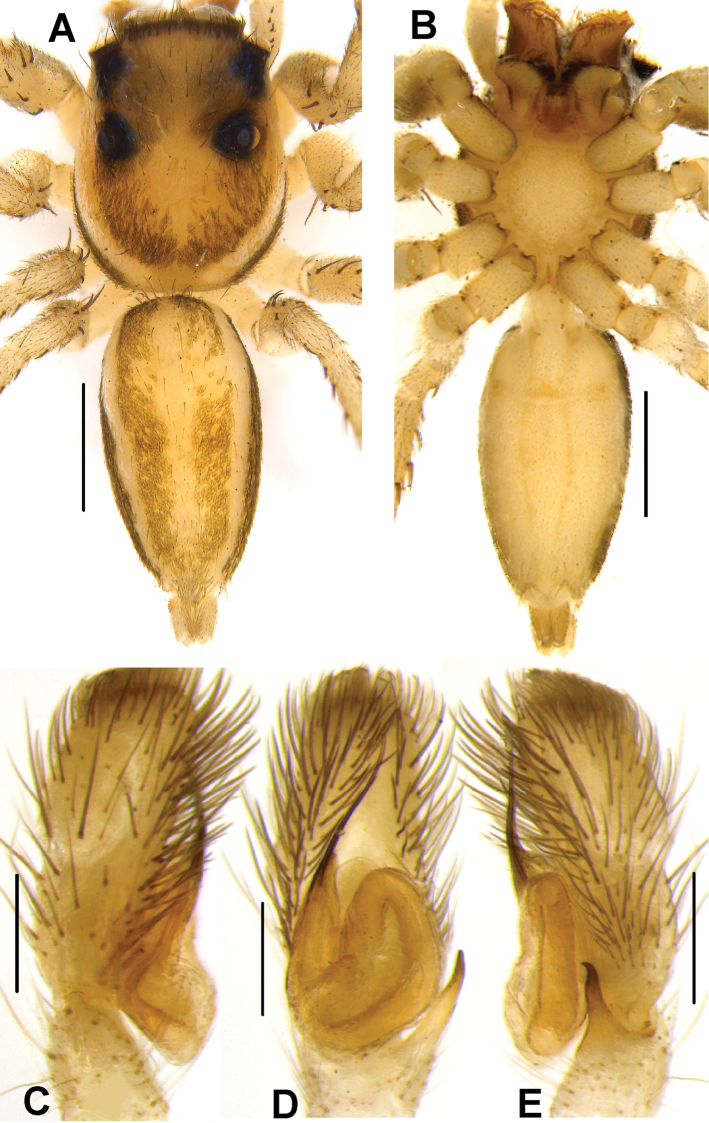
*Phintelloidesbrunne* (**A, B**). Male habitus **A** dorsal view **B** ventral view **C–E** Male palp **C** prolateral view **D** ventral view **E** retrolateral view. Scale bars: 1 mm (**A, B**), 0.2 mm (**C–E**).

**Figure 10.**
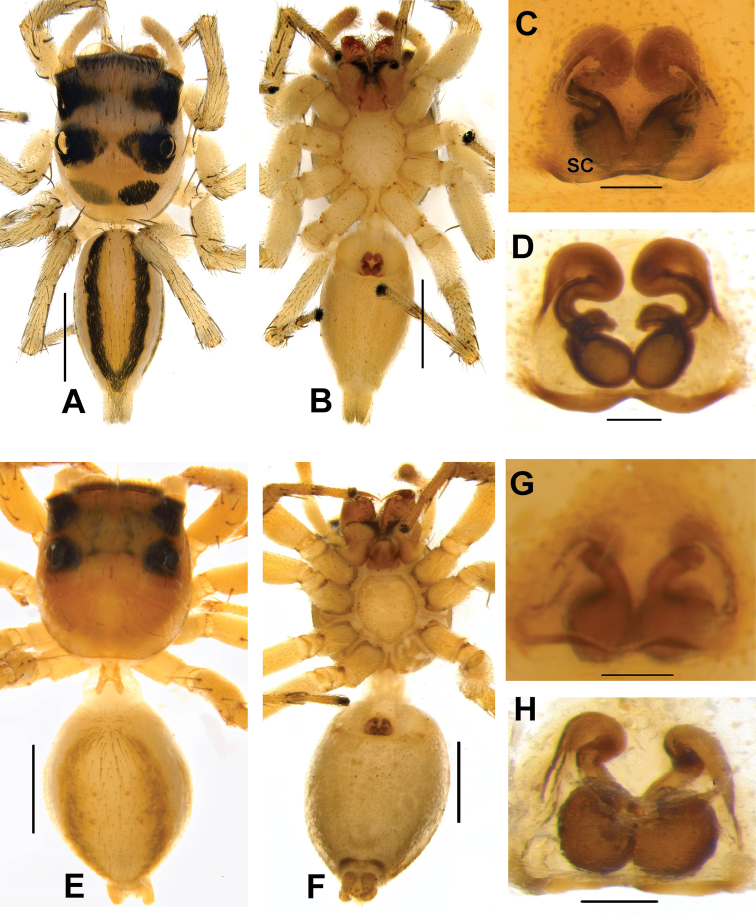
*Phintelloidesflavumi* (**A–D**) **A–B** Female habitus **A** dorsal view **B** ventral view **C–D** Epigynum **C** ventral view **D** dorsal view; *Phintelloidesbrunne* (**E–H**) **E, F** Female habitus **E** dorsal view **F** ventral view **G, H** Epigynum **G** ventral view **H** dorsal view. Abbreviation: SC = scapum. Scale bars: 1 mm (**A, B, E, F**), 0.1 mm (**C–D**), 0.1 mm (**G, H**).

**Figure 11.**
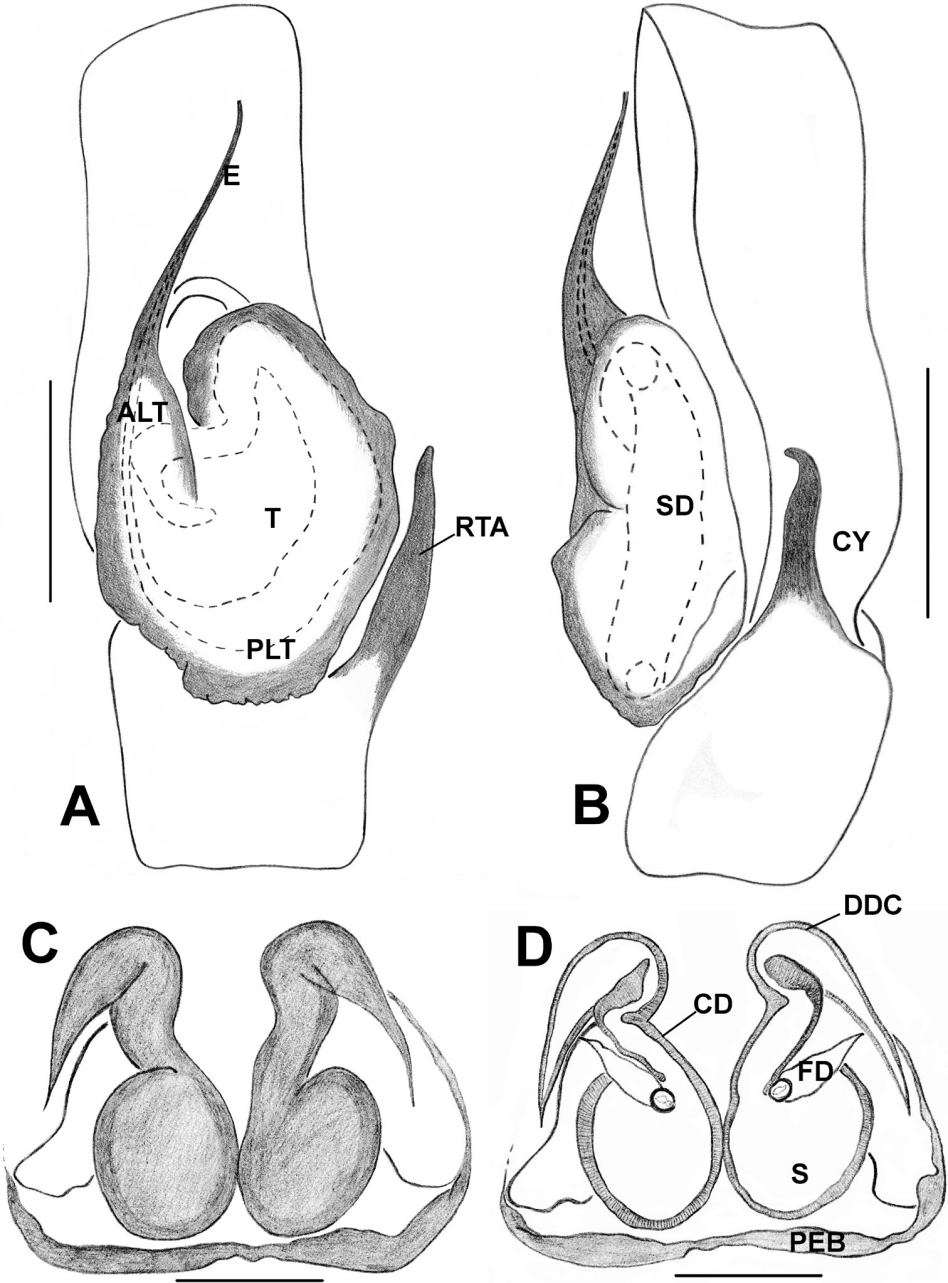
*Phintelloidesbrunne***A** Palp, ventral view **B** Palp, retrolateral view **C** Epigynum, ventral view **D** Vulva, dorsal view. Abbreviations: ALT = apical lobe of tegulum; CD = copulatory ducts; CY = cymbium; DDC = duck-neck-shaped diverging curves; E = embolus; FD = fertilisation ducts; PEB = posterior epigynal border; PLT = proximal lobe of tegulum; RTA = retrolateral tibial apophysis; S = spermatheca; SD = sperm duct; T = tegulum. Scale bars: 0.2 mm (**A, B**), 0.1 mm (**C, D**).

Figs 8A–D , 9A–E , 10E–H , 11A–D

####### Type material.

**Holotype** ♂ (IFS_SAL_142): Sri Lanka, Central Province, Kandy District, Delthota, Loolkandura FR, 1480m, hand collection, 07°08'45"N, 80°41'53"E, 11-V-2010, leg. S Batuwita and N Athukorala. **Paratype.** ♀ (IFS_SAL_281): same locality, Matale disrtict, Gammaduwa, Knuckles range, 900 m, 18-XI-2009, Hand collection, leg. SP Benjamin, S Batuwita et al. **Other material examined.** 1♂ (IFS_SAL_844): same locality, Nuwara Eliya District, Hakgala SNR, 1745 m, 06°55'00"N, 80°46'00"E, beating, 30-VI-2016, leg. K Nilani. (IFS_SAL_256): same locality, Matale District, Knuckles range Deenston K06/7/9, beating, 11/12-III-1998, leg. SP Benjamin.

####### Etymology.

This species name a noun in apposition, derived from the Latin *brunneus*, and refers to the reddish brown colouration of median band of the dorsal abdomen.

####### Diagnosis.

This species is distinguishable from other known congeners by colour of the median band of abdomen and broad PLT in males (Fig. 8A–C), and the relatively large spermathecae and shape of copulatory ducts (thick, stout) in females. It differs from *P.alborea*, *P.jesudasi*, and *P.flavumi* by comparably shorter embolus, narrower triangular ALT and from *P.versicolor* by comparably longer embolus, narrow ALT and short RTA. Female members obviously differ from *P.orbisa* and *P.flavoviri* by the absence of coiled CD.

####### Description.

Male. Live spiders, clypeus enclosed with tuft of white scales, prosoma blackish brown decorated with pale yellow band on the anterior margin of prosoma behind AME. White dots sparsely scattered on prosoma (Fig. 8A–D). A white, prominent diamond-shaped mark behind PLE (Fig. 8A–C). Lateral sides of prosoma with white belts (Fig. 8A–C). In ethanol preserved specimens, chelicerae brown, prosoma yellowish brown. Posterior margin of prosoma slightly truncated (Fig. 9A). Rounded, yellowish brown sternum, edges light brown (Fig. 9B).

Abdomen moderately long and slightly narrower than prosoma, tapering posteriorly. Dorsum with much broader greenish brown median band, delimited by narrow white lateral bands extending longitudinally from anterior to posterior end (Fig. 8A–C). Ventrum blackish grey in life and pale yellow in preserved specimens. Spinnerets greyish brown. Leg I blackish brown and robust than others, leg II, III and IV blackish yellow.

Yellowish brown palp. Cymbium shorter than *P.jesudasi* and slightly narrower distally. Embolus slender, long immovable on the thinner apical portion of tegulum, slightly extending beyond the level of the distal end of tegulum (Figs 9C–E, 11A, B). Lamellar process absent. Bulbus longer than wide. Spermatophore loop clearly visible at the antero-lateral portion of bulbus. Tegulum with small posterior lobe. RTA nearly half-length of bulbus, broader at base, narrow tip hook-shaped (Figs 9D, E, 11A, B).

**Measurements.**TL 4.60, PL 1.85, pW at PLE 1.50, AL 2.30, AW 1.15. Eye field: diameter of AME 0.43, PLE 0.16, ALE 0.24, PME 0.01, PME-PME 1.13, PLE-PLE 1.10, ALE-PME 0.03, ALE-PLE 0.65. Leg I: TR 0.27, FM 1.13, PT 0.81, TB 1.30, MT 0.95, TA 0.41; Leg II: TR 0.24, FM 1.13, PT 0.46, TB 0.81, MT 0.68, TA 0.41; Leg III: TR 0.24, FM 1.22, PT 2.03, TB 0.92, MT 0.98, TA 0.43; Leg IV: TR 0.22, FM 1.16, PT 0.51, TB 1.00, MT 1.10, TA 0.43.

**Female.** Ethanol preserved specimens, prosoma yellowish brown, ALE, PME, PLE covered with black blotches (Fig. 10E, F). Chelicerae, labium yellowish brown, edges brown. Pentagon-shaped sternum, pale yellow, edges light brown (Fig. 10F). Posterior prosoma steep and slightly truncated. Leg I dark yellowish brown, slightly robust than rests. Legs II, III, and IV yellow.

Abdomen moderately long and slightly broader than prosoma, tapering posteriorly. Dorsum with two yellowish brown stripes extending longitudinally from anterior portion to near spinnerets (Fig. 10E). Middle of dorsum decorated with two pairs of dark yellowish brown spots. Ventrum pale brown. Spinnerets yellow.

Epigynum moderately sclerotised. DDC is shorter compared to its congeners (Figs 10G, H, 11C, D). Position of CO unclear, could be laterally outwards. Comparably shorter CD is twisted near DDC. Spermathecae large and rounded (Fig. 10G, H). FD lanceolate originating from apical portion of receptacles (Figs 10H, 11D). PEB comparably thinner than other congeners.

**Measurements.**TL 4.72, PL 2.15, PW at PLE 1.48, AL 2.55, AW 2.10. Eye field: diameter of AME 0.43, PLE 0.16, ALE 0.24, PME 0.01, PME-PME 1.10, PLE-PLE 1.15, ALE-PME 0.03, ALE-PLE 0.65. Leg I: TR 0.25, FM 1.12, PT 0.78, TB 1.32, MT 0.90, TA 0.36; Leg II: TR 0.26, FM 1.20, PT 0.44, TB 0.78, MT 0.68, TA 0.40; Leg III: TR 0.25, FM 1.20, PT 2.00, TB 0.87, MT 0.96, TA 0.40; Leg IV: TR 0.25, FM 1.10, PT 0.54, TB 1.00, MT 1.20, TA 0.40.

####### Distribution.

This species is known only from Sri Lanka.

###### Phintelloides flavoviri sp. n.

Taxon classificationAnimaliaAraneaeSalticidae

http://zoobank.org/9ED1B07B-33C6-4C36-B2E7-38DCC9285F74

**Figure 12.**
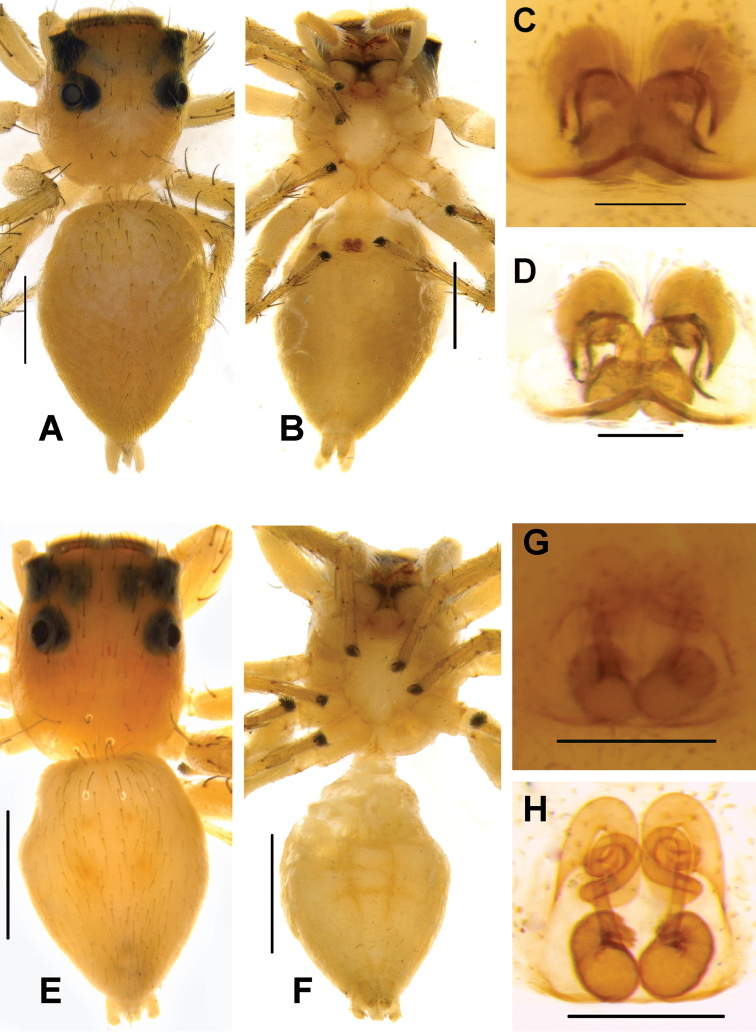
*Phintelloidesflavoviri* (**A–D**) **A, B** Female habitus **A** dorsal view **B** ventral view **C, D** Epigynum **C** ventral view **D** dorsal view; *Phintelloidesorbisa* (**E–H**) **E, F** Female habitus **E** dorsal view **F** ventral view **G, H** Epigynum **G** ventral view **H** dorsal view. Scale bars: 1 mm (**A, B, E, F**), 0.1 mm (**C–D**), 0.2 mm (**G, H**).

**Figure 13.**
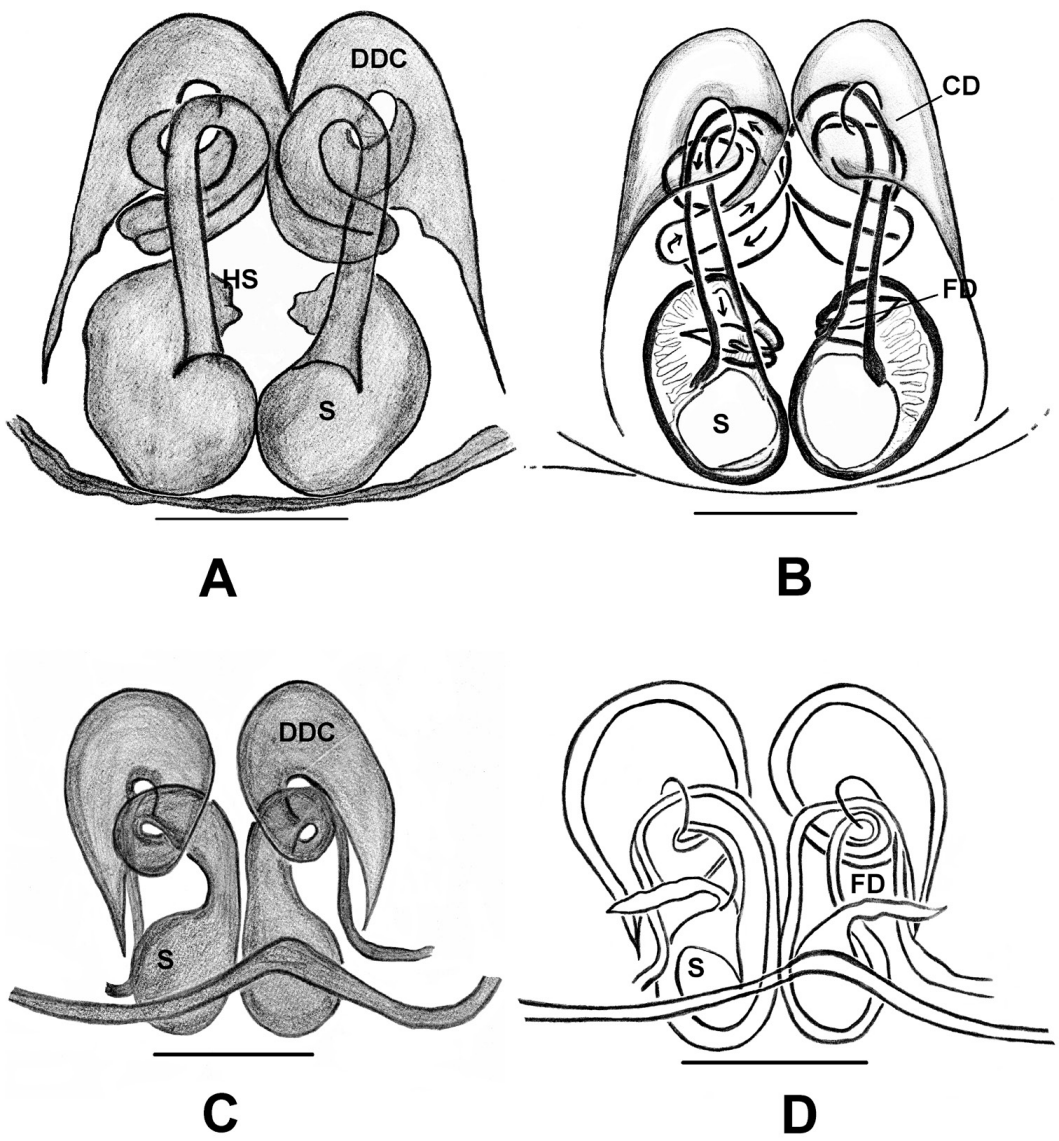
*Phintelloidesorbisa* (**A, B**) and *Phintelloidesflavoviri* (**C, D**) **A, C** Epigynum, ventral view **B, D** Vulva, dorsal view. Abbreviations: CD = copulatory ducts; DDC = duck-neck-shaped diverging curves; FD = fertilisation ducts; HS = head like structure; S = spermatheca. Scale bars: 0.1 mm (**A–D**).

Figs 8E–F , 12A–D , 13C–D

####### Type material.

**Holotype** ♀ (IFS_SAL_754): Sri Lanka, Southern Province, Galle District, Hiyare, Kombala-Kottawa FR, 252 m, 06°03'53"N, 80°18'05"E, beating, 24–26-V-2016, leg. K Nilani. **Paratype.** ♀ (IFS_SAL_755): same locality and collection data as in holotype.

####### Etymology.

The species name a noun in apposition, is derived from the Latin *flavo viridi* and refers to the uniform yellowish green colour body of females.

####### Diagnosis.

This species is distinguishable from other known congeners by the single coiled, comparably broader CD, sclerotised long projections from DDC and oval spermathecae (Figs 12C, D, 13C, D). It is closely related to *P.orbisa* (Figs 8E, F) both possess a twisted CD with coils (which are longer in *P.orbisa*) and broader DDC; however, it differs by the rounded spermathecae, single coil of CD, and sclerotised structures of CD.

####### Description.

Female. Prosoma greenish yellow, ocular region with dense white hairs. AME and ALE greenish black in life (Fig. 8E, F). Pale yellow prosoma with black blotches on the ocular region in preserved specimens (Fig. 12A). PLE and PME surrounded with black patches. Posterior prosoma with devoid of any markings. Posterior margin of prosoma slightly truncated. Sternum pale yellow, oval in shape.

Abdomen greenish yellow in life, pale yellow in preserved specimen, elliptical, broader, and longer than prosoma. Dorsum devoid of any longitudinal stripes or markings as in other congeners (Fig. 8E, F). Ventrum pale yellow without any markings. Spinnerets pale yellow.

Epigynum moderately sclerotised. CO indistinct. DDC are broader, twice width of CD at anterior margin (Figs 12C, D, 13C, D). CD broader, longer, straight initially, twisted with one coil near DDC. Spermathecae rounded, thick walled, placed closely to each other. FD lanceolate, arising from anterior wall of receptacles (Fig. 13D). PEB comparably thinner than its congeners.

**Measurements.**TL 4.80, PL 1.92, PW at PLE 1.44, AL 2.56, AW 1.84. Eye field: diameter of AME 0.43, PLE 0.15, ALE 0.28, PME 0.01, PME-PME 1.15, PLE-PLE 1.17, ALE-PME 0.03, ALE-PLE 0.66. Leg I: TR 0.28, FM 1.14, PT 0.54, TB 0.87, MT 0.64, TA 0.47; Leg II: TR 0.26, FM 1.10, PT 0.47, TB 0.80, MT 0.60, TA 0.40; Leg III: TR 0.25, FM 1.34, PT 0.60, TB 0.81, MT 0.53, TA 0.40; Leg IV: TR 0.26, FM 1.47, PT 0.67, TB 0.94, MT 0.67, TA 0.60.

**Male.** Unknown.

####### Distribution.

This species is known only from Sri Lanka.

###### Phintelloides flavumi sp. n.

Taxon classificationAnimaliaAraneaeSalticidae

http://zoobank.org/5BF3DAD5-E532-4DA1-BDB4-A8C72A4E8DC6

**Figure 14.**
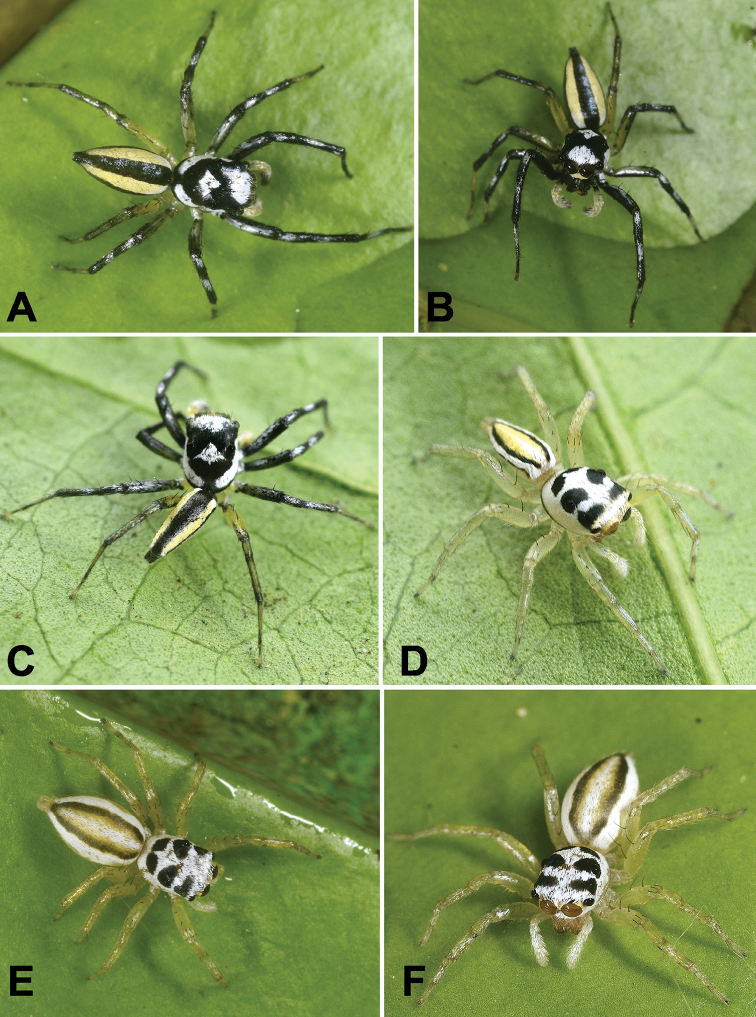
*Phintelloidesflavumi***A, B** Male from Hiyare, Kombala-Kottawa FR**C** Male from Singaraja FR, Kudawa **D** Female from Singaraja FR, Kudawa **E, F** Female from Hiyare, Kombala-Kottawa FR.

**Figure 15.**
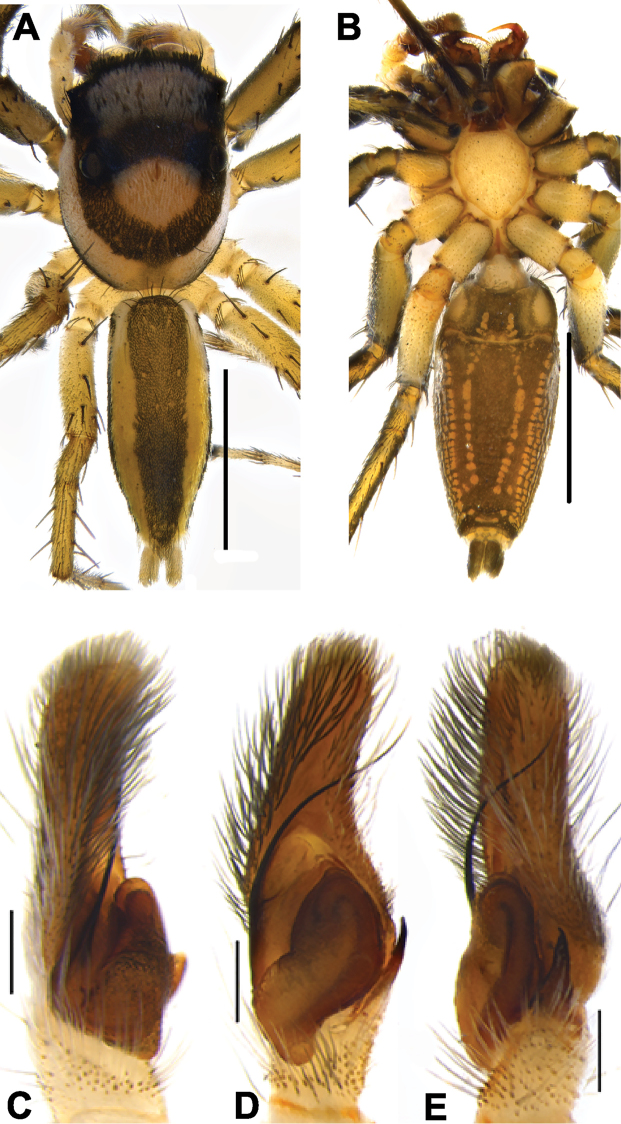
*Phintelloidesflavumi* (**A, B**). Male habitus **A** dorsal view **B** ventral view **C–E** Male palp **C** prolateral view **D** ventral view **E** retrolateral view. Scale bars: 2 mm (**A, B**), 0.2 mm (**C–E**).

**Figure 16.**
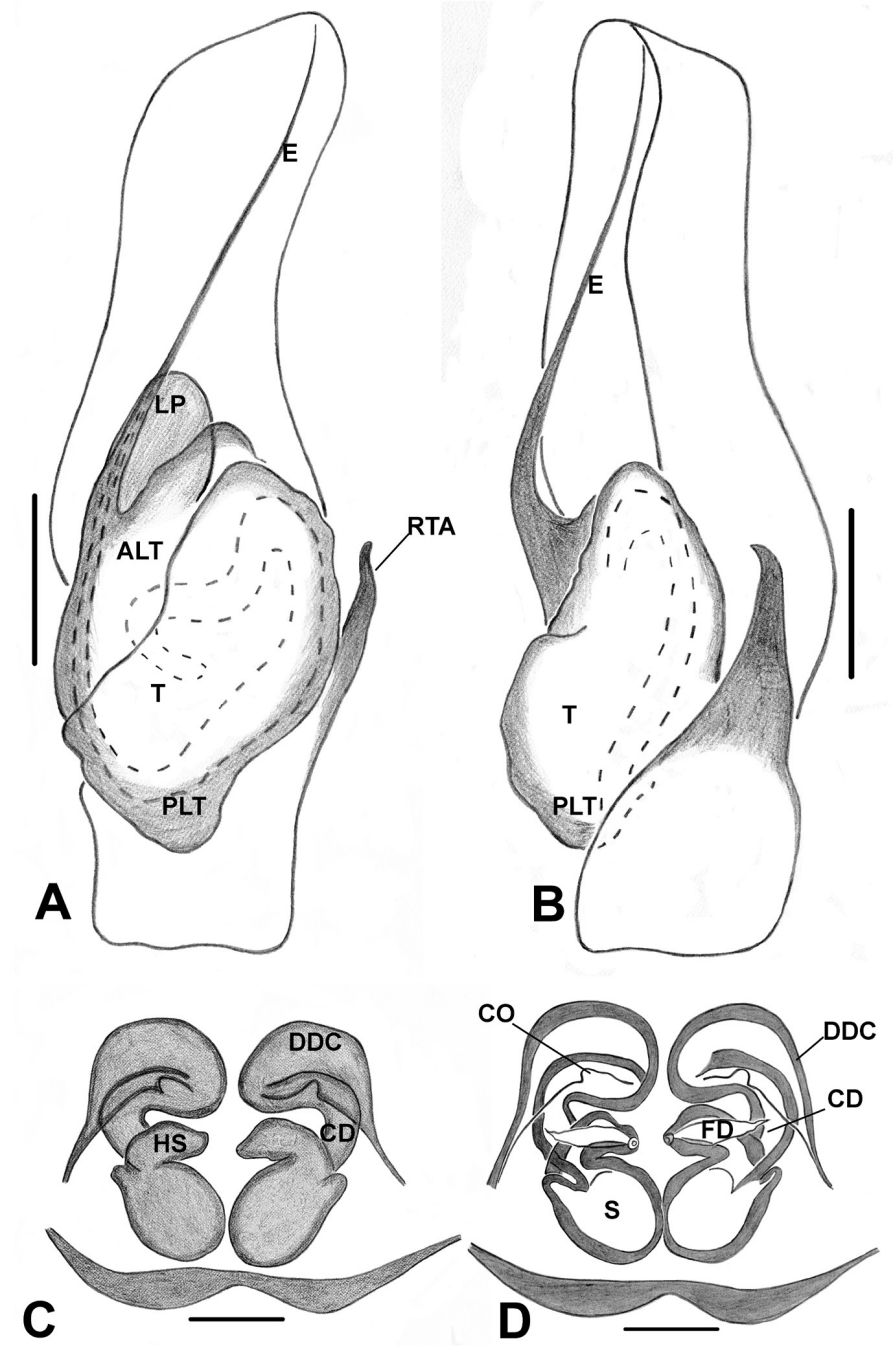
*Phintelloidesflavumi***A** Palp, ventral view **B** Palp, retrolateral view **C** Epigynum, ventral view **D** Vulva, dorsal view. Abbreviations: ALT = apical lobe of tegulum; CD = copulatory ducts; CO = copulatory opening; DDC = duck-neck-shaped diverging curves; E = embolus; FD = fertilisation ducts; HS = head like structure; LP = lamellar process; PLT = proximal lobe of tegulum; RTA = retrolateral tibial apophysis; S = spermatheca; T = tegulum. Scale bars: 0.2 mm (**A–B**), 0.1 mm (**C–D**).

Figs 10A–D , 14A–F , 15A–E , 16A–D

####### Type material.

**Holotype** ♂ (IFS_SAL_758): Sri Lanka, Southern Province, Galle District, Hiyare, Kombala-Kottawa FR, 252m, 06°03'53"N, 80°18'05"E, beating, 24–26-V-2016, leg. K Nilani and I Sandunika**. Paratype.** ♀ (IFS_SAL_760): Same locality and collection data as holotype. **Other material examined.** 1♀ (IFS_SAL_689): same locality and collection data as holotype, 18-V-2010, leg. SP Benjamin and S Batuwita. 3♂, 2♀ (IFS_SAL_742-746): Same locality and collection data as holotype. 2♂, 1♀ (IFS_SAL_970-972): Sabaragamuwa Province, Rathnapura District, Sinharaja FR, Kudawa, 521 m, 06°24'58.26"N, 80°25'25"E, beating, 11–13-X-2016, leg. K Nilani.

####### Etymology.

The species name a noun in apposition, is derived from the Latin *flavum* and refers to the yellow coloured scales around AME.

####### Diagnosis.

The species is distinguishable from other congeners by the rounded, well-developed LP, longer, straight, tapering RTA in males (Figs 15D, E, 16A, B) and inverted C-shaped CD, well-developed HS (Figs 10C, 16C), and relatively smaller body size in females. It is closely related to *P.alborea* and *P.jesudasi* in palpal structure with minor differences including semicircular LP, straight RTA. However, it obviously differs by well-developed HS and inverted C-shaped CD.

####### Description.

Male. In life, prosoma black, decorated with white band in the vicinity of first row of eyes at the anterior margin, clypeus with dense white scales (Fig. 14B). Chelicerae black, with white hairs at its base. Behind PLE, white, prominent diamond-shaped marks present (Fig. 14A–C). Lateral sides of prosoma with white belts (Fig. 14A–C). Posterior margin of prosoma rather steep and slightly truncated. Yellowish brown sternum, oval in shape, edges light brown. First pair of legs blackish and sturdy, other legs yellowish black.

Abdomen oval, moderately long, slightly narrower than prosoma, tapering posteriorly. Dorsum with comparably narrow black, median band, bordered by pale yellow bands extending longitudinally from anterior to posterior end (Fig. 14A–C). Ventrum blackish grey in life and brown with yellowish brown dots longitudinally, arranged from epigastric furrow to spinnerets in preserved specimens (Fig. 15B). Spinnerets black.

Moderately sclerotised pale yellow palp. Distal end of cymbium longer, narrower than proximal region. Embolus slender, long immovable, on rather broad apical portion of bulbus, extending to distal end of cymbium (Figs 15D, E, 16A, B). Lamellar process well developed, large, lying immediately below embolus (Figs 15D, 16A). Bulbus longer than wide. Sperm duct clearly visible at the antero-lateral portion of bulbus. Retrolateral portion of bulbus with small bump. Tegulum with posterior lobe. RTA long nearly half-length of the bulbus, broader at the base, narrower, hook-shaped at the tip (Figs 15D–E, 16A, B).

**Measurements.**TL 4.70, PL 2.20, PW at PLE 1.52, AL 2.40, AW 1.30. Eye field: diameter of AME 0.51, PLE 0.33, ALE 0.25, PME 0.12, PME-PME 1.20, PLE-PLE 0.66, ALE-PME 0.32, ALE-PLE 0.66. Leg I: TR 0.35, FM 2.25, PT 1.00, TB 1.88, MT 1.54, TA 0.81; Leg II: TR 0.24, FM 1.72, PT 0.76, TB 1.50, MT 0.84, TA 0.81; Leg III: TR 0.34, FM 1.87, PT 0.80, TB 1.24, MT 1.21, TA 0.50; Leg IV: TR 0.32, FM 1.78, PT 0.63, TB 1.33, MT 1.57, TA 0.60.

**Female.** Prosoma sparsely covered with white hairs and decorated with three pairs of large, black blotches, surrounding PME, PLE, ocular area, and posterior slope of prosoma (Fig. 14D–F). Pale brown AME covered with white hairs anteriorly and yellowish hairs posteriorly (Fig. 14F). Clypeus enclosed with dense white scales. Chelicerae unidentate, orange brown. Sternum yellow, with pale yellow hairs. In ethanol preserved specimen, carapace yellow with black patches.

Abdomen white, leaf-shaped, longer, slightly broader than prosoma. Dorsum with two lateral dark green streaks, elongating longitudinally along the whole length of the abdomen (Figs 14D–F). Ventrum with white scales, devoid of any markings in life. Spinnerets pale yellow. Legs glassy greenish yellow.

Epigynum moderately sclerotised. Copulatory openings located laterally inside duck-neck-shaped diverging curves. Copulatory ducts twisted, diverge initially and then bending inward to form a much broader duck-neck-shaped diverging curves leading to the copulatory openings (Figs 10C, D, 16C, D). Comparably large, oval spermathecae with head-like structure placed closely to each other (Figs 13D, 16C, D). Fertilisation ducts lanceolate originating from apical wall of receptacles (Figs 13D, 16D). Posterior epigynal border rather broad.

**Measurements.**TL 4.63, PL 2.11, PW at PLE 1.88, AL 2.50, AW 1.32. Eye field: diameter of AME 0.52, PLE 0.33, ALE 0.25, PME 0.12, PME-PME 1.22, PLE-PLE 0.66, ALE-PME 0.35, ALE-PLE 0.68. Leg I: TR 0.27, FM 2.14, PT 0.91, TB 1.84, MT 1.62, TA 0.70; Leg II: TR 0.28, FM 1.65, PT 0.70, TB 1.40, MT 0.85, TA 0.84; Leg III: TR 0.34, FM 1.82, PT 0.74, TB 1.23, MT 1.24, TA 0.55; Leg IV: TR 0.30, FM 1.84, PT 0.66, TB 1.26, MT 1.54, TA 0.66.

####### Distribution.

This species is known only from Sri Lanka.

###### Phintelloides jesudasi

Taxon classificationAnimaliaAraneaeSalticidae

(Caleb & Mathai, 2014) comb. n.

**Figure 17.**
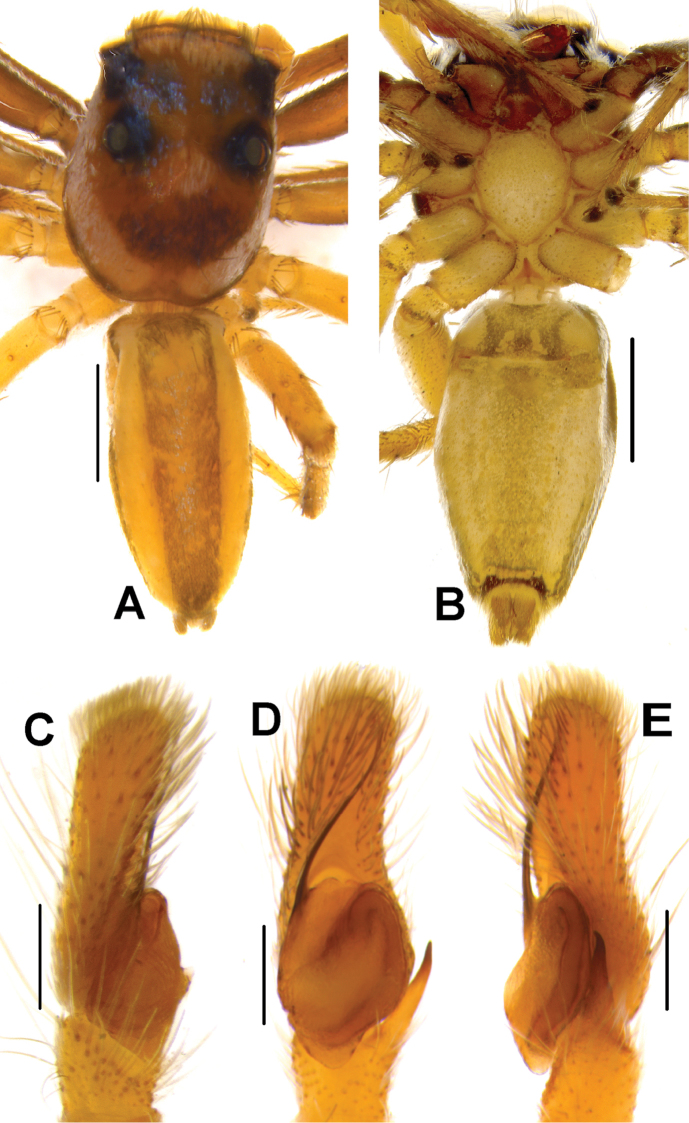
*Phintelloidesjesudasi* (**A, B**). Male habitus **A** dorsal view **B** ventral view **C–E** Male palp **C** prolateral view **D** ventral view **E** retrolateral view. Scale bars: 1 mm (**A, B**), 0.2 mm (**C–E**).

**Figure 18.**
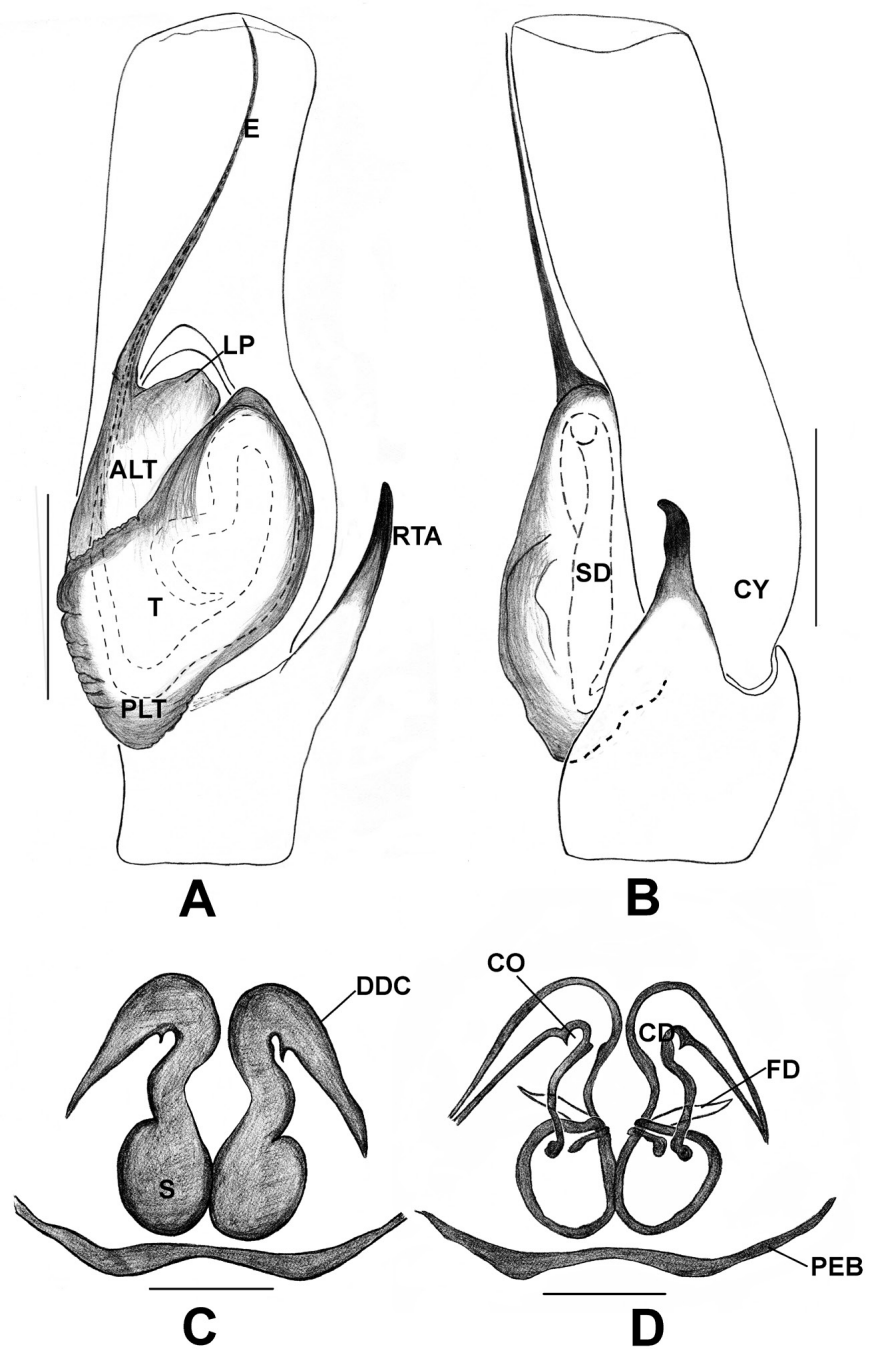
*Phintelloidesjesudasi***A** Palp, ventral view **B** Palp, retrolateral view **C** Epigynum, ventral view **D** Vulva, dorsal view. Abbreviations: ALT = apical lobe of tegulum; CD = copulatory ducts; CO = copulatory opening; CY = cymbium; DDC = duck-neck-shaped diverging curves; E = embolus; FD = fertilisation ducts; LP = lamellar process; PEB = posterior epigynal border; PLT = proximal lobe of tegulum; RTA = retrolateral tibial apophysis; S = spermatheca; SD = sperm duct; T: = tegulum. Scale bars: 0.2 mm (**A, B**), 0.1 mm (**C, D**).

Figs 4E–H , 6E–H , 17A–E , 18A–D

Chrysilla jesudasi Caleb & Mathai, 2014b: 63, figs 1–14.

####### Material examined.

1♂ (IFS_SAL_668), Sri Lanka, North Western Province, Kurunagala District, Ethagala FR, hand collection, 1–28-VII-2007, leg. Z Jaleel. 1♀ (IFS_SAL_137), same locality, 07°29'11.23"N, 80°22'21.64"E, 190 m, hand collection, 1-28-II-2008, leg. Z Jaleel. 1♂ (IFS_SAL_293), same locality, Polgahawala, hand collection, 14-VI-2015, leg. HMSM Nadeeshani. 1♂ (IFS_SAL_324), Uva Province, Badulla District, Diyaluma falls, 660 m, 06°43'57"N, 81°01'58"E, 04-VII-2012, leg. SP Benjamin. 4♂, 1♀ (IFS_SAL_920-924), Western Province, Gampaha District, Pilikuttuwa FR, 69 m, 07°03'52.4"N, 80°03'04"E, beating, 28-IX-2016, leg. K Nilani and I Sandunika.

####### Diagnosis.

This species is easily distinguishable from other known congeners by the irregular LP, stouter RTA and by the broad anterolateral portion of bulbus (Figs 17D, E, 18A, B) in males and shape of copulatory ducts, comparably thinner CD with space between DDC in females (Figs 6G, H, 18C, D). It is closely related to *P.alborea* and *P.flavumi* in palpal structure; however, it differs from them by irregular LP, broad ALT in males and comparably thinner DDC.

####### Description.

Male. In life, clypeus blackish with white stripe covered with tuft of white scales, cephalothorax blackish, with pale yellow band behind AME. White, prominent, diamond-shaped mark behind eye field (Fig. 4E, F). Lateral sides of prosoma with white belts (Fig. 4E, F; Caleb and Mathai 2014: figs 1, 3). Chelicerae reddish black, covered with white hairs at its base (Caleb and Mathai 2014: fig. 2). Prosoma fawn in colour, black patches behind ALE and around PLE in preserved specimens (Fig. 17A). Posterior margin of prosoma rather steep and slightly truncated. Yellowish brown sternum oval in shape, edges bordered with light brown (Fig. 17B). Leg I robust than others, legs I and II blackish with white hairs around proximal region of patella, tibia and metatarsus (Caleb and Mathai 2014), legs III and IV blackish yellow.

Abdomen moderately long, slightly narrower than prosoma, tapering posteriorly. Dorsum with broad blackish grey median band, surrounded by pale yellow bands, extending longitudinally from anterior to posterior end (Fig. 4E, F; Caleb and Mathai 2014: figs 1, 3). Ventrum blackish grey in life and yellowish brown in preserved specimens (Fig. 17B). Spinnerets yellow.

Pale yellow palp. Cymbium longer and narrower at the distal region. Embolus slender and long immovable on rather broad apical portion of bulbus (Figs 17C–E, 18A, B). Lamellar process is comparably smaller than in *P.alborea* (Figs 17D, 18A). Bulbus longer than wide. Spermatophore loop is clearly visible at the antero-lateral portion of bulbus. Retrolateral portion of bulbus with small bump. Tegulum with small posterior lobe. RTA long nearly half-length of the bulbus, broader at the base, narrower and curved at the tip (Figs 17D–E, 18A, B).

**Measurements.**TL 4.50, PL 2.10, PW at PLE 1.60, AL 2.30, AW 1.15. Eye field: diameter of AME 0.52, PLE 0.33, ALE 0.27, PME 0.12, PME-PME 1.22, PLE-PLE 0.67, ALE-PME 0.32, ALE-PLE 0.68. Leg I: TR 0.32, FM 2.15, PT 0.93, TB 1.90, MT 1.66, TA 0.74; Leg II: TR 0.26, FM 1.68, PT 0.71, TB 1.41, MT 0.81, TA 0.81; Leg III: TR 0.30, FM 1.80, PT 0.73, TB 1.25, MT 1.21, TA 0.51; Leg IV: TR 0.30, FM 1.83, PT 0.64, TB 1.29, MT 1.53, TA 0.63.

**Female.** Prosoma white decorated with three pairs of large, black patches, surrounding PME, behind PLE and posterior slope of prosoma in life (Fig. 4G, H; Caleb and Mathai 2014: figs 4, 6). Ocular region covered with white scales. Dark brown AME covered with white and yellowish hairs in the anterior and posterior portions respectively (Caleb and Mathai 2014: fig. 5). Clypeus enclosed with dense white scales. Chelicerae unidentate, orangish brown. Sternum yellow covered with pale yellow hairs (Fig. 6F). In alcohol-preserved specimen, carapace yellow with faded black patches (Fig. 6E).

Abdomen yellow, oval-shaped, longer, and narrower than prosoma. Dorsum with two lateral blackish stripes extending longitudinally along the length of the abdomen (Fig. 4G, H; Caleb and Mathai 2014: figs 4, 6). Ventrum enclosed with white scales with devoid of any markings in life. Spinnerets pale yellow. Legs glassy greenish yellow.

Epigynum moderately sclerotised. Copulatory openings placed laterally outwards (Caleb and Mathai 2014). CD diverge initially and then bend inward to form a duck-neck-shaped diverging curve leading to CO (Figs 6G, H, 18C, D, Caleb and Mathai 2014: figs 9, 10, 13, 14). Oval spermathecae with head-like structure at the anterior wall and placed closely to each other. FD lanceolate, originating from anterolateral wall of receptacles (Figs 6H, 18D). Posterior epigynal border rather broad.

**Measurements.**TL 4.22, PL 1.91, PW at PLE 1.84, AL 2.30, AW 1.32. Eye field: diameter of AME 0.51, PLE 0.33, ALE 0.26, PME 0.11, PME-PME 1.22, PLE-PLE 0.66, ALE-PME 0.33, ALE-PLE 0.68. Leg I: TR 0.28, FM 2.12, PT 0.92, TB 1.85, MT 1.65, TA 0.73; Leg II: TR 0.27, FM 1.66, PT 0.70, TB 1.42, MT 0.82, TA 0.81; Leg III: TR 0.30, FM 1.81, PT 0.72, TB 1.26, MT 1.20, TA 0.53; Leg IV: TR 0.31, FM 1.80, PT 0.67, TB 1.28, MT 1.53, TA 0.65.

####### Distribution.

India and Sri Lanka (new record).

###### Phintelloides orbisa sp. n.

Taxon classificationAnimaliaAraneaeSalticidae

http://zoobank.org/98293723-FD3F-44CD-BDD7-CB053628FFB2

Figs 12E–H , 13A, B

####### Type material.

**Holotype** 1♀ (IFS_SAL_306), Sri Lanka, North Central Province, Anuradapura District, Mihintale Sanctuary, 123 m, 08°21'10.60"N, 80°30'14.54"E, hand collection, 06-VII-2013, leg. SP Benjamin et al.

####### Etymology.

The species name a noun in apposition, is derived from the Latin *orbis* and refers to the coiled CD.

####### Diagnosis.

This species is distinguishable from *P.alborea*, *P.brunne*, *P.jesudasi*, and *P.flavumi* by having coiled CD (Figs 12G, H, 13A, B) and from *P.flavoviri* by reniform spermathecae, CD with 2–3 coils and absence of sclerotised structures originating from CD.

####### Description.

Female. In ethanol preserved specimens, prosoma orange in colour with black blotches on the ocular region (Fig. 12E). PLE surrounded with black scales. Posterior region of prosoma with devoid of any markings (Fig. 12E). Posterior margin of prosoma slightly truncated. Pale yellow sternum oval in shape.

Abdomen yellow, oval in shape as long as prosoma. Dorsum devoid of any longitudinal stripes, as in other congeners, two pairs of yellowish orange dots at the middle of abdomen (Fig. 12E). Ventrum pale yellow without any markings (Fig. 12F). Spinnerets pale yellow.

Epigynum with poorly sclerotised PEB. Spermathecae comparably large, thick walled and reniform with head-like structure (Figs 12G–H, 13A–B). CD twisted with 2–3 coils, ends in a duck-neck-shaped diverging curve leading to CO. Fertilisation ducts lanceolate, arising from anterolateral wall of receptacles (Figs 12H, 13B).

**Measurements.**TL 5.29, PL 2.28, PW at PLE 1.88, AL 3.35, AW 1.48. Eye field: diameter of AME 0.44, PLE 0.15, ALE 0.25, PME 0.01, PME-PME 1.10, PLE-PLE 1.15, ALE-PME 0.03, ALE-PLE 0.68. Leg I: TR 0.27, FM 1.15, PT 0.78, TB 1.40, MT 0.93, TA 0.33; Leg II: TR 0.27, FM 1.30, PT 0.40, TB 0.77, MT 0.66, TA 0.45; Leg III: TR 0.25, FM 1.22, PT 2.25, TB 0.87, MT 0.93, TA 0.43; Leg IV: TR 0.25, FM 1.00, PT 0.55, TB 1.10, MT 1.32, TA 0.47.

**Male.** Unknown.

###### Chrysilla

Taxon classificationAnimaliaAraneaeSalticidae

Thorell, 1887

####### Type species.

*Chrysillalauta* Thorell, 1887

####### Diagnosis.

Carapace low, twice as long as eye field, gently sloping behind eye field, broader behind PME. Cephalic region slightly broad anteriorly, flat above. Hairy narrowed clypeus. Anterior eyes in a straight line. Chelicerae elongate, directed diagonally forwards, slightly diverging distally with prominent retrolateral tooth. Sternum broadly truncate in front. Legs IV longer than legs III. Abdomen longer and narrower than prosoma. Long dark spinnerets.

Furthermore, *Chrysilla* can be separated from *Phintella* by the bright, metallic colouration of body, narrower and longer abdomen, comparably slender, quite longer and gently bent embolus, elongated oval-shaped apical tegulum, much longer than wide genital bulb, elongated cymbium (Ahmed et al. 2014), single and strong RTA nearly half of the tegulum, copulatory openings separated by ca. one diameter, and pyriform or rounded spermathecae of epigyne (Caleb 2016; Wang and Zhang 2012). For a detailed diagnosis and description see Ahmed et al. (2014), Dyal (1935) and Prószyński and Deeleman-Reinhold (2010).

According to Prószyński (2016)*Chrysilla* may be best recognised by its colouration. Further, Caleb (2016) also claimed that the presence of a colourful body with shiny scales separates *Chrysilla* from *Phintella*. However, as several species of *Phintella* such as *P.vittata* and *P.argentea*, also have a shiny body colouration and as shown in this study over reliance on body colouration could lead to ambiguities.

####### Remarks.

Caleb (2016) suggested that *C.lauta* and *C.volupe* might be conspecific, as he was unable to differentiate them due to the minor differences in their somatic and genital morphology. However, we are able to diagnose *C.lauta* and *C.volupe*, based on our material from Sri Lanka as given below.

###### Chrysilla lauta

Taxon classificationAnimaliaAraneaeSalticidae

Thorell, 1887

Figs 19A–E , 20A, B

####### Material examined.

1♂ (IFS_SAL_699), Sri Lanka, Western Province, Panadura, Mahabellana, along Bolgoda south lake, 9 m, 06°42'48"N, 79°54'09"E, beating, -XII-2008, leg. SP Benjamin and SK Dayananda.

####### Diagnosis.

**Figure 19.**
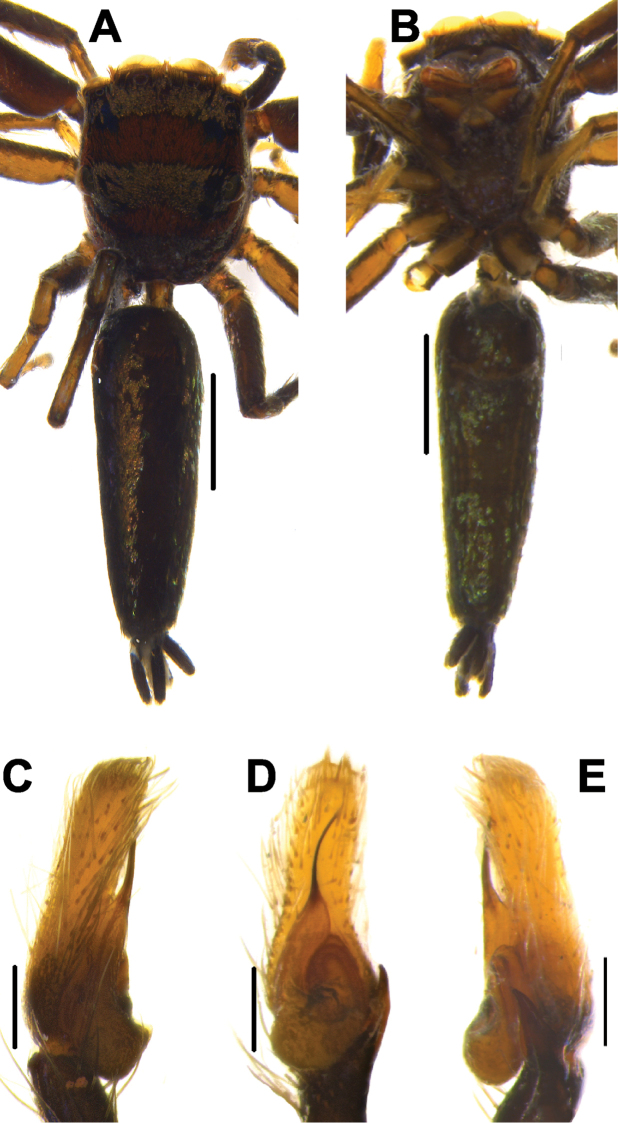
*Chrysillalauta* (**A, B**). Male habitus **A** dorsal view **B** ventral view **C–E** Male palp **C** prolateral view **D** ventral view **E** retrolateral view. Scale bars: 1 mm (**A, B**), 0.2 mm (**C–E**).

**Figure 20.**
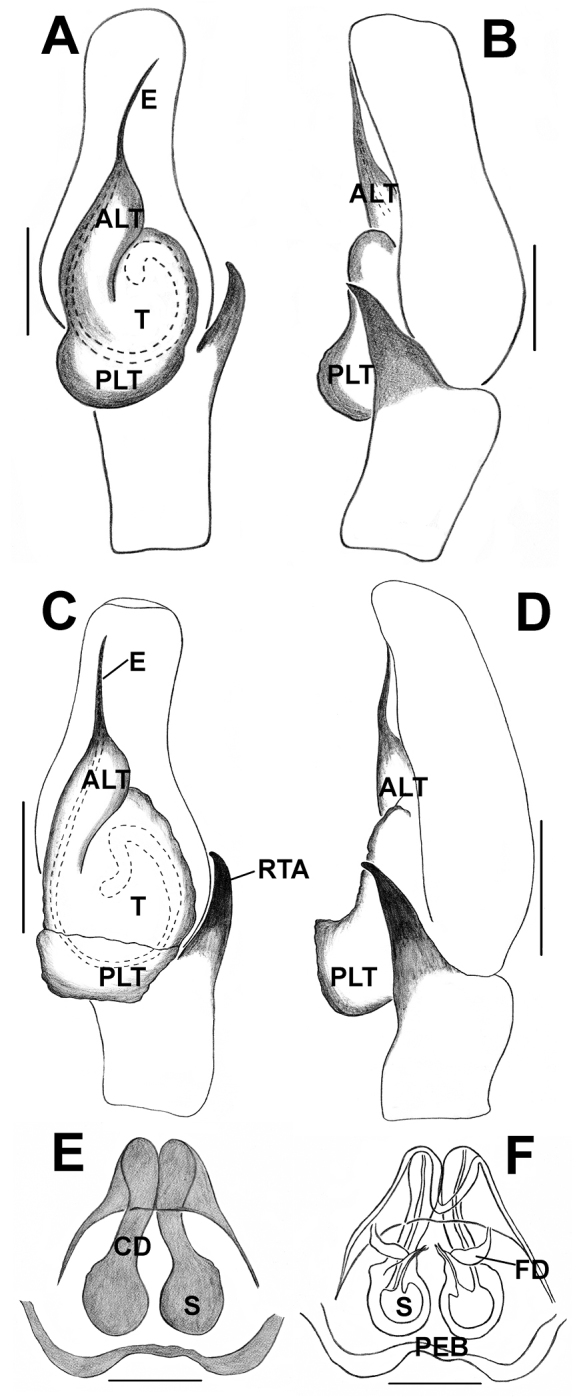
*Chrysillalauta* (**A, B**). **A** Palp, ventral view **B** Palp, retrolateral view. *C.volupe* (**C–F**) **C** Palp, ventral view **D** Palp, retrolateral view **E** Epigynum, ventral view **F** Vulva, dorsal view. Abbreviations: ALT = apical lobe of tegulum; CD = copulatory ducts; E = embolus; FD = fertilisation ducts; PEB = posterior epigynal border; PLT = proximal lobe of tegulum; RTA = retrolateral tibial apophysis; S = spermatheca; T = tegulum. Scale bars: 0.2 mm (**A–B**), 0.1 mm (**C–F**).

This species is closely related to *C.volupe* and *C.deelemani* Prószynski, Deeleman-Reinhold, 2010 in the general form of palpal structure and is distinguishable from them by the abdominal scutum (Fig. 19A, B) and absence of “M”-shaped orange scales on the dorsal abdomen, round shape of PLT, and comparably stouter RTA (Figs 19D, E, 20A, B).

####### Description.

Male. Prosoma arranged with iridescent blue and reddish orange scales (Fig. 19A, B). Edges of prosoma covered with thin line of iridescent blue scales. AME and ALE black in colour, enclosed with reddish orange scales. Clypeus with blackish blue iridescent scales. Reddish brown chelicerae with two promarginal and one retromarginal teeth, sternum oval-shaped covered with iridescent scales. First pair of legs blackish blue, long and larger than other pairs. Other legs beige in colour.

Narrow abdomen tapers to the iridescent blackish blue spinnerets. Dorsum of abdomen covered with iridescent blackish blue scutum decorated with silvery markings (Fig. 19A). Ventrum blackish grey in colour.

Palp metallic blue, except for golden yellow cymbium. Cymbium elongated, narrower at the distal end (Figs 19C–E, 20A, B). Embolus medium sized, bent at the tip. Sperm duct clearly visible at the shoulder of tegulum. Posterior lobe of tegulum is rounded, extended diagonally. Apical portion of bulbus narrow extending beyond the distal end of tegulum (Figs 19D, E, 20A, B). RTA with broad based, with a bulge at the dorsal edge, sharply pointed, bent forward at the tip (Figs 19D, E, 20A, B).

**Measurements.**TL 5.00, PL 2.10, PW at PLE 1.65, AL 2.80, AW 3.08. Eye field: diameter of AME 0.42, PLE 0.31, ALE 0.25, PME 0.09, PME-PME 1.16, PLE-PLE 1.22, ALE-PME 0.41, ALE-PLE 0.51. Leg I: TR 0.32, FM 1.45, PT 0.59, TB 1.24, MT 0.81, TA 0.54; Leg II: TR 0.31, FM 1.23, PT 0.54, TB 0.78, MT 0.65, TA 0.56; Leg III: TR 0.27, FM 1.32, PT 0.42, TB 0.63, MT 0.75, TA 0.54; Leg IV: TR 0.29, FM 1.23, PT 0.34, TB 1.20, MT 1.21, TA 0.66.

**Female.** Unknown.

###### Chrysilla volupe

Taxon classificationAnimaliaAraneaeSalticidae

(Karsch, 1879)

**Figure 21.**
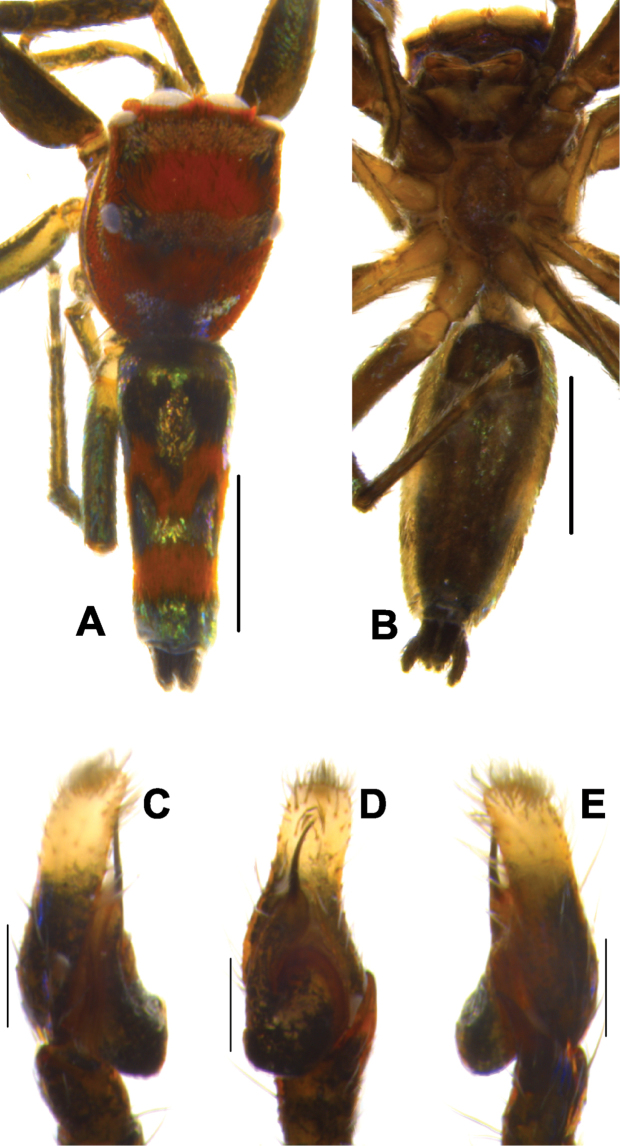
*Chrysillavolupe***A, B** Male habitus **A** dorsal view **B** ventral view **C–E** Male palp **C** prolateral view **D** ventral view **E** retrolateral view. Scale bars: 2 mm (**A, B**), 0.2 mm (**C–E**).

**Figure 22.**
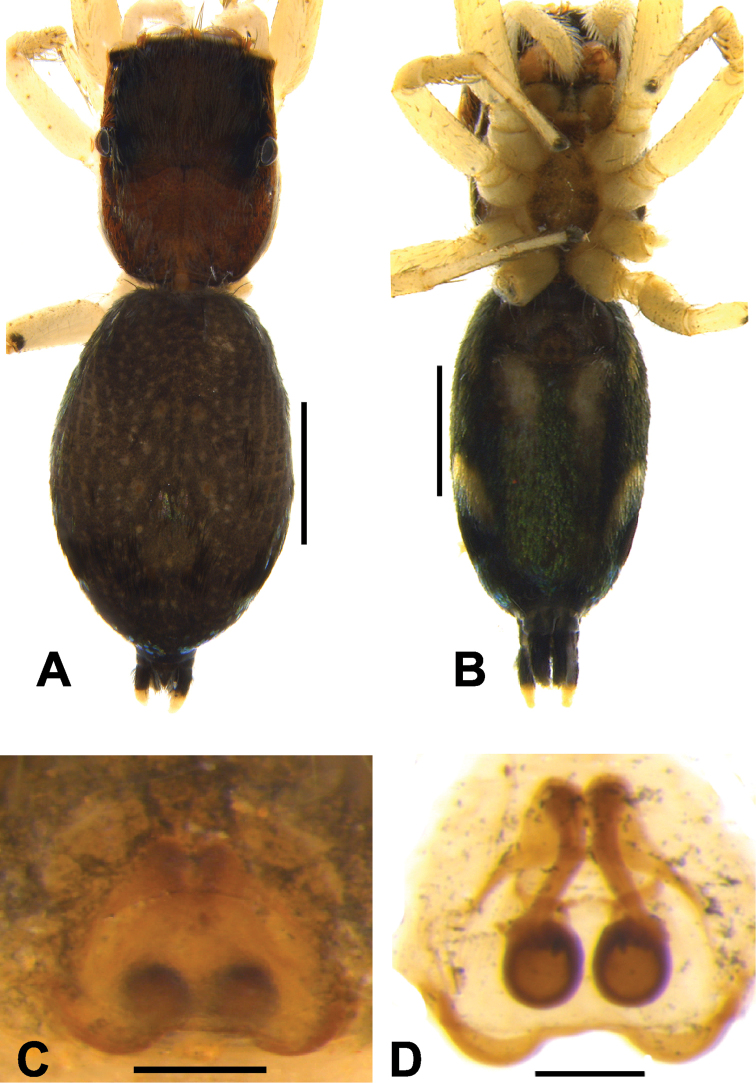
*Chrysillavolupe***A, B** Female habitus **A** dorsal view **B** ventral view **C** Epigynum, ventral view **D** Vulva, dorsal view. Scale bars: 2 mm (**A, B**), 0.1 mm (**C, D**).

Figs 20C–F , 21A–E , 22A–D

####### Material examined.

1♂ (IFS_SAL_239), Sri Lanka, Central Province, Kandy District, Kandy town, hand collection, 10-VI-2015, leg. CI Clayton. 1♂ (IFS_SAL_443), Sri Lanka, Central Province, Kandy District, Ballagola, 467 m, 07°17'12"N, 80°42'48E, 22-VI-2013, leg. SP Benjamin. 3♂, 2♀ (IFS_SAL_633-638), Sri Lanka, North Central Province, Anuradapura District, Mihintale Sanctuary, 123 m, 08°21'10.60"N, 80°30'14.54"E, hand collection, 22-VI-2013, leg. I Sandunika. 1♂ (IFS_SAL_669), Sri Lanka, North Western Province, Kurunagala District, Ethagala FR, 190 m, 07°29'11.23"N, 80°22'21.64"E, hand collection, 1-28-VII-2007, leg. Z Jaleel. 1♂ (IFS_SAL _860), Sri Lanka, Central Province, Matale District, IFS Arboretum, 180 m, 07°51'34"N, 80°40'28"E, 17-VIII-2012, leg. SP Benjamin et al.

####### Diagnosis.

This species is closely related to *C.lauta* and *C.deelemani* in palpal structure and it is distinguishable from *C.lauta* by the absence of abdominal scutum, and rectangular PLT in males and from latter by abdominal metallic colour pattern in both sexes. See also Caleb and Mathai (2014) for a detailed diagnosis.

####### Description.

Male. In live spiders, prosoma covered by iridescent blue and reddish orange scales arranged as alternative bands with thin layer of blue iridescent scales, edged of prosoma cover. AME and ALE black in colour, enclosed with reddish orange scales. Clypeus with iridescent blue scales. Reddish brown chelicerae with two promarginal and one retromarginal teeth, sternum oval and covered with iridescent scales (Caleb and Mathai 2014: figs 12, 15). First pair of legs blackish blue, longer and larger than other pairs. Abdomen tapers to the black spinnerets. Dorsum of abdomen covered with reddish orange, iridescent blue and black patches, orange scales arranged in ‘M’ shape on the abdomen (Fig. 21A; Caleb and Mathai 2014: figs 15, 18). Ventrum blackish grey colour.

Palp metallic bluish black. Cymbium narrower at the distal end than proximal region. Embolus medium sized and slightly curves at the tip (Żabka 1988). Sperm duct is clearly visible at the distal end of tegulum. Proximal lobe of tegulum is partitioned by conspicuous groove (Figs 20C, 21D). Apical portion of bulbus narrow extending beyond the distal end of tegulum. Strong RTA broad at base but narrow and bent forward at the tip (Figs 20C, D, 21D, E).

**Measurements**TL 3.74, PL 1.94, PW at PLE 1.50, AL 1.85, AW 0.75. Eye field: diameter of AME 0.41, PLE 0.24, ALE 0.23, PME 0.07, PME-PME 1.15, PLE-PLE 1.19, ALE-PME 0.20, ALE-PLE 0.48. Leg I: TR 0.27, FM 1.35, PT 0.54, TB 1.21, MT 0.78, TA 0.55; Leg II: TR 0.24, FM 1.10, PT 0.40, TB 0.72, MT 0.61, TA 0.43; Leg III: TR 0.30, FM 1.20, PT 0.41, TB 0.59, MT 0.75, TA 0.53; Leg IV: TR 0.30, FM 1.23, PT 0.30, TB 1.12, MT 1.20, TA 0.62.

**Female.** In ethanol-preserved specimens, prosoma is reddish brown and ocular region is dark, blackish brown in colour (Figs 22A). Narrow and short fovea between PLE. Chelicerae and labium yellowish brown. Sternum oval with black dots. Lateral sides of prosoma covered with thin line of greyish white scales. Posterior margin of prosoma is truncated. Legs yellow in colour and first pair of legs rather broader than others. Femur IV and IV with black blotches. Abdomen much broader than prosoma (Fig. 22A). Dorsum greyish black decorated with dots, ventrum metallic black with metallic pale yellow markings (Fig. 22B). Anterior spinnerets black with yellow tip, posterior and median pairs black.

Epigynum moderately sclerotised. Posterior margin characterised with two lobed scapum (Figs 20E, F, 22C, D). Spermathecae rounded with thick wall. CD broad originates from anterolateral portion of spermathecae and terminates in cap-like structure at anterior margin. Lanceolate fertilisation ducts open in anterolateral wall of receptacles (Figs 20F, 22D).

**Measurements.**TL 3.92, PL 1.95, PW at PLE 1.52, AL 1.95, AW 0.85. Eye field: diameter of AME 0.42, PLE 0.26, ALE 0.25, PME 0.08, PME-PME 1.16, PLE-PLE 1.19, ALE-PME 0.25, ALE-PLE 0.50. Leg I: TR 0.27, FM 1.37, PT 0.54, TB 1.23, MT 0.81, TA 0.61; Leg II: TR 0.27, FM 1.20, PT 0.51, TB 0.74, MT 0.62, TA 0.51; Leg III: TR 0.32, FM 1.24, PT 0.44, TB 0.61, MT 0.75, TA 0.61; Leg IV: TR 0.30, FM 1.33, PT 0.35, TB 1.22, MT 1.31, TA 0.73.

####### Distribution.

Sri Lanka, India, Bhutan.

###### Genus Proszynskia gen. n.

Taxon classificationAnimaliaAraneaeSalticidae

http://zoobank.org/AC2B0EDC-19A6-4CC6-BECB-5656515297D6

####### Type species.

*Viciriadiatreta* Simon, 1902.

####### Etymology.

The name honours Jerzy Prószyński, one of the most influential salticidologists, who redescribed many type specimens and greatly contributed to our knowledge of jumping spider biodiversity.

####### Monophyly and phylogenetic placement.

**Figure 23.**
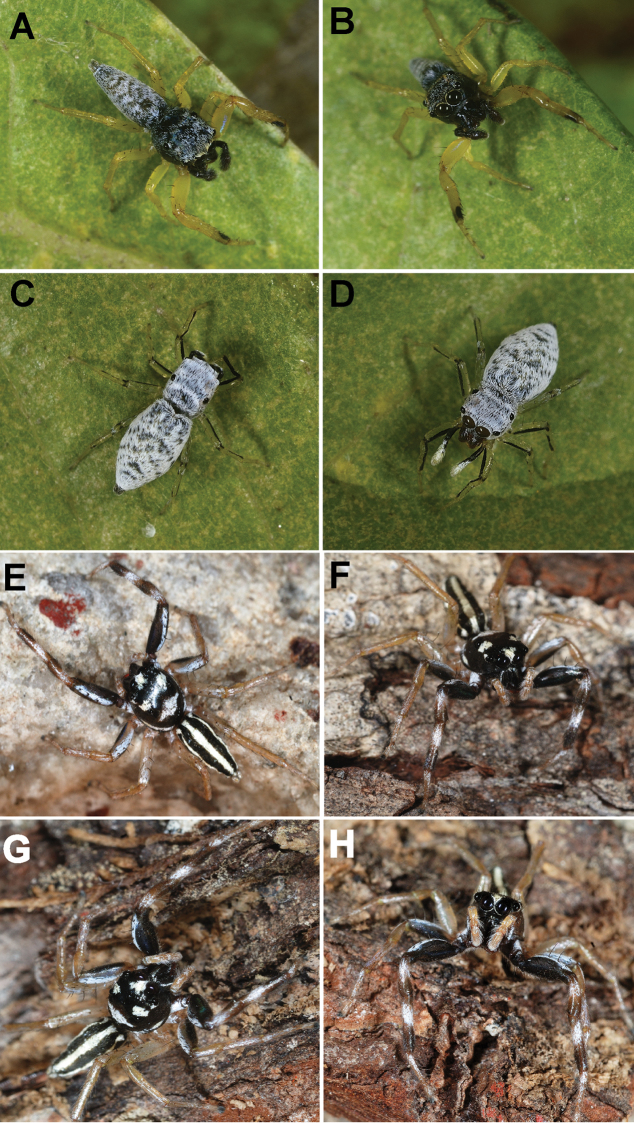
*Phintellajaleeli* (**A–D**) from Ethagala FR**A, B** Male in life **C, D** Female in life and Male of *Proszynskiadiatreta* (**E–H**) from Batticaloa.

**Figure 24.**
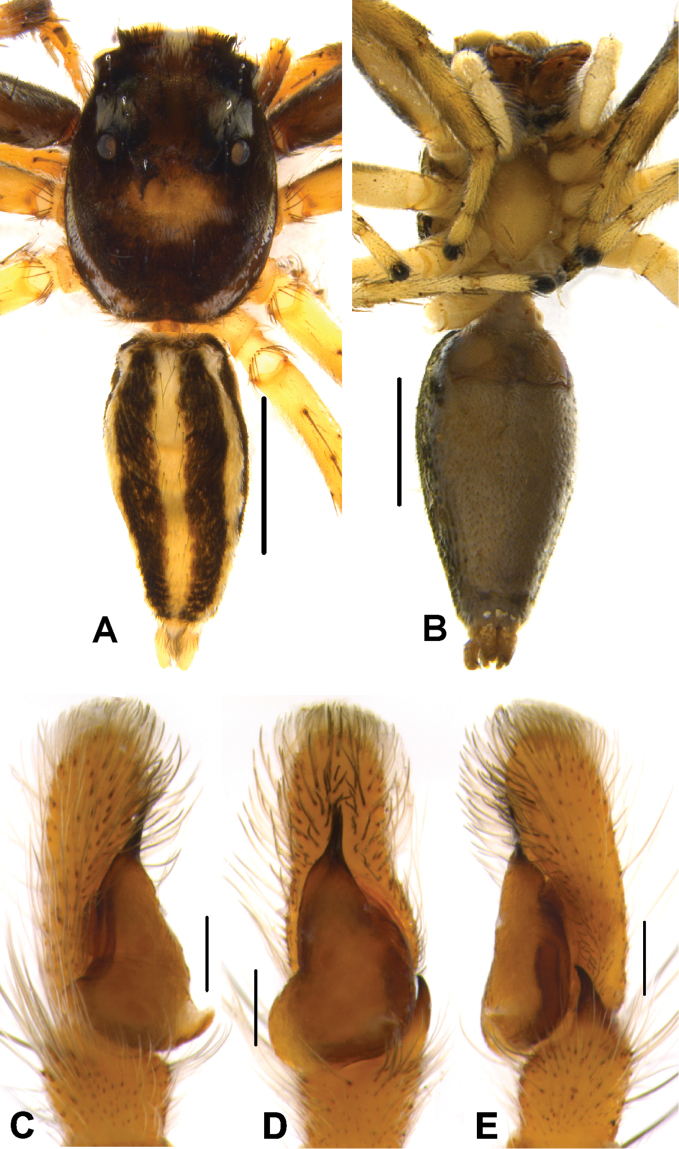
*Proszynskiadiatreta***A, B** Male habitus **A** dorsal view **B** ventral view **C–E** Male palp **C** prolateral view **D** ventral view **E** retrolateral view. Scale bars: 2 mm (**A, B**), 0.2 mm (**C–E**).

**Figure 25.**
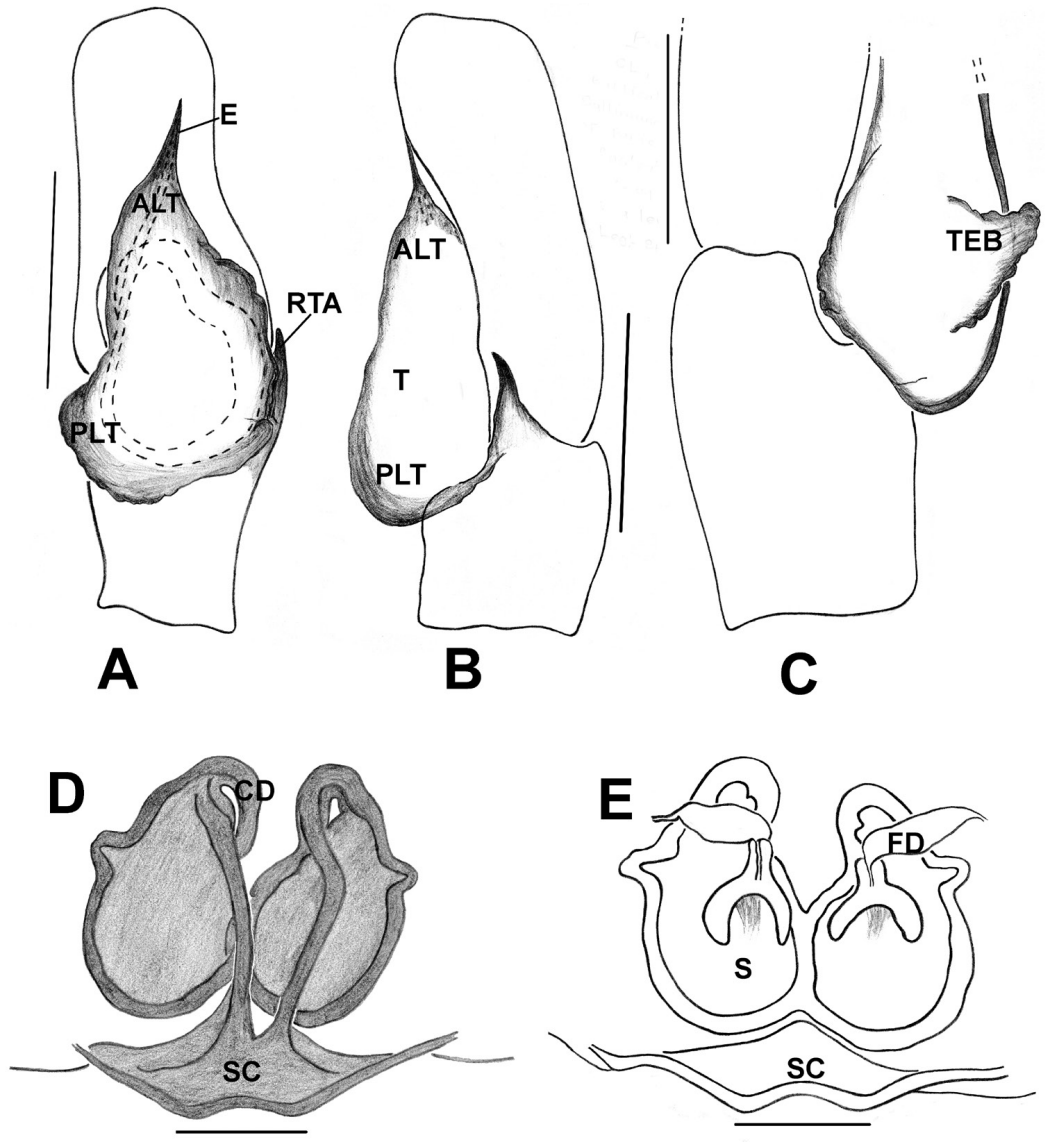
*Proszynskiadiatreta***A–C** Male palp, **A** ventral view **B** retrolateral view **C** prolateral view **D** Epigynum, ventral view **E** Vulva, dorsal view. Abbreviations: ALT = apical lobe of tegulum; CD = copulatory ducts; E = embolus; FD = fertilisation ducts; PLT = proximal lobe of tegulum; RTA = retrolateral tibial apophysis; S = spermatheca; SC = scapum; T = tegulum; TEB = tegular bump. Scale bars: 0.2 mm (**A–C**), 0.1 mm (**C–E**).

**Figure 26.**
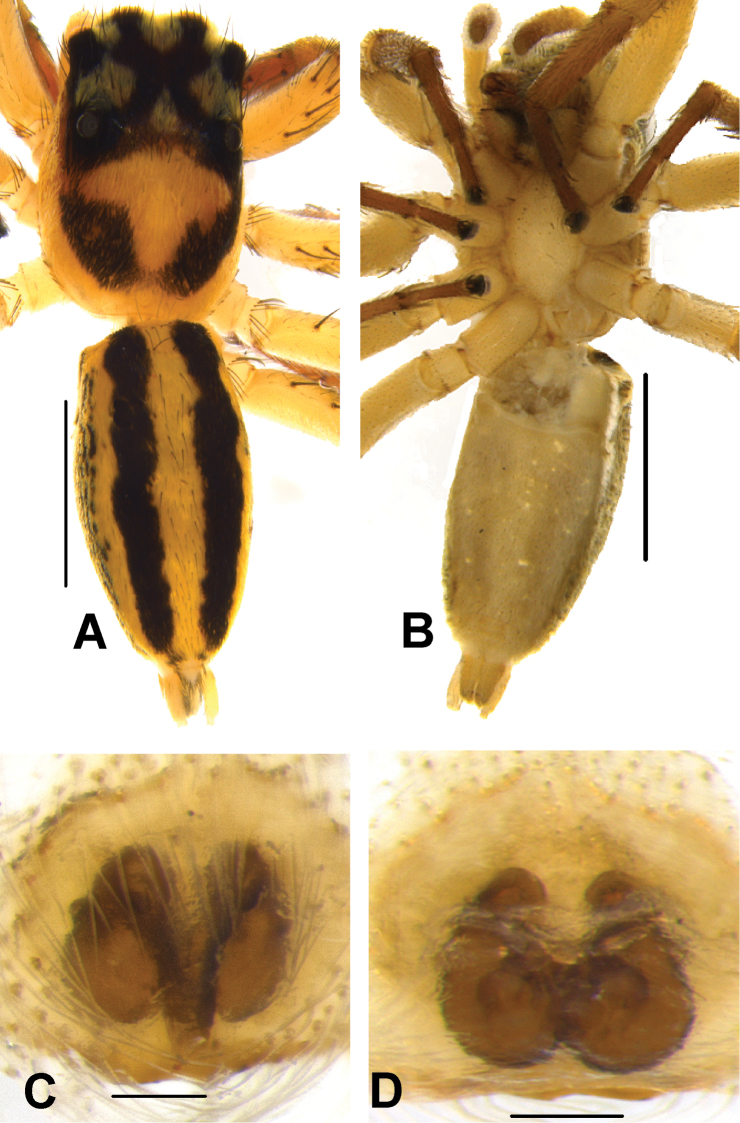
*Proszynskiadiatreta***A, B** Female habitus **A** dorsal view **B** ventral view **C** Epigynum, ventral view **D** Vulva, dorsal view. Scale bars: 2 mm (**A, B**), 0.1 mm (**C–D**).

Monophyly of *Proszynskia* is recovered in all molecular trees and the morphological tree (Figs 1–3) is supported by the following unambiguous putative synapomorphies: pale yellow median abdominal band bordered with two black bands (Fig. 23E–G), more/less straight embolus (Figs 24D, 25A), large, pyriform spermathecae, mid-wall origin of FD (Figs 25D, E, 26C, D).

####### Diagnosis.

This genus can readily be recognised from the closely related genus *Phintelloides* by pale yellow, median longitudinal band bordered with blackish stripes of dorsal abdomen, short and straight embolus, absence of LP, well-developed PLT, narrowed distal part of the tegulum, mid-wall origin of FD and absence of DDC in females. Other differences are the pattern of abdominal dorsal markings and copulatory organ’s morphology including short, robust, and comparably shorter embolus and comparably short RTA in males, absence of curved CD, presence of large and highly sclerotised spermathecae and the presence of SC in females.

####### Description.

Large spiders (7–9mm). Prosoma longer than wide with pale yellow patches on the ocular field; white diamond-shaped mark behind eye field; white belts on lateral prosoma; leg I dark brown and robust in males; tibiae and metatarsi of first two legs with four and two pairs of ventral spines respectively; abdomen with pale yellow longitudinal median band; bordered by blackish brown lateral bands and ventrum uniform pale colour; short and straight embolus; apical portion of bulbus without lamellar process; well-developed proximal lobe of bulbus prolaterally; medium sized RTA with bent tip. Position of CO uncertain; CD short; large, pyriform-shaped spermathecae; FD originating from mid-wall of the receptacles; PEB with partially developed scapum.

####### Composition.

This genus encompasses two known species: *Proszynskiaanusuae* (Tikader & Biswas, 1981) comb. n. and *P.diatreta* (Simon, 1902) comb. n.

####### Remarks.

*Proszynskiadiatreta* was recovered as a separate lineage in all our analyses (Figs 1, 2; Suppl. material 1–3: Figs S1–S3). However, its placement within trees differed. Many more species of the lineage may remain to be discovered in the South Asian region. *Proszynskiaanusuae* comb. n. (formerly *Marpissaanusuae* Tikader & Biswas, 1981: 97, text figs 18–20) is distinct from other *Marpissa* of the subtribe Marpissina Simon, 1901 in habitus as well as copulatory organ’s morphology. Morphologically, *P.anusuae* resembles *P.diatreta* and is thus transferred here.

####### Distribution.

India and Sri Lanka.

###### Proszynskia diatreta

Taxon classificationAnimaliaAraneaeSalticidae

(Simon, 1902) comb. n.

Figs 23E–H , 24A–E , 25A–E , 26A–D

Viciria diatreta Simon, 1902: 366; Prószyński 1984: 433, figs 42–43; Caleb and Mathai 2014: 65, figs 38–46.
Phintella diatreta (Simon, 1902): Caleb 2016: 274.

####### Remarks.

This species was recently transferred to *Phintella* by Caleb (2016). However, it differs from most *Phintella* by general appearance, body size, as well as palpal structure including shape of PLT, narrowed distal part of the tegulum, comparably straight embolus, pyriform spermathecae, and origin of FD through funnel-like structures on the mid-dorsal wall of receptacles. Further, our molecular data of single and combined gene analysis corroborates its placement outside of *Phintella*.

####### Material examined.

1♂ (IFS_SAL_520), Sri Lanka, Eastern province, Batticaloa District, Sallimunai, 4km North of Panichchankerni, Sea level, 08°06'37"N, 81°27'20"E, 07-09-VIII-2010, leg. SP Benjamin and S Batuwita. 1♀ (IFS_SAL_539), Northern Province, Vavuniya District, Poonthottam, Home gardens, 98 m, 08°46'12.95"N, 80°30'32.81"E, hand collection, 27.X.2015, leg. K Nilani. 1♂ (IFS_SAL_861), same locality and collection data, 18-VII-2016. 4♂, 1♀ (IFS_SAL_1011- 1015), same locality and collection data, 13-I-2017.

####### Diagnosis.

This species is distinguishable from *Proszynskiaanusuae* comb. n. by the sclerotised structures from spermathecae and funnel-like unusual structures connecting spermathecae and FD (Figs 25D, E, 26C, D).

####### Description.

Male. Prosoma black, decorated with greenish yellow patches on the ocular region, a patch between the AME, two lateral patches placed between PLE and PME (Fig. 23E–G; Caleb and Mathai 2014: figs 38, 40, 41). Ocular field slightly elevated. Behind PLE, there is a white, prominent diamond-shaped mark. (Figs 23E, G). Lateral sides of prosoma decorated with white belts (Figs 23E–G; Caleb and Mathai 2014: figs 38, 40). Clypeus covered with black scales (Fig. 23H). Chelicerae black with two promarginal and one retromarginal teeth, labium black in colour. Sternum oval, greyish yellow. Posterior margin of prosoma rather steep and slightly truncated. Leg I dark blackish yellow and robust with black femur and white hairs dorsally, ventral portion of femur II covered with black scales, others blackish yellow.

Abdomen longer and slightly narrower than prosoma, tapering posteriorly. Dorsum with narrow pale yellow median band, surrounded by two black bands bordering with pale yellow lateral bands extending longitudinally from anterior to posterior end (Fig. 23E–G; Caleb and Mathai 2014: fig. 38). Ventrum blackish grey in life (Caleb and Mathai 2014: fig. 39) and yellowish brown in preserved specimens (Fig. 24B). Spinnerets greenish yellow.

Brownish yellow palp. Cymbium narrows at the distal region. Embolus straight and robust, immovable on rather broad apical portion of tegulum (Figs 24D, 25A). LP absent. Bulbus longer than wide. Spermatophore loop clearly visible at the shoulder of tegulum. Small bulge at retrolateral portion of bulbus. Tegulum with prolaterally expanded proximal lobe. RTA broader at the base, narrower and bent forward at the tip (Figs 24D–E, 25A, B).

**Measurements.**TL 7.00, PL 3.15, PW at PLE 2.80, AL 3.90, AW 2.00. Eye field: diameter of AME 0.53, PLE 0.28, ALE 0.30, PME 0.05, PME-PME 1.32, PLE-PLE 1.20, ALE-PME 0.38, ALE-PLE 0.78. Leg I: TR 0.35, FM 1.92, PT 0.88, TB 1.73, MT 1.18, TA 0.75; Leg II: TR 0.30, FM 1.71, PT 1.00, TB 1.22, MT 1.12, TA 0.68; Leg III: TR 0.28, FM 1.73, PT 0.72, TB 1.10, MT 1.10, TA 0.74; Leg IV: TR 0.30, FM 1.93, PT 0.80, TB 1.32, MT 1.21, TA 0.82.

**Female.** Almost all somatic characters similar to male except shape of the mark behind PLE and comparably less strong first pair of legs (Fig. 26A, B). Epigynum highly sclerotised. Spermathecae large and pear-shaped (Figs 25D, E, 26C, D). Position of copulatory openings remains unclear. Copulatory ducts short and bent inward dorsally. FD lanceolate, opening in funnel-like structures in the mid-dorsal wall of receptacles (Figs 25E, 26D). Posterior margin characterised with moderately sclerotised scapum from which two sclerotised structures running parallel and fused with anterior wall of spermathecae (Figs 25D, 26C).

**Measurements.**TL 8.45, PL 3.75, PW at PLE 3.30, AL 4.60, AW 2.70. Eye field: diameter of AME 0.55, PLE 0.28, ALE 0.35, PME 0.05, PME-PME 1.35, PLE-PLE 1.20, ALE-PME 0.38, ALE-PLE 0.78. Leg I: TR 0.36, FM 1.90, PT 0.80, TB 1.63, MT 1.18, TA 0.76; Leg II: TR 0.30, FM 1.75, PT 1.00, TB 1.32, MT 1.15, TA 0.66; Leg III: TR 0.26, FM 1.75, PT 0.70, TB 1.15, MT 1.15, TA 0.70; Leg IV: TR 0.35, FM 1.89, PT 0.85, TB 1.30, MT 1.20, TA 0.80.

####### Distribution.

India and Sri Lanka (new record).

###### Genus Phintella

Taxon classificationAnimaliaAraneaeSalticidae

Strand, in Bösenberg & Strand, 1906

####### Type species.

*Telamoniabifurcilinea* Bösenberg & Strand, 1906.

####### Diagnosis.

*Phintella* are usually small to medium (3–6 mm in length). Some species are covered with metallic iridescent scales. They are characterised by relatively high cephalothorax with distinctive posterior slope, unidentate chelicerae, tegulum with lobe and bump about 90°clockwise from the base of the embolus, embolus sets apically, usually short, pointed or furcate, palpal tibia with one or more apophyses. Female genitalia simple with rounded spermathecae in most species, insemination ducts of different length, usually not twisted (Żabka 2012).

###### Phintella argentea sp. n.

Taxon classificationAnimaliaAraneaeSalticidae

http://zoobank.org/F65B2846-F58A-4402-91B2-D1C1B63AAD61

**Figure 27.**
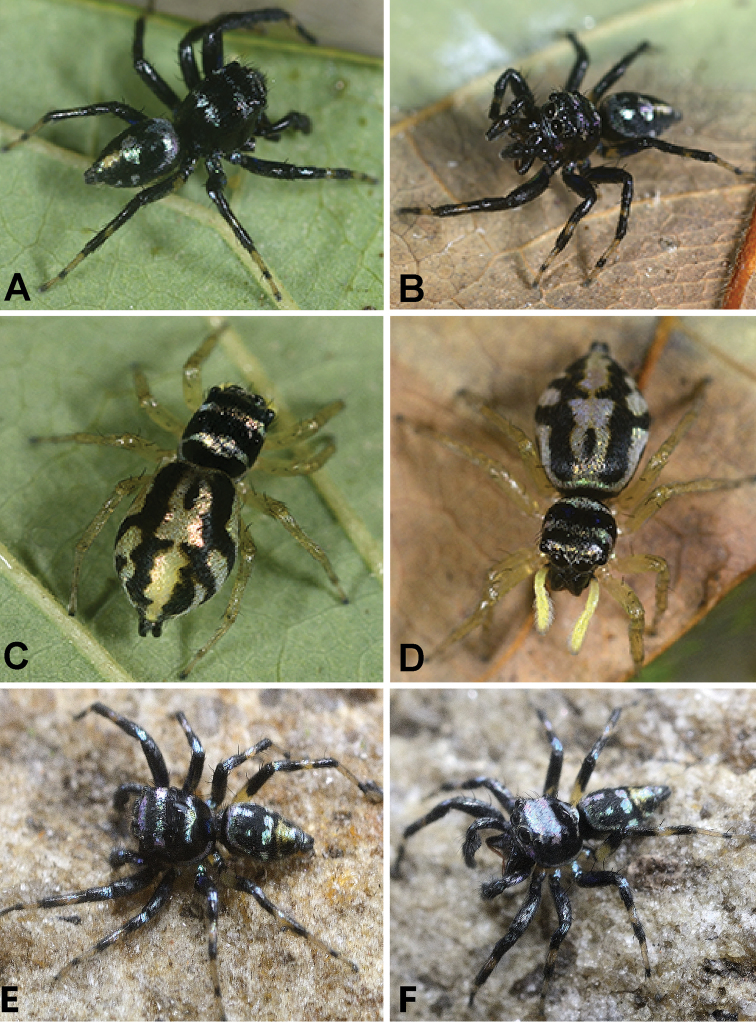
*Phintellaargentea***A, B** Male from Loolecondera estate **C–D** Female from Loolecondera estate **E–F** Male from Hakgala SNR.

**Figure 28.**
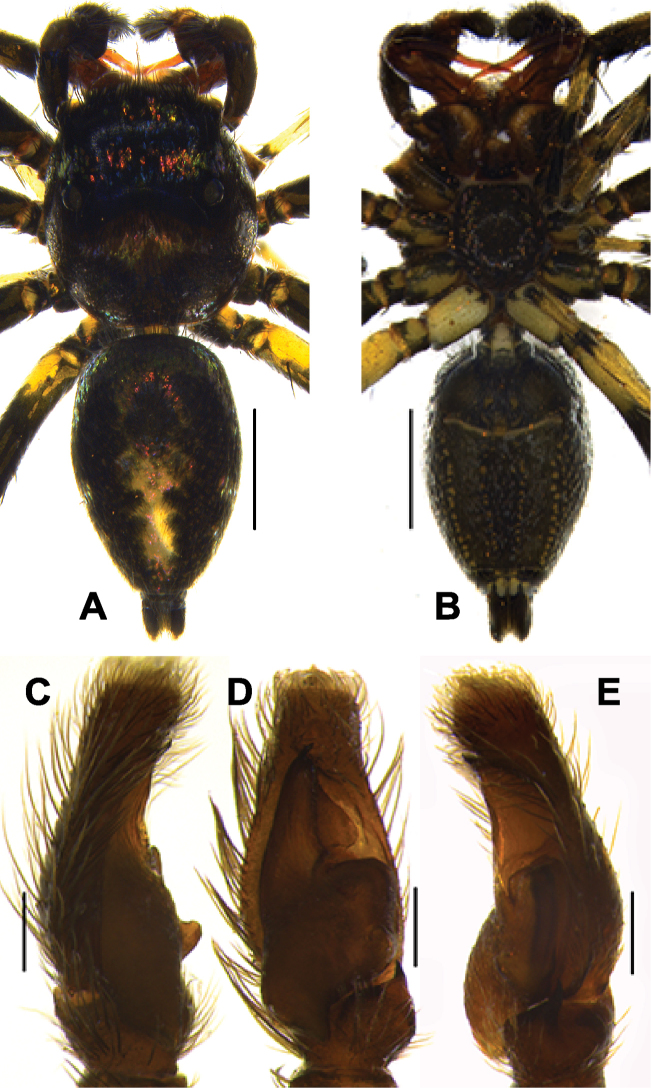
*Phintellaargentea***A, B** Male habitus **A** dorsal view **B** ventral view **C–E** Male palp **C** prolateral view **D** ventral view **E** retrolateral view. Scale bars: 1 mm (**A, B**), 0.2 mm (**C–E**).

**Figure 29.**
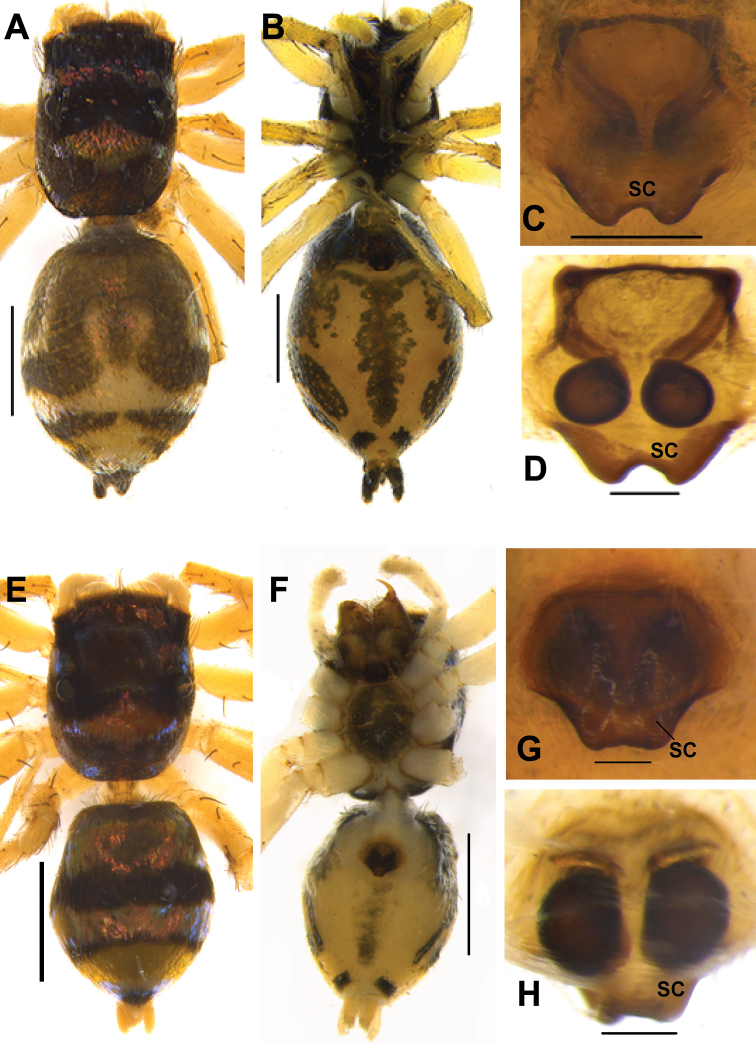
*Phintellaargentea* (**A–D**) **A, B** Female habitus **A** dorsal view **B** ventral view **C, D** Epigynum **C** ventral view **D** dorsal view; *Phintellavittata* (**E–H**) **E–F** Female habitus **E** dorsal view **F** ventral view **G–H** Epigynum **G** ventral view **H** dorsal view. Scale bars: 1 mm (**A–B, E–F**), 0.1 mm (**C–D, G–H**).

**Figure 30.**
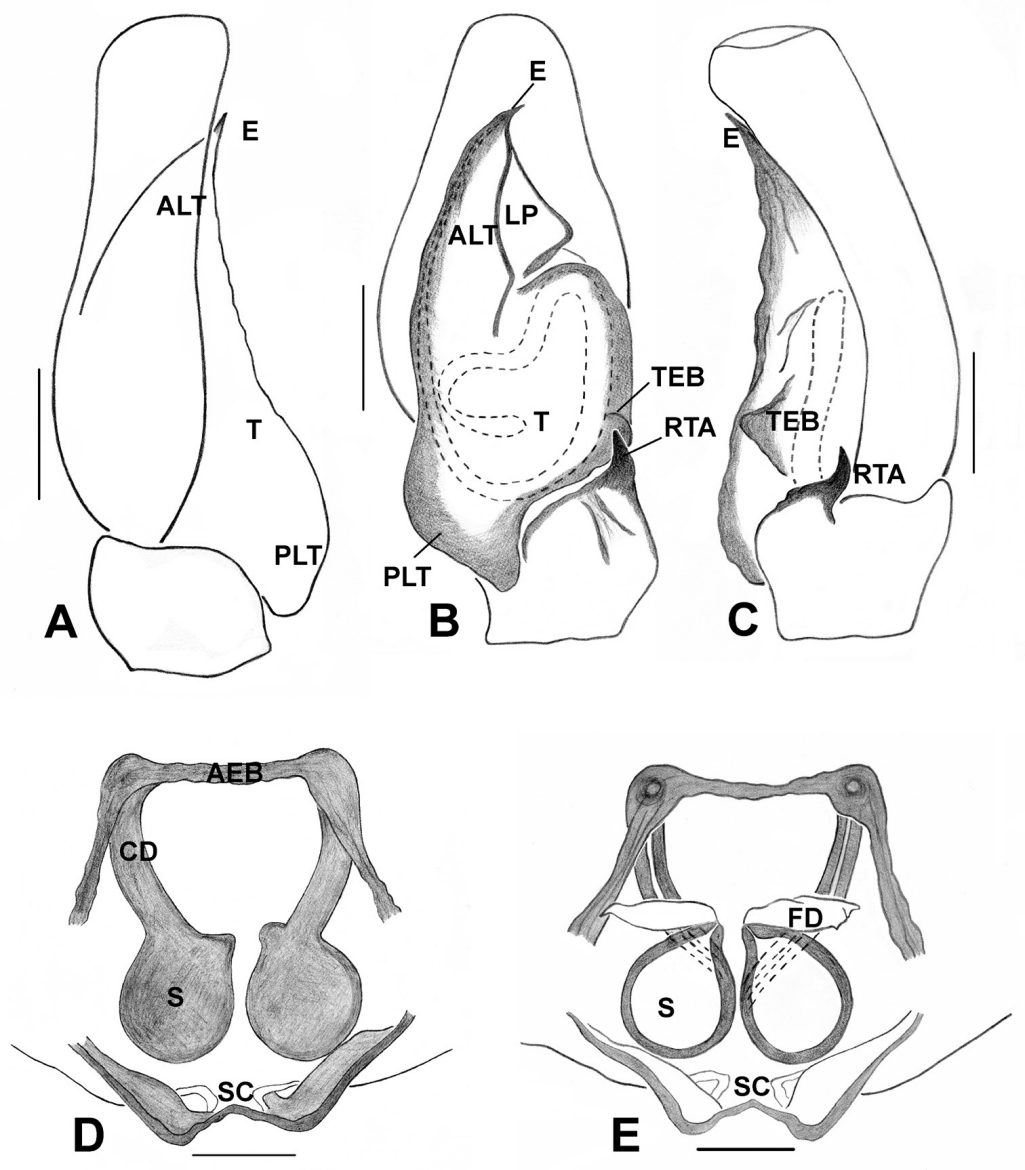
*Phintellaargentea***A–C** Male palp **A** prolateral view **B** ventral view **C** retrolateral view **D** Epigynum, ventral view **E** Vulva, dorsal view. Abbreviations: AEB = anterior epigynal border; ALT = apical lobe of tegulum; CD = copulatory ducts; E = embolus; FD = fertilisation ducts; LP = lamellar process; PLT = proximal lobe of tegulum; RTA = retrolateral tibial apophysis; S = spermatheca; T = tegulum; TEB = tegular bump. Scale bars: 0.2 mm (**A–C**), 0.1 mm (**D–E**).

Figs 27A–F , 28A–E , 29A–D , 30A–E

####### Type material.

**Holotype** ♂ (IFS_SAL_085): Sri Lanka, Central Province, Nuwara Eliya District, Agarapatana, Bopattalawa FR, 1665 m, 06°50'36"N, 80°40'40"E, hand collection, 18–21-II-2007, leg. SP Benjamin and Z Jaleel. **Paratype.** ♀ (IFS_SAL_086): same locality and collection data as in holotype.

####### Other material examined.

2♀ (IFS_SAL_087-088), Sri Lanka, Central Province, Nuwara Eliya District, Agarapatana, Bopattalawa FR, 1665 m, 06°50'36"N, 80°40'40"E, hand collection, 18–21-II-2007, leg. SP Benjamin and Z Jaleel. 1♀, 1♂ (IFS_SAL_191-192), same locality and collection data. 1♀ (IFS_SAL_452), Kandy District, Deltoa, Loolcondera FR, 1480 m, 07°08'45"N, 80°41'53"E, hand collection, 11-V-2010, leg. S Batuwita, N Athukorala. 1♂, ♀ (IFS_SAL_857-858), same locality and collection data, 22-VI-2016, leg. I Sandunika. 2♀ (IFS_SAL_457-458), Nuwara Eliya District, Horton plains NP, 2141 m, 06°47'54"N, 80°48'51"E, hand collection, 20–21-II-2007, leg. SP Benjamin and Z Jaleel. 2♂ (IFS_SAL _893-894), Upcot, 1850 m, 06°46’N, 80°36’E, beating, 03-X-2016, leg. K Nilani and I Sandunika.

####### Etymology.

This species name a noun in apposition, is derived from the Latin *argenteus*, and refers to the presence of characteristic silvery markings on the prosoma and abdomen of spiders of this species.

####### Diagnosis.

The species is closely related to *Phintellaaccentifera* (Simon, 1901), *P.aequipeiformis* Zabka, 1985, *P.suavis* (Simon, 1885) and *P.vittata* (C. L. Koch, 1846) in palpal morphology. However, it is distinguishable from them by the markings of dorsal abdomen in both sexes (Fig. 27A–F), more elongated ALT and LP (Figs 28C–E, 30B, C) in males and much longer copulatory ducts that diverge above spermathecae and deep recurved SC border in females (Figs 29C, D, 30D, E).

####### Description.

Male. In life, rounded prosoma covered with black shiny scales. Transverse silvery marking on the middle of the eye field (Fig. 27A, B, E, F). Ocular region slightly elevated. Clypeus enclosed with silvery black glistening setae (Fig. 27B, F). AME surrounded with silvery scales. Chelicerae glossy black, blackish brown fangs. Transverse large diamond-shaped silvery blotch behind eye field (Fig. 27A, E). Rounded sternum, blackish grey, rounded furrow at the middle in preserved specimens (Fig. 28B). Silvery patches on each lateral sides of prosoma (Figs 27A, E, F). Posterior margin of prosoma steeper, slightly truncated at the middle. Glossy brownish black legs with greyish yellow banding pattern, leg I somewhat robust.

Abdomen slightly narrower than prosoma, tapering posteriorly. Glossy black dorsum decorated with silvery band at the anterior margin, silvery blotches at the middle and lateral sides and golden yellow mark near spinnerets at the posterior end (Fig. 27A, B, E, F). Ventrum blackish grey, greyish yellow parallel dotted lines from epigastric furrow to spinnerets, in preserved specimens (Fig. 28B). Spinnerets shiny black.

Brownish black and strongly sclerotised palp. Short cymbium, narrow distal region. Embolus short, somewhat stout, immovable above the apical portion of tegulum, extending beyond the level of the distal end of tegulum (Figs 28D, 30B). Comparably narrower, longer apical portion of tegulum (Figs 28D, E, 30B, C). Large, conspicuous, triangular flap on side of embolus (Figs 28D, E, 30B). Bulbus tapering, sperm duct comparably broader at distal end of tegulum, narrower at the apical portion of tegulum. Small hump at retrolateral portion of bulbus (Figs 28C, D, 30B, C). Proximal lobe of tegulum with well-developed posterior lobe at the prolateral portion. Palpal tibia short, dorsally bent RTA (Figs 28D, E, 30B, C).

**Measurements.**TL 4.65, PL 1.90, PW at PLE 1.50, AL 2.2, AW 1.5. Eye field: diameter of AME 0.40, PLE 0.1, ALE 0.22, PME 0.01, PME-PME 1.24, PLE-PLE 1.08, ALE-PME 0.32, ALE-PLE 0.62. Leg I: TR 0.30, FM 1.46, PT 0.97, TB 1.32, MT 1.08, TA 0.51; Leg II: TR 0.27, FM 1.16, PT 0.54, TB 0.84, MT 0.70, TA 0.46; Leg III: TR 0.24, FM 1.24, PT 0.51, TB 0.94, MT 0.97, TA 0.46; Leg IV: TR 0.27, FM 1.22, PT 0.54, TB 1.05, MT 1.16, TA 0.46.

**Female.** Similar to male except greyish yellow legs, leg I not robust, broader abdomen than prosoma, covered with golden yellow gleaming scales, dorsal pattern of abdomen as in Fig. 27C, Ventrum pale yellow, dark green medians, lateral blotches in preserved specimens, pale yellow palp (Fig. 29B).

Epigynum moderately sclerotised, fully developed scapum extending beyond the line of epigastric furrow (Figs 29C, D, 30D–E). CO clearly distinguishable, located inside of strongly sclerotised hood-like structure at the anterior margin. Rounded spermathecae, thick walled (Figs 30D, E, 32D). Copulatory canals comparably longer. FD lanceolate, originating from the anterior wall of receptacles (Fig. 30E).

**Measurements.**TL 4.80, PL 2.00, PW at PLE 1.45, AL 2.65, AW 1.75. Eye field: diameter of AME 0.40, PLE 1.34, ALE 0.27, PME 0.01, PME-PME 1.19, PLE-PLE 1.11, ALE-PME 0.35, ALE-PLE 0.62. Leg I: TR 0.24, FM 1.32, PT 0.86, TB 1.27, MT 0.97, TA 0.43; Leg II: TR 0.24, FM 1.16, PT 0.49, TB 0.81, MT 0.68, TA 0.43; Leg III: TR 0.24, FM 1.16, PT 0.46, TB 0.92, MT 0.94, TA 0.46; Leg IV: TR 0.24, FM 1.16, PT 0.54, TB 1.03, MT 1.11, TA 0.46.

###### Phintella jaleeli sp. n.

Taxon classificationAnimaliaAraneaeSalticidae

http://zoobank.org/49F84074-4112-4B4E-9EA7-96B4DFD4805E

**Figure 31.**
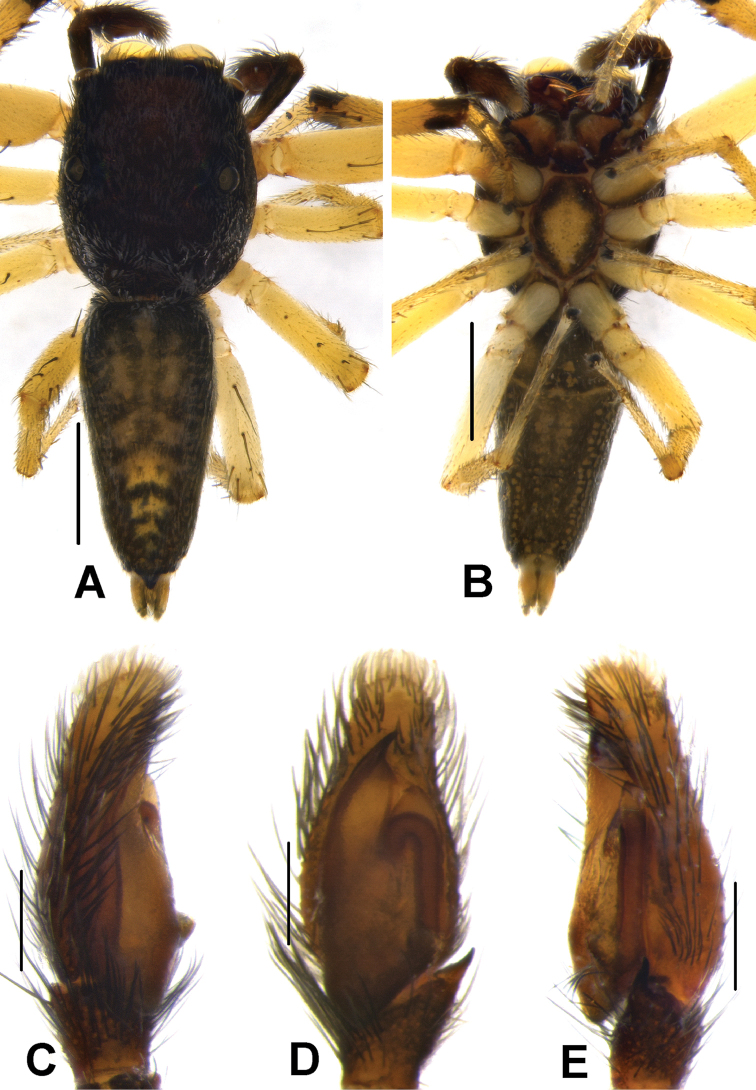
*Phintellajaleeli***A, B** Male habitus **A** dorsal view **B** ventral view **C–E** Male palp **C** prolateral view **D** ventral view **E** retrolateral view. Scale bars: 1 mm (**A, B**), 0.2 mm (**C–E**).

**Figure 32.**
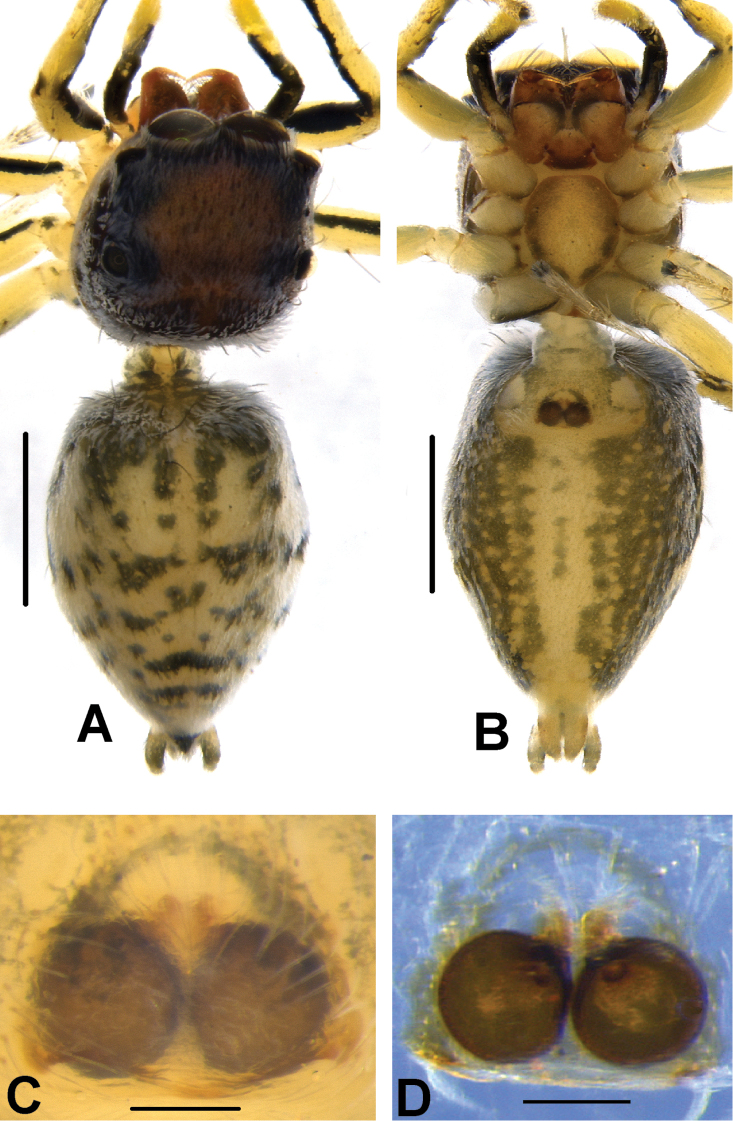
*Phintellajaleeli***A, B** Female habitus **A** dorsal view **B** ventral view **C** Epigynum, ventral view **D** Vulva, dorsal view. Scale bars: 2 mm (**A, B**), 0.1 mm (**C–D**).

**Figure 33.**
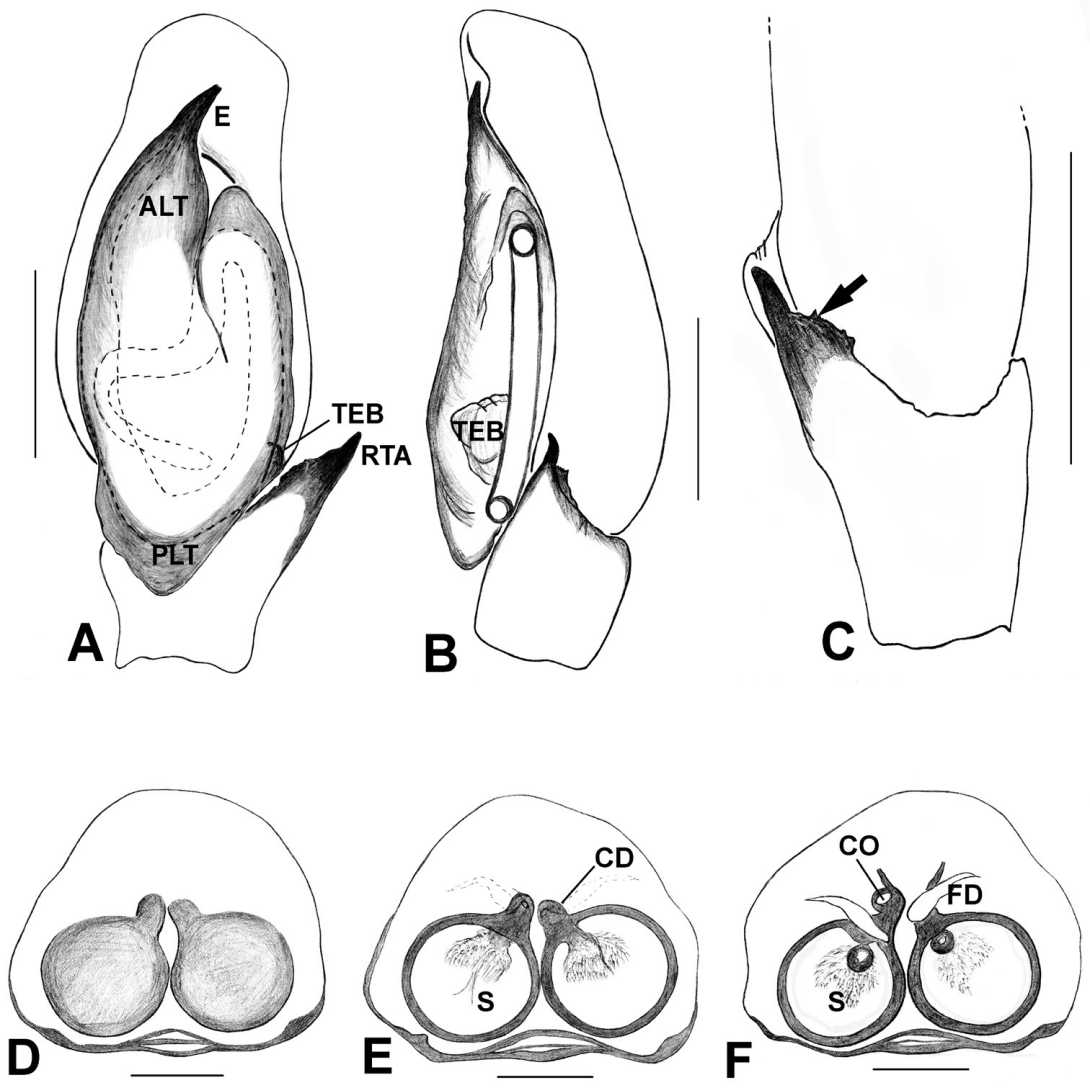
*Phintellajaleeli***A–C** Male palp **A** ventral view **B** retrolateral view **C** retro-ventral view **D–E** Epigynum, ventral view **F** Vulva, dorsal view. Abbreviations: ALT = apical lobe of tegulum; CD = copulatory ducts; CO = copulatory opening; E = embolus; FD = fertilisation ducts; PLT = proximal lobe of tegulum; RTA = retrolateral tibial apophysis; S = spermatheca; TEB = tegular bump. Scale bars: 0.2 mm (**A–C**), 0.1 mm (**D–F**). Arrow points to minute teeth on RTA.

Figs 23A–D , 31A–E , 32A–D , 33A–F

####### Type material.

**Holotype** ♂ (IFS_SAL_407): Sri Lanka, North Western Province, Kurunagala District, Ethagala FR, 190 m, 07°29'11.23"N, 80°22'21.64"E, hand collection, 15-VII-2007, leg. Z Jaleel. **Paratype.** ♀ (IFS_SAL_408): Same locality and collection data as in holotype.

####### Other material examined.

2♂ (IFS_SAL_409–410): Same locality and collection data as in type materials. 4♂, 1♀ (IFS_SAL_079–083): Same locality and collection data, 1–28-II-2007. 1♂ (IFS_SAL_164), same locality and collection data, 190 m, 07°29'11.23"N, 80°22'21.64"E, beating, 08-IV-2015, leg. SP Benjamin et al. 1♂ (IFS_SAL_265), 07°28'17"N, 80°30"E, 24-XI-2009, same locality and collection data, SP Benjamin and S Batuwita. 1♂ (IFS_SAL_271), same locality and collection data 07°29'11.23"N, 80°22'21.64"E, beating, 08-IV-2015, N Athukorala. 6♂, 6♀ (IFS_SAL_178–189), Nikaravatiya, hand collection, 1–3-XI-2007, leg. Z Jaleel. 2♀ (IFS_SAL_200–201), same locality and collection data, 11-II-2007. 3♂, 3♀ (IFS_SAL_213–218), same locality and collection data, 21-I-2008. 2♂, 4♀ (IFS_SAL_387–392), same locality and collection data, 1–3-11-2007. 1♂ 4♂, 1♀ (IFS_SAL_790–792), 252 m, 07°29'11.23"N, 80°22'21.64"E, hand collection, 07-VI-2016, leg. N Athukorala and K Nilani. 1♂ (IFS_SAL_102), Eastern province, Ampara District, Gal Oya NP, 80 m, 07°13'22"N, 81°31'56"E, 10-II-2010, leg. SP Benjamin and S Batuwita. 1♂ (IFS_SAL_262), Central province, Matale District, IFS Arboretum, 180 m, 07°51'34"N, 80°40'28"E, litter, 19-VI-2014, N Athukorala et al. 2♂, 1♀ (IFS_SAL_438–440), same locality, 188 m, 07°51'34"N, 80°40'28"E, 17-VIII-2012, leg. SP Benjamin et al. 1♀ (IFS_SAL_927): Western Province, Gampaha District, Pilikuttuwa FR, 69 m, 07°03'52.4"N, 80°03'04"E, beating, 28-IX-2016, leg. K Nilani.

####### Etymology.

This name is a patronym for the collector Z Jaleel, who collected many specimens of this species and other spiders described by us here and elsewhere.

####### Diagnosis.

The species is similar to *P.abnormis* (Bösenberg & Strand, 1906) in copulatory organ morphology; however, it is distinguishable by RTA with basal minute teeth and absence of LP in males (Figs 31D–E, 33A–C) and globular spermathecae, very short CD and absence of SC (Figs 32D, 33D–F).

####### Description.

Male. In life, prosoma blackish brown, somewhat oval, broad in shape, sparsely covered with greyish white hairs (Fig. 23A). Clypeus with black scales. Chelicerae glossy black with reddish brown fangs. Oval sternum, yellowish brown in middle, dark green at the periphery, edges reddish brown colour in ethanol preserved specimens (Fig. 31B). Posterior margin of prosoma steep, slightly truncated. Leg I greenish yellow, robust, prominent black patches on lateral sides of tibia I, others greenish yellow (Fig. 23A, B).

Abdomen longer and narrower than prosoma, tapering posteriorly. Dorsum densely covered with greyish white hairs in life (Fig. 23A), blackish green, pale yellow chevrons in preserved specimens (Fig. 31A). Anal tubercle blackish brown, more prominent than in other congeners (Fig. 31A). Ventrum blackish green with parallel dot lines from epigastric furrow to spinnerets in preserved specimens, spinnerets greenish yellow (Fig. 31B).

Blackish brown palp. Short cymbium narrows at the distal region. Embolus short and robust immovable on rather broad apical portion of tegulum (Figs 31D, 33A). Lamellar process absent. Bulbus more elongated than broad. Sperm duct clearly visible at the shoulder of tegulum. Small protuberance at retrolateral portion of bulbus (Figs 31C, E, 33A–C). Proximal lobe of tegulum with small posterior lobe. Broader base of RTA with minute teeth, narrower and slightly bent forward at the tip (Figs 31D–E, 33A–C).

**Measurements.**TL 3.90, PL 1.80, PW at PLE 1.45, AL 2.10, AW 1.05. Eye field: diameter of AME 0.51, PLE 0.19, ALE 0.22, PME 0.01, PME-PME 1.24, PLE-PLE 1.08, ALE-PME 0.32, ALE-PLE 0.62. Leg I: TR 0.30, FM 1.22, PT 0.43, TB 1.08, MT 0.54, TA 0.38; Leg II: TR 0.24, FM 1.10, PT 0.24, TB 0.76, MT 0.38, TA 0.30; Leg III: TR 0.32, FM 1.13, PT 0.30, TB 0.86, MT 0.49, TA 0.35; Leg IV: TR 0.32, FM 1.16, PT 0.32, TB 0.92, MT 0.49, TA 0.38.

**Female.** In life, prosoma blackish brown, broad and densely covered with greyish white hairs (Figs 23C, D). Clypeus covered with greyish white scales. Chelicerae glossy blackish brown with brownish yellow fangs. Oval sternum, yellowish brown in middle and edges covered with dark green and reddish brown patches in ethanol preserved specimens (Fig. 32B). Posterior margin of prosoma steep and slightly truncated. Legs glassy greenish yellow with black longitudinal stripes on the lateral sides (Figs 23C, D), leg I not robust as in males. Black palp with greyish white end.

Abdomen longer and slightly broader than prosoma. Dorsum densely covered with greyish white hairs in life (Figs 23C, D), blackish green with pale yellow chevrons in preserved specimens (Fig. 32A). Anal tubercle blackish brown and more prominent than other congeners. Ventrum blackish green with greyish yellow middle band and parallel dot lines from epigastric furrow to spinnerets in preserved specimens (Fig. 32B). Spinnerets greenish yellow.

Epigynum with large, globe-like and highly sclerotised spermathecae with thin wall. Scapum absent (Figs 35C, 36D), copulatory openings small in front of receptacles. Copulatory ducts too short and broad (Figs 32C, D, 33D–F). FD lanceolate, opening into the anterior wall of receptacles (Fig. 33F).

**Measurements.**TL 4.00, PL 1.90, PW at PLE 1.45, AL 2.15, AW 1.20. Eye field: diameter of AME 0.51, PLE 0.21, ALE 0.24, PME 0.01, PME-PME 1.10, PLE-PLE 1.08, ALE-PME 0.35, ALE-PLE 0.65. Leg I: TR 0.24, FM 0.97, PT 0.24, TB 0.73, MT 0.38, TA 0.27; Leg II: TR 0.24, FM 1.03, PT 0.22, TB 0.76, MT 0.41, TA 0.32; Leg III: TR 0.32, FM 1.16, PT 0.35, TB 0.92, MT 0.54, TA 0.40; Leg IV: TR 0.35, FM 1.19, PT 0.35, TB 0.94, MT 0.51, TA 0.40.

###### Phintella vittata

Taxon classificationAnimaliaAraneaeSalticidae

CL Koch, 1846

**Figure 34.**
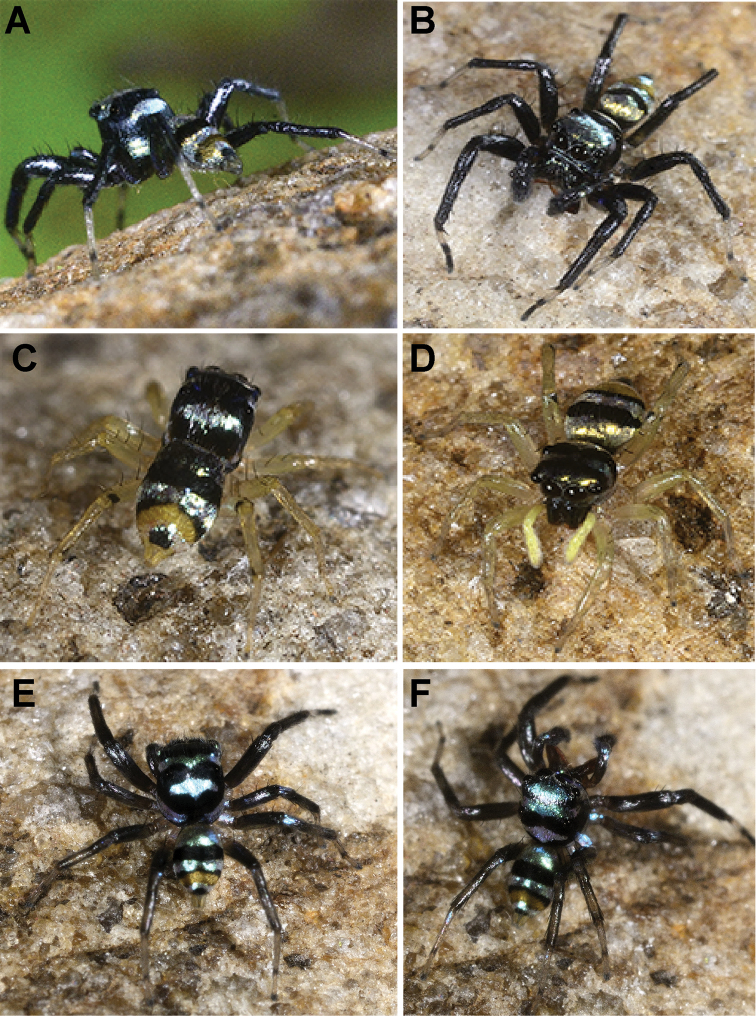
*Phintellavittata***A, B** Male from Hiyare, Kombala-Kottawa FR**C, D** Female from Singaraja FR, Kudawa **E, F** Male from Bowatenna.

**Figure 35.**
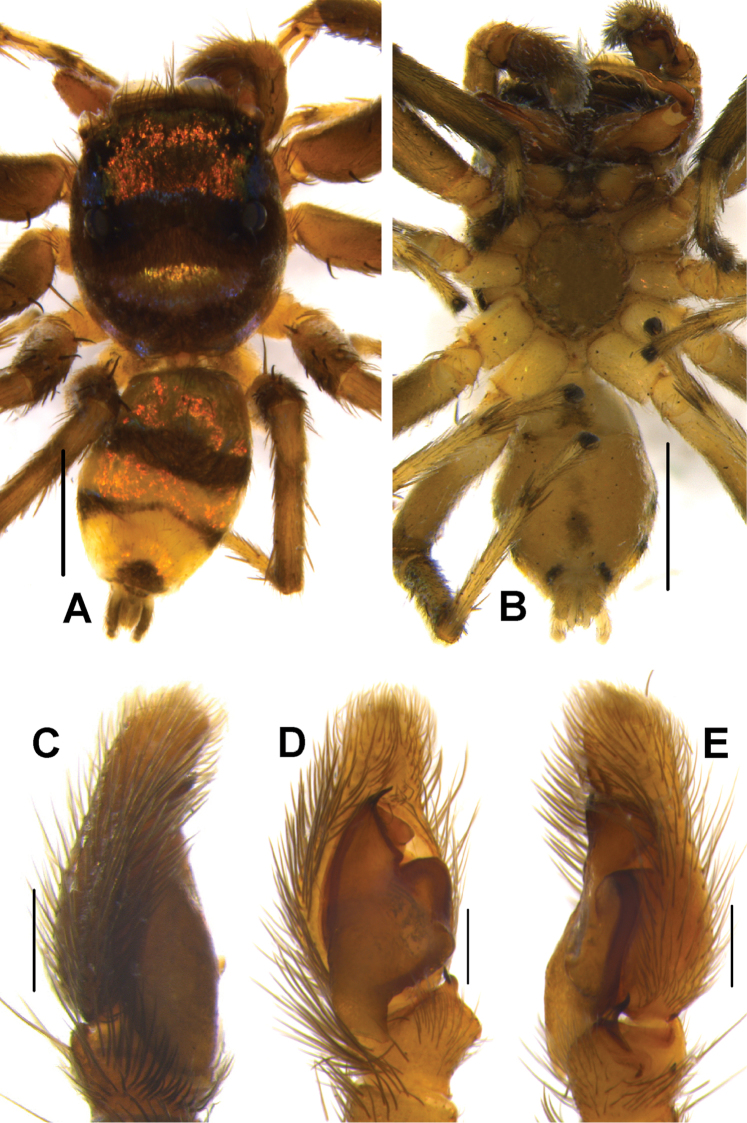
*Phintellavittata***A, B** Male habitus **A** dorsal view **B** ventral view **C–E** Male palp **C** prolateral view **D** ventral view **E** retrolateral view. Scale bars: 1 mm (**A, B**), 0.2 mm (**C–E**).

**Figure 36.**
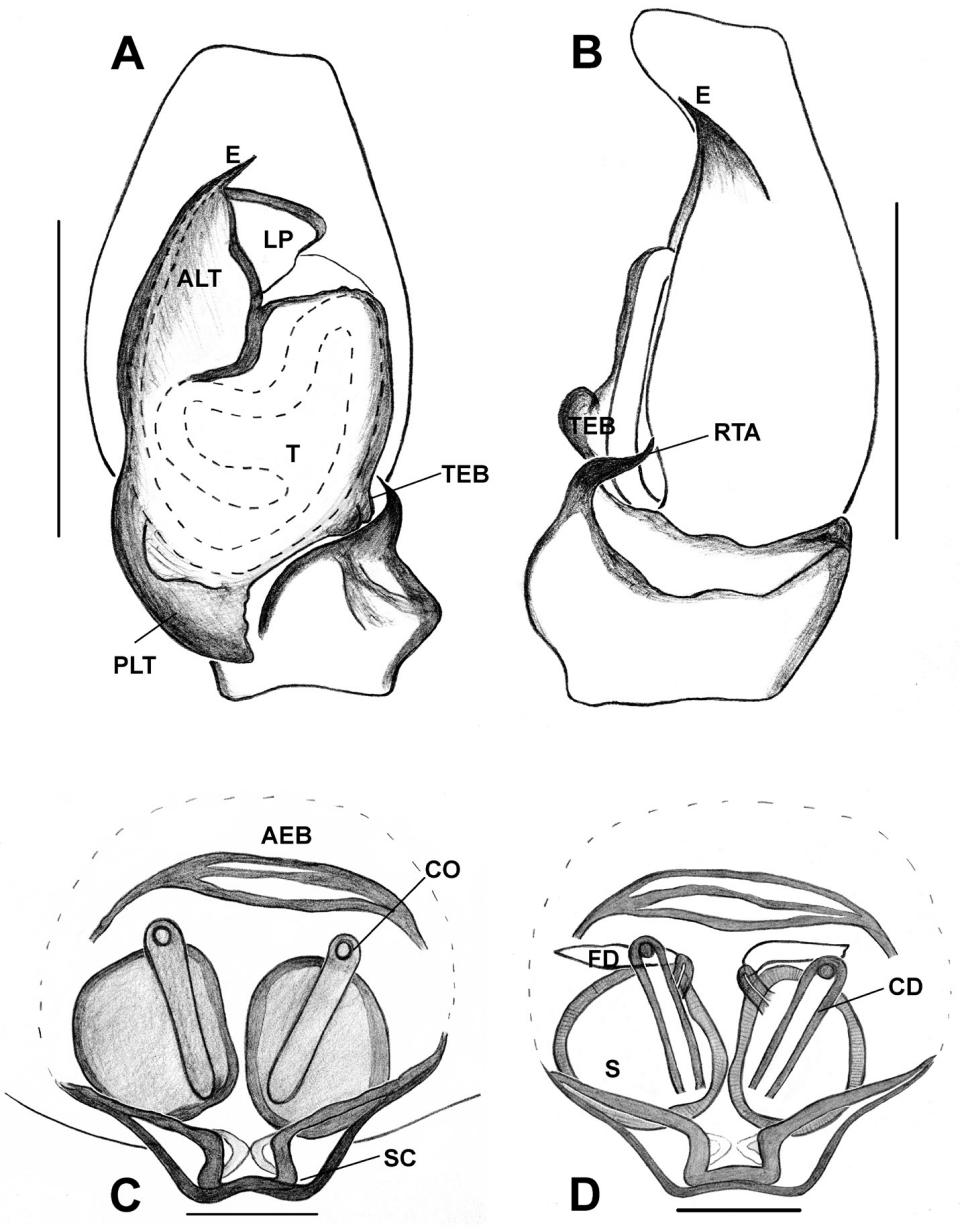
*Phintellavittata***A, B** Male palp **A** ventral view **B** retrolateral view **C** Epigynum, ventral view **D** Vulva, dorsal view. Abbreviations: AEB = anterior epigynal border; ALT = apical lobe of tegulum; CD = copulatory ducts; CO = copulatory opening; E = embolus; FD = fertilisation ducts; LP = lamellar process; PLT = proximal lobe of tegulum; RTA = retrolateral tibial apophysis; S = spermatheca; T = tegulum; TEB = tegular bump. Scale bars: 0.2 mm (**A–B**), 0.1 mm (**C–D**).

Figs 29E–H , 34A–F , 35A–E , 36A–D

####### Material examined.

2♀ (IFS_SAL_196-198), Sri Lanka, North Western Province, Kurunagala District, Ethagala FR, 190 m, 07°29'11.23"N, 80°22'21.64"E, hand collection, 11-II-2007, Z Jaleel. 1♀ (IFS_SAL_138-139), same locality and collection data, 20-V-2007. 1♂, 2♀ (IFS_SAL_670-672), same locality and collection data, 1-28-VII-2007. 1♂ (IFS_SAL_227), same locality and collection data, 17-XI-2007. 1♀ (IFS_SAL_290), same locality and data, 09-XII-2007. 2♀ (IFS_SAL_138–139), same locality and collection data, 07°29'11.23"N, 80°22'21.64"E, 300m, 1–28-II-2008, leg. Z Jaleel. 2♂, 1♀ (IFS_SAL_210-212), same locality and data, 21-I-2008. 1♂, 1♀ (IFS_SAL_384-385), Nikaravatiya, hand collection 1-3-11-2007, Z Jaleel. 1♂ (IFS_SAL_507), same locality and data. 2♀ (IFS_SAL _176-177), Central Province, Nawalapitiya, March, 2008, leg. Z Jaleel. 1♂ (IFS_SAL_604), Matale District, Bowatenna, Reservoir area, secondary shrub along road to bund, 252 m, 07°39'37"N, 80°41'18"E, beating, 10-II-2016, leg. K Nilani. 1♂, 1♀ (IFS_SAL_877-878), Gannoruwa forest, 575 m, 07°17'16"N, 80°35'47"E, beating, 30-VIII-2016, leg. K Nilani and I Sandunika. 1♂, 1♀ (IFS_SAL_240-241), Eastern Province, Ampara Disrtict, Samangala, 112 m, 07°24'38.27"N, 81°34'52.38"E, beating, 19-V-2015, leg. N Athukorala. 5♂, 5♀ (IFS_SAL_701-710), Western Province, Kalutara District, Panadura, Mahabellana, along Bolgoda south lake, 9 m, 06°42'48"N, 79°54'09"E, beating, VII-2008, leg. SP Benjamin, SK Dayananda. 1♀, 1♂ (IFS_SAL _461-462), Kaluthara Disrtict, Paragoda-Bulathsinnhala, 169 m, 06°38'23"N, 80°09'55"E, 07-VIII-2012. 3♂, 2♀ (IFS_SAL_915-919), Gampaha District, Pilikuttuwa FR, 69 m, 07°03'52.4"N, 80°03'04"E, beating, 28-IX-2016, leg. K Nilani and I Sandunika. 1♂, ♀ (IFS_SAL_749-750), Southern Province, Galle District, Hiyare, Kombala-Kottawa FR, 252 m, 06°03'53"N, 80°18'05"E, beating, 24-26-V-2016, leg. K Nilani and I Sandunika. 1♀ (IFS_SAL_816): North Central Province, Anuradapura District, Mihintale Sanctuary, 123 m, 08°21'10.60"N, 80°30'14.54"E, hand collection, 14-VI-2016, leg. K Nilani and I Sandunika.

####### Diagnosis.

The species is closely related to *Phintellaaccentifera*, *P.aequipeiformis*, *P.argentea*, and *P.suavis* in palpal morphology. However, it is distinguishable by the transverse metallic bands on the abdomen in both sexes (Figs 34A–F), comparably smaller and broader ALT, dorsally curved RTA (Figs 35DE, 36A, B) in males and oval spermathecae and moderately recurved SC border in females (Figs 29H, 36C, D).

####### Description.

Male. Small spiders (less than 5.0 mm). In life, prosoma oval and black with transverse blue metallic band on the middle of the eye field (Figs 34A, B, E, F). Surroundings of eyes black-grey with sparse grey-brown setae (Prószyński 1992; Roy et al. 2016; Żabka 1985). Clypeus covered with blackish grey gleaming setae. Chelicerae glossy black with blackish brown fangs. Transverse large diamond-shaped metallic blue blotch behind eye field (Fig. 34A, E, F). Rounded sternum, brownish grey in ethanol preserved specimens (Fig. 35B). Two silvery belts on each lateral sides of prosoma (Fig. 34A, E). Posterior margin of prosoma steep, slightly truncated. Glossy brownish black legs except greyish yellow metatarsus and leg I somewhat robust.

Abdomen slightly shorter and narrower than prosoma, tapering posteriorly. Dorsum decorated with transverse blue and black metallic lustrous banding pattern, single transverse golden yellow at the posterior region (Figs 34A, E, F). Ventrum blackish grey with devoid of markings (Fig. 35B). Spinnerets pale yellow.

Brownish black palp. Short cymbium narrows at distal region. Embolus short, somewhat stout immovable, on rather triangular apical portion of tegulum, extending beyond the level of the distal end of tegulum (Figs 35D–E, 36A–B). Large triangular flap on side of embolus (lamellar process) conspicuous (Figs 35D, 36A). Bulbus elongated. Sperm duct comparably broader at the distal end of tegulum, narrower at the apical portion of tegulum. Small hump at retrolateral portion of bulbus. Proximal lobe of tegulum with well-developed posterior lobe at the prolateral side. Palpal tibia short, two, unequal sized lateral apophysis (Figs 35D, E, 36A, B), a small tooth on the posterior (dorsal) surface of tibia (Peng et al. 1993; Prószyński 1992; Żabka 1985).

**Measurements.**TL 3.40, PL 1.80, PW at PLE 1.24, AL 1.44, AW 1.10. Eye field: diameter of AME 0.45, PLE 0.16, ALE 0.20, PME 0.01, PME-PME 1.22, PLE-PLE 1.00, ALE-PME 0.30, ALE-PLE 0.60. Leg I: TR 0.25, FM 0.625, PT 0.37, TB 0.88, MT 0.63, TA 0.37; Leg II: TR 0.22, FM 0.75, PT 0.37, TB 1.00, MT 0.625, TA 0.50; Leg III: TR 0.18, FM 1.00, PT 0.37, TB 0.63, MT 0.87, TA 0.63; Leg IV: TR 0.20, FM 1.00, PT 0.38, TB 0.88, MT 0.80, TA 0.62.

**Female.** As in male except for greyish yellow legs and distal end of femur IV and tibia IV with black patches (Figs 34C, D). Epigynum strongly sclerotised with well-developed scapum, extending beyond the line of epigastric furrow at the posterior margin (Figs 29G, H, 36C, D). Copulatory openings clearly visible. Large and oval spermathecae with strongly sclerotised thick wall (Figs 29H, 36C, D). Copulatory canals externally in the shape of letter “V” (Żabka 1985). FD lanceolate, opening into the anterior wall of receptacles (Figs 29H, 36D).

**Measurements.**TL 3.65, PL 1.60, PW at PLE 1.56, AL 2.00, AW 1.65. Eye field: diameter of AME 0.42, PLE 0.10, ALE 0.20, PME 0.01, PME-PME 1.26, PLE-PLE 1.00, ALE-PME 0.35, ALE-PLE 0.60. Leg I: TR 0.17, FM 0.76, PT 0.38, TB 0.63, MT 0.45, TA 0.31; Leg II: TR 0.17, FM 1.00, PT 0.30, TB 0.63, MT 0.30, TA 0.50; Leg III: TR 0.24, FM 1.10, PT 0.38, TB 0.67, MT 0.70, TA 0.35; Leg IV: TR 0.25, FM 1.00, PT 0.35, TB 0.87, MT 0.58, TA 0.50.

## Discussion

We provide evidence that the specimens collected by us in Sri Lanka are members of three distinct evolutionary lineages. The monophyly of *Phintella* and *Chrysilla* has never been tested. They never have been clearly defined in phylogenetic terms. Most species of *Phintella* were originally placed in *Chrysilla*, *Telamonia*, *Icius*, or *Jotus*. Many species of both genera are apparently misplaced (Prószyński and Deeleman-Reinhold 2010). We have probably included all known species of both genera from Sri Lanka in addition to existing sequences of *Phintella* from NCBI in our molecular analysis. Our molecular as well as morphological phylogeny suggested that both genera are not closely related to each other as previously assumed and lack clear-cut generic boundaries. This may have led to our initial misidentification of the collected material. *Chrysilla* differs morphologically from *Phintella* by the more elongated slender abdomen, slender, curved and medium sized embolus, comparably longer and stronger single RTA, and smaller spermathecae.

*Chrysilla* is more closely related to *Siler* than to *Phintella* (Figs 1–3). Genitalia of *Chrysilla* and *Phintella* differ considerably. The monophyly of *Chrysilla* is supported by a group of unambiguous synapomorphies: horizontal metallic blue and reddish orange rows of prosoma, more elongated slender abdomen of males, medium sized cymbium with much narrower distal end and elongated semicircular apical lobe of tegulum. *Epocilla* and *Cosmophasis* are included in tribe Chrysillini on the basis of the tegular bump of the palp (Maddison 2015). Our molecular trees corroborated this placement. However, these relationships remain to be further tested when more data become available.

Species diversity is unevenly distributed across the globe with terrestrial biodiversity concentrated in a few restricted biodiversity hotspots, Sri Lanka being one of them (Myers et al. 2000; Sechrest et al. 2002). Sri Lanka’s natural forests are impacted by loss of vegetation and other negative effects of increased human population density. Here, we show that our efforts to mitigate the effects of biodiversity loss might be hampered by the lack of an up-to-date taxonomy. Prior to this study a single species each of *Chrysilla* and *Phintella* was recorded from Sri Lanka. Both were thought to be widespread. This study adds 12 species to that count and demonstrates that some of them have an evolutionary history different from species of *Chrysilla* and *Phintella*. Most of the new taxa are very restricted in distribution. Their conservation needs to be reassessed in light of the new data presented.

## Supplementary Material

XML Treatment for Phintelloides

XML Treatment for Phintelloides alborea

XML Treatment for Phintelloides brunne

XML Treatment for Phintelloides flavoviri

XML Treatment for Phintelloides flavumi

XML Treatment for Phintelloides jesudasi

XML Treatment for Phintelloides orbisa

XML Treatment for Chrysilla

XML Treatment for Chrysilla lauta

XML Treatment for Chrysilla volupe

XML Treatment for Proszynskia

XML Treatment for Proszynskia diatreta

XML Treatment for Phintella

XML Treatment for Phintella argentea

XML Treatment for Phintella jaleeli

XML Treatment for Phintella vittata

## Supplementary materials

Supplementary material 1 Figure S1. Phylogeny of Chrysillini obtained by the ML analysis of CO1 single gene molecular data Data type: phylogeny data Explanation note: Numbers above the line denote bootstrap proportions from 1000 replicates. Only support values above 60% are given. This dataset is made available under the Open Database License (http://opendatacommons.org/licenses/odbl/1.0/). The Open Database License (ODbL) is a license agreement intended to allow users to freely share, modify, and use this Dataset while maintaining this same freedom for others, provided that the original source and author(s) are credited.Nilani Kanesharatnam, Suresh P. Benjamin

Supplementary material 2 Figure S2. Phylogeny of Chrysillini obtained by the ML analysis of 28S single gene molecular data Data type: phylogeny data Explanation note: Numbers above the line denote bootstrap proportions from 1000 replicates. Only support values above 90% are given. This dataset is made available under the Open Database License (http://opendatacommons.org/licenses/odbl/1.0/). The Open Database License (ODbL) is a license agreement intended to allow users to freely share, modify, and use this Dataset while maintaining this same freedom for others, provided that the original source and author(s) are credited.Nilani Kanesharatnam, Suresh P. Benjamin

Supplementary material 3 Figure S3. Phylogeny of Chrysillini obtained by the ML analysis of 18S single gene molecular data Data type: phylogeny data Explanation note: Numbers above the line denote bootstrap proportions from 1000 replicates. Only support values above 70% are given. This dataset is made available under the Open Database License (http://opendatacommons.org/licenses/odbl/1.0/). The Open Database License (ODbL) is a license agreement intended to allow users to freely share, modify, and use this Dataset while maintaining this same freedom for others, provided that the original source and author(s) are credited.Nilani Kanesharatnam, Suresh P. Benjamin
