# Supplementary figures and images for: Multilocus genetic and morphological phylogenetic analysis reveals a radiation of shiny South Asian jumping spiders (Araneae, Salticidae)
Source: Zookeys. 2019 Jan 16;839:1–81. doi: 10.3897/zookeys.839.28312 (PMC6482596; doi:10.3897/zookeys.839.28312)

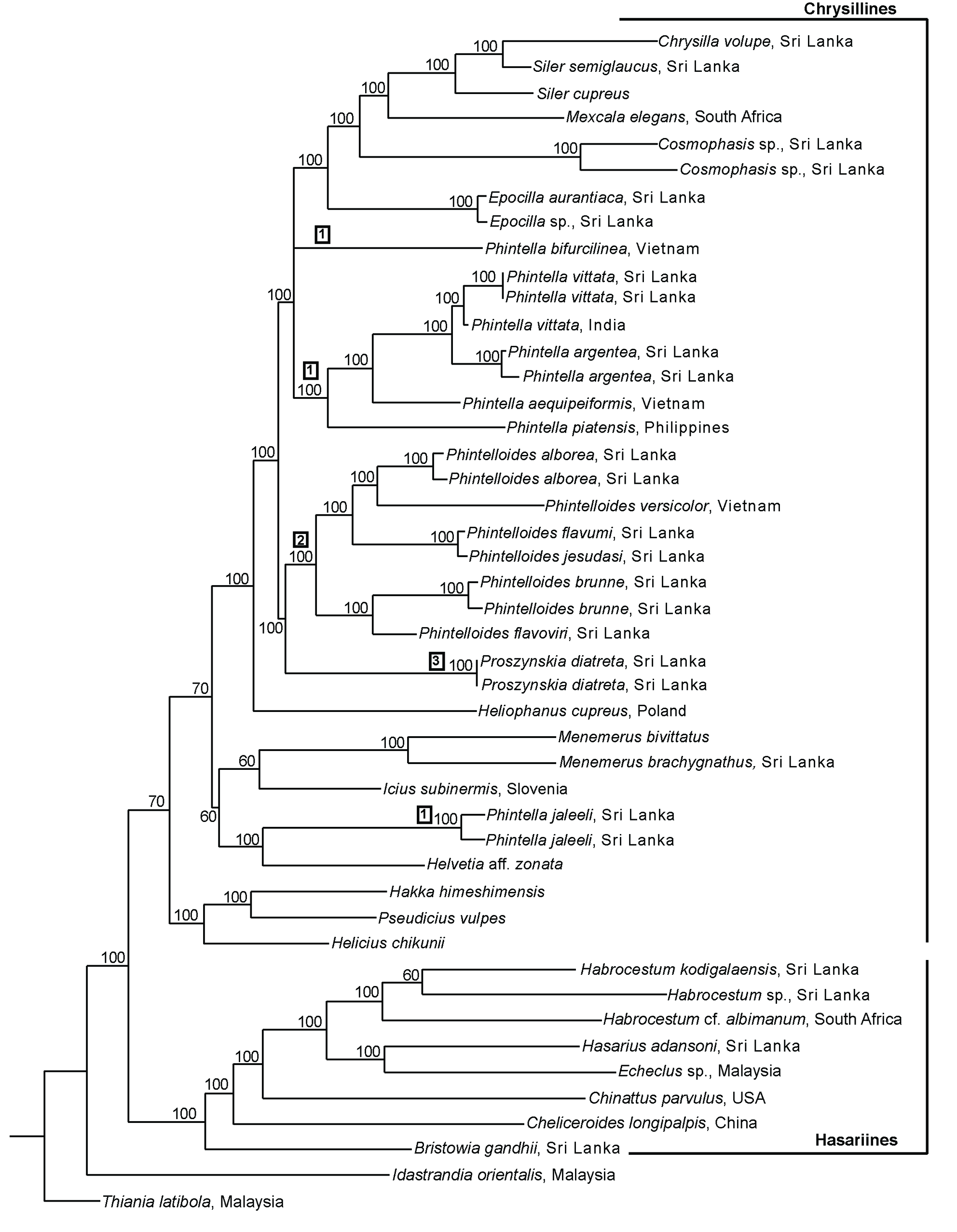

Supplement: Supplementary material 1 [file zookeys-839-001-s001.tif]

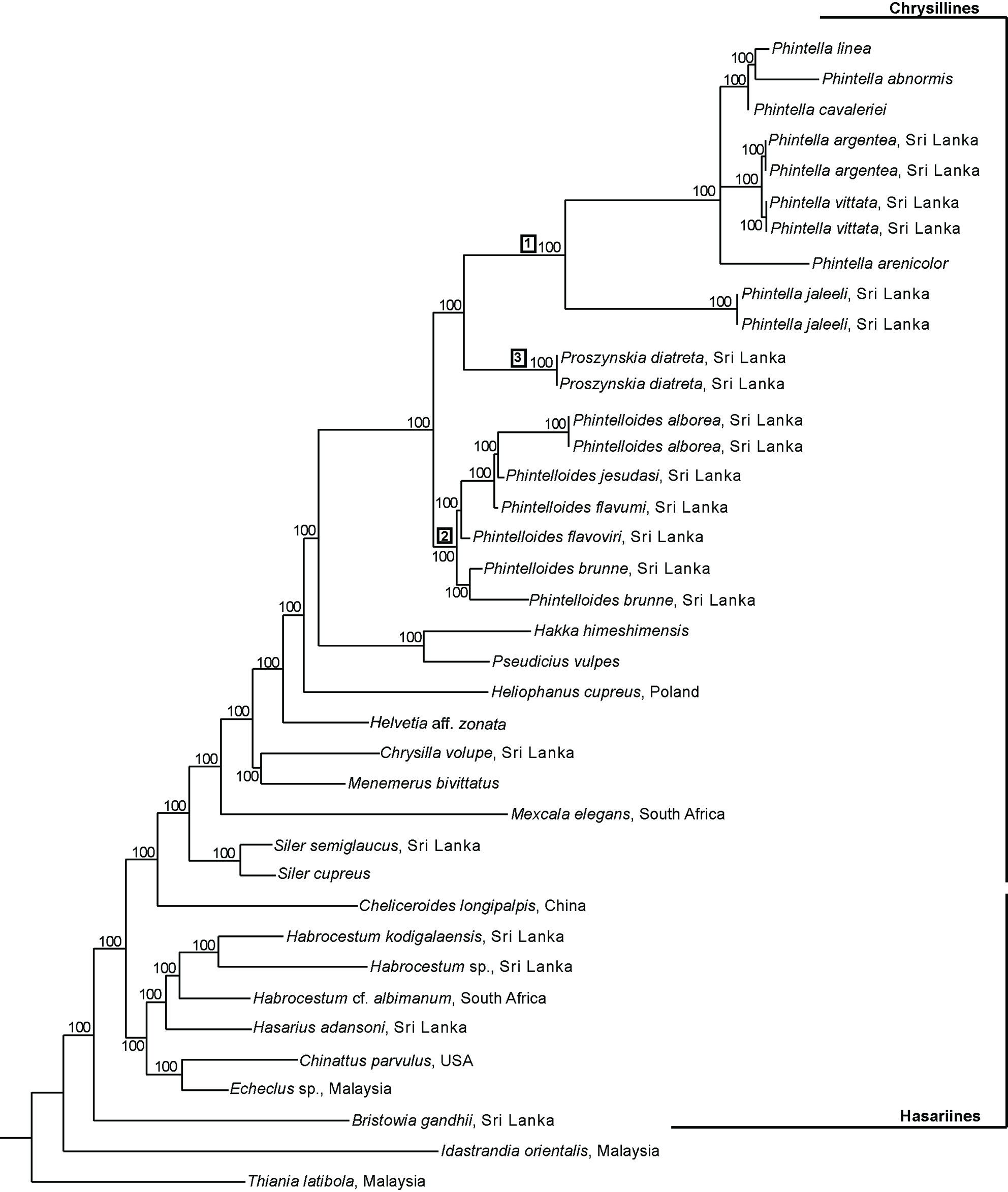

Supplement: Supplementary material 2 [file zookeys-839-001-s002.tif]

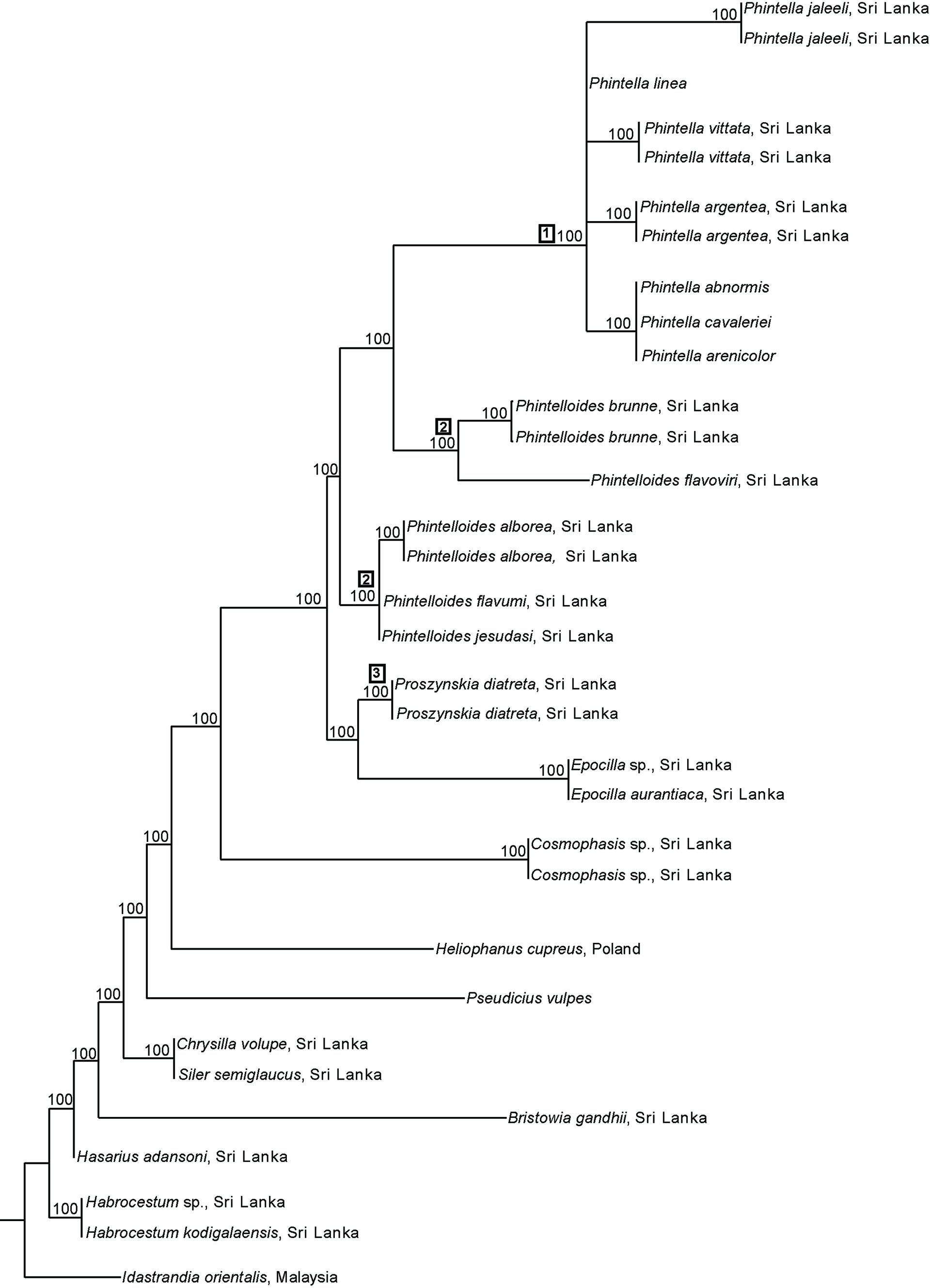

Supplement: Supplementary material 3 [file zookeys-839-001-s003.tif]
